# Pufferfish Optimization Algorithm: A New Bio-Inspired Metaheuristic Algorithm for Solving Optimization Problems

**DOI:** 10.3390/biomimetics9020065

**Published:** 2024-01-23

**Authors:** Osama Al-Baik, Saleh Alomari, Omar Alssayed, Saikat Gochhait, Irina Leonova, Uma Dutta, Om Parkash Malik, Zeinab Montazeri, Mohammad Dehghani

**Affiliations:** 1 Department of Software Engineering, Al-Ahliyya Amman University, Amman 19328, Jordan; o.albaik@ammanu.edu.jo; 2 ISBM COE, Faculty of Science and Information Technology, Software Engineering, Jadara University, Irbid 21110, Jordan; omari08@jadara.edu.jo; 3 Department of Mathematics, Faculty of Science, The Hashemite University, P.O. Box 330127, Zarqa 13133, Jordan; omarm_re@hu.edu.jo; 4 Symbiosis Institute of Digital and Telecom Management, Constituent of Symbiosis International Deemed University, Pune 412115, India; saikat.gochhait@sidtm.edu.in; 5 Neuroscience Research Institute, Samara State Medical University, 89 Chapaevskaya Street, 443001 Samara, Russia; irina.leonova@unn.ru; 6 Faculty of Social Sciences, Lobachevsky University, 603950 Nizhny Novgorod, Russia; 7 Former Dean of Life Sciences and Head of Zoology Department, Celland Molecular Biology, Toxicology Laboratory, Department of Zoology, Cotton University, Guwahati 781001, India; uma.dutta@cottonuniversity.ac.in; 8 Department of Electrical and Software Engineering, University of Calgary, Calgary, AB T2N 1N4, Canada; maliko@ucalgary.ca; 9 Department of Electrical and Electronics Engineering, Shiraz University of Technology, Shiraz 7155713876, Iran; z.montazeri@sutech.ac.ir

**Keywords:** optimization, bio-inspired, metaheuristic, pufferfish, exploration, exploitation

## Abstract

A new bio-inspired metaheuristic algorithm named the Pufferfish Optimization Algorithm (POA), that imitates the natural behavior of pufferfish in nature, is introduced in this paper. The fundamental inspiration of POA is adapted from the defense mechanism of pufferfish against predators. In this defense mechanism, by filling its elastic stomach with water, the pufferfish becomes a spherical ball with pointed spines, and as a result, the hungry predator escapes from this threat. The POA theory is stated and then mathematically modeled in two phases: (i) exploration based on the simulation of a predator’s attack on a pufferfish and (ii) exploitation based on the simulation of a predator’s escape from spiny spherical pufferfish. The performance of POA is evaluated in handling the CEC 2017 test suite for problem dimensions equal to 10, 30, 50, and 100. The optimization results show that POA has achieved an effective solution with the appropriate ability in exploration, exploitation, and the balance between them during the search process. The quality of POA in the optimization process is compared with the performance of twelve well-known metaheuristic algorithms. The simulation results show that POA provides superior performance by achieving better results in most of the benchmark functions in order to solve the CEC 2017 test suite compared to competitor algorithms. Also, the effectiveness of POA to handle optimization tasks in real-world applications is evaluated on twenty-two constrained optimization problems from the CEC 2011 test suite and four engineering design problems. Simulation results show that POA provides effective performance in handling real-world applications by achieving better solutions compared to competitor algorithms.

## 1. Introduction

Optimization problems are a kind of problem that have more than one feasible solution. According to this, optimization is the process of obtaining the best optimal solution among all feasible solutions for an optimization problem [1]. From a mathematical point of view, any optimization problem can be modeled using three parts: decision variables, constraints, and the objective function of the problem. The main goal in optimization is to assign values to the decision variables so that the objective function is optimized by respecting the constraints of the problem [2]. There are numerous optimization problems in science, engineering, mathematics, technology, industry, and real-world applications that must be optimized using appropriate techniques. Problem solving techniques in dealing with optimization problems are classified into two groups: deterministic and stochastic approaches [3]. Deterministic approaches in two classes, gradient-based and non-gradient-based, have effective performance in optimizing convex, linear, continuous, differentiable, and low-dimensional problems [4]. Although, when problems become more complex and especially the dimensions of the problem increase, deterministic approaches are inefficient as they get stuck in local optima [5]. On the other hand, many practical optimization problems have features such as being non-convex, non-linear, discontinuous, non-differentiable, and high dimensions. The disadvantages and ineffectiveness of deterministic approaches in solving practical optimization problems with such characteristics have led researchers to develop stochastic approaches [6].

Metaheuristic algorithms are one of the most effective stochastic approaches for solving optimization problems, which can achieve suitable solutions for optimization problems based on random search in the problem-solving space and the use of random operators and trial and error processes. The optimization process in metaheuristic algorithms is such that the first several candidate solutions are initialized randomly in the problem-solving space under the name of algorithm population. Then, these candidate solutions are improved based on the steps of updating the algorithm population during successive iterations. After the full implementation of the algorithm, the best candidate solution obtained during the algorithm iterations is presented as a solution to the problem [7]. The random search process in the performance of metaheuristic algorithms provides no guarantee to achieving the global optimum, although the solutions obtained from metaheuristic algorithms are acceptable as quasi-optimal because they are close to the global optimum. Achieving more effective solutions closer to the global optimum for optimization problems has been a motivation for researchers to design numerous metaheuristic algorithms [8].

A metaheuristic algorithm, to have an effective search process to achieve a suitable solution for the optimization problem, must be able to search the problem-solving space well at both global and local levels. The goal in global search with the concept of exploration is to comprehensively scan the problem-solving space to avoid getting stuck in local optima and to discover the region containing the main optima. The goal in local search with the concept of exploitation is to scan accurately and with small steps in promising areas in the problem-solving space to achieve better solutions closer to the global optimum. Balancing exploration and exploitation during algorithm iterations and the search process in the problem-solving space is the key point in the success of the metaheuristic algorithm in addition to having a high ability in exploration and exploitation [9].

The main research question according to the numerous metaheuristic algorithms that have been designed so far is the following: “is there still a need to introduce new algorithms or not”? In response to this question, the No Free Lunch (NFL) [10] theorem explains that the successful performance of a metaheuristic algorithm in solving a set of optimization problems is no guarantee that the same algorithm will provide the same performance in solving other optimization problems. Based on the NFL theorem, it cannot be claimed that a particular metaheuristic algorithm is the best optimizer for all optimization applications. This means that a successful algorithm in solving one optimization problem may fail in solving another problem by getting stuck in a local optimum. Hence, there is no assumption of success or failure of implementing a metaheuristic algorithm on an optimization problem. The NFL theorem, by keeping the studies of metaheuristic algorithms active, motivates researchers to provide more effective solutions to optimization problems by designing new metaheuristic algorithms.

The innovation and novelty of this paper is in introducing a new metaheuristic algorithm called the Pufferfish Optimization Algorithm (POA) to solve optimization problems in different sciences. The scientific contributions of this study are as follows:

- POA is designed based on simulating the natural behavior of pufferfish and its predators in the sea.
- The basic inspiration of POA is taken from the defense mechanism of pufferfish against predator attacks.
- The theory of POA is stated and its implementation steps are mathematically modeled in two phases: (i) exploration based on the simulation of the predator’s attack on the pufferfish and (ii) exploitation based on the simulation of the pufferfish’s defense mechanism against the predator.
- The performance of POA is evaluated to optimize the CEC 2017 test suite for problem dimensions of 10, 30, 50, and 100.
- The effectiveness of POA in handling optimization tasks is evaluated on twenty-two constrained optimization problems from the CEC 2011 test suite and four engineering design problems.
- Results obtained from POA are compared with the performance of twelve well-known metaheuristic algorithms.

The structure of the paper is as follows: The literature review is presented in Section 2. Then, the proposed Pufferfish Optimization Algorithm is introduced and modeled in Section 3. Simulation studies and results are presented in Section 4. The effectiveness of POA in solving real-world applications is investigated in Section 5. Conclusions and suggestions for future research are provided in Section 6.

## 2. Literature Review

Metaheuristic algorithms have been developed by taking inspiration from various natural phenomena, lifestyles of living organisms, concepts in biological, genetics, physics sciences, rules of games, human interactions, and other evolutionary phenomena. According to the employed inspiration source in the design, metaheuristic algorithms are placed in five groups: swarm-based, evolutionary-based, physics-based, human-based, and game-based approaches.

Swarm-based metaheuristic algorithms are developed with inspiration from the natural behavior and strategies of animals, insects, birds, reptiles, aquatics, and other living creatures in the wild. Particle Swarm Optimization (PSO) [11], Ant Colony Optimization (ACO) [12], Artificial Bee Colony (ABC) [13], and Firefly Algorithm (FA) [14] are among the most well-known swarm-based metaheuristic algorithms. PSO is designed based on modeling the movement of flocks of birds and swarms of fish that are searching for food. ACO is proposed based on modeling the ability of ants to explore the shortest communication path between the food source and the colony. ABC is introduced based on the modeling of the hierarchical activities of honeybees in an attempt to reach new food sources. FA is designed with inspiration from optical communication between fireflies. Pelican Optimization (PO) is another swarm-based metaheuristic algorithm, that is inspired by the strategy of pelicans during hunting [15]. Among the natural behavior of living organisms in the wild, the processes of hunting, foraging, chasing, digging, and migration are much more prominent and have been a source of inspiration in the design of swarm-based metaheuristic algorithms such as the Snake Optimizer (SO) [16], Sea Lion Optimization (SLnO) [17], Flying Foxes Optimization (FFO) [18], Mayfly Algorithm (MA) [19], White Shark Optimizer (WSO) [20], African Vultures Optimization Algorithm (AVOA) [21], Grey Wolf Optimizer (GWO) [22], Reptile Search Algorithm (RSA) [23], Whale Optimization Algorithm (WOA) [24], Golden Jackal Optimization (GJO) [25], Honey Badger Algorithm (HBA) [26], Marine Predator Algorithm (MPA) [27], Orca Predation Algorithm (OPA) [28], and Tunicate Swarm Algorithm (TSA) [29].

Evolutionary-based metaheuristic algorithms are developed with inspiration from the concepts of biology and genetics, natural selection, survival of the fittest, and Darwin’s evolutionary theory. The Genetic Algorithm (GA) [30] and Differential Evolution (DE) [31] are the most well-known algorithms of this group, whose design is inspired by the reproduction process, genetic concepts, and the use of random mutation, selection, and crossover operators. The Artificial Immune System (AIS) is introduced based on the simulation of the mechanism of the body’s defense system against diseases and microbes [32]. Some other evolutionary-based metaheuristic algorithms are the Cultural Algorithm (CA) [33], Genetic Programming (GP) [34], and Evolution Strategy (ES) [35].

Physics-based metaheuristic algorithms are developed with inspiration from laws, transformations, processes, phenomena, forces, and other concepts in physics. Simulated Annealing (SA) is one of the most well-known physics-based metaheuristic algorithms, which is developed based on the modeling of the metal annealing phenomenon. In this process, with the aim of achieving an ideal crystal, metals are first melted under heat, then slowly cooled [36]. Physical forces and Newton’s laws of motion have been fundamental inspirations in designing algorithms such as the Gravitational Search Algorithm (GSA) based on gravitational attraction force [37], the Momentum Search Algorithm (MSA) [38] based on momentum force, and the Spring Search Algorithm (SSA) [39] based on the elastic force of a spring. The Water Cycle Algorithm (WCA) is proposed based on the modeling of physical transformations in the natural water cycle [40]. Some other physics-based metaheuristic algorithms are Fick’s Law Algorithm (FLA) [41], Prism Refraction Search (PRS) [42], Henry Gas Optimization (HGO) [43], Black Hole Algorithm (BHA) [44], Nuclear Reaction Optimization (NRO) [45], Equilibrium Optimizer (EO) [46], Multi-Verse Optimizer (MVO) [47], Lichtenberg Algorithm (LA) [48], Archimedes Optimization Algorithm (AOA) [49], Thermal Exchange Optimization (TEO) [50], and Electro-Magnetism Optimization (EMO) [51].

Human-based metaheuristic algorithms are developed with inspiration from the thoughts, choices, decisions, interactions, communications, and other activities of humans in society or personal life. The Teaching–Learning-Based Optimization (TLBO) is one of the most widely used human-based metaheuristic algorithms, whose design is inspired by educational communication and knowledge exchange between teachers and students, as well as students with each other [52]. The Mother Optimization Algorithm (MOA) is introduced with inspiration from Eshrat’s care of her children [6]. The Election-Based Optimization Algorithm (EBOA) is proposed based on modeling the process of voting and holding elections in society [8]. The Chef-Based Optimization Algorithm (CHBO) is designed based on the simulation of teaching cooking skills by chefs to applicants in culinary schools [53]. The Teamwork Optimization Algorithm (TOA) is developed with the inspiration of collaboration among team members in providing teamwork in order to achieve specified team goals [54]. Some other human-based metaheuristic algorithms are Driving Training-Based Optimization (DTBO) [5], War Strategy Optimization (WSO) [55], Ali Baba and the Forty Thieves (AFT) [56], Gaining Sharing Knowledge-based Algorithm (GSK) [57], and Coronavirus Herd Immunity Optimizer (CHIO) [58].

Game-based metaheuristic algorithms are developed by taking inspiration from the rules of games as well as the behavior of players, coaches, referees, and other influential people in individual and team games. The Darts Game Optimizer (DGO) is one of the most well-known algorithms of this group, which is proposed based on modeling the competition of players in throwing darts and collecting more points in order to win the game [59]. The Golf Optimization Algorithm (GOA) is introduced based on the simulation of players hitting the ball in order to place the ball in the holes [60]. The Puzzle Algorithm (PA) is designed based on modeling the strategy of players putting puzzle pieces together in order to complete it according to the pattern [61]. Some other game-based metaheuristic algorithms are Volleyball Premier League (VPL) [62], Running City Game Optimizer (RCGO) [63], and Tug of War Optimization (TWO) [64].

Based on the best knowledge obtained from the literature review, no metaheuristic algorithm inspired by the natural behavior of pufferfish in the wild has been introduced so far. Meanwhile, the attack of the hungry predator on the pufferfish and the defense mechanism of the pufferfish against the attacks of the predators are intelligent processes that can be the basis for the design of a new optimizer. To address this research gap in the studies of metaheuristic algorithms, a new bio-inspired metaheuristic algorithm, based on the modeling of natural behavior between pufferfish and their predators, has been designed and is described in the next section.

## 3. Pufferfish Optimization Algorithm

In this section, the inspiration source in the design of the proposed Pufferfish Optimization Algorithm approach is stated first, then its implementation steps are mathematically modeled to be used to solve optimization problems.

### 3.1. Inspiration of POA

**Figure 1:**
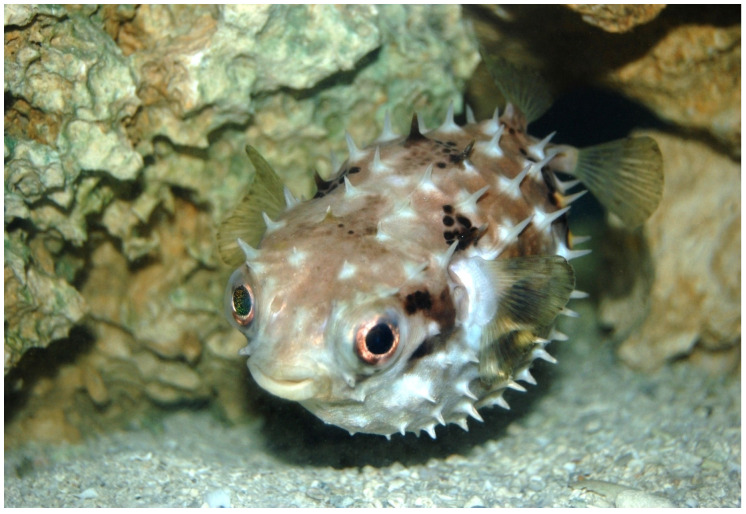
Pufferfish taken from free media Wikimedia Commons.

Pufferfish are a primarily marine and estuarine fish of the family Tetraodontidae and order Tetraodontiformes. This fish is morphologically similar to porcupinefish that have large spines. The body size of pufferfish is small to medium and their maximum length has been observed up to 50 cm [65]. Their beak-like four teeth are one of the most characteristic features of pufferfish. The lack of pectoral fins, pelvis, and ribs are also unique to pufferfish. The significantly lost fin and bone features of the pufferfish are due to the fish’s specialized defense mechanism, which extends by sucking water through the mouth cavity [66]. An image of the pufferfish is shown in Figure 1.

Pufferfish have a very slow movement, which makes them an easy target for predators. The pufferfish’s specialized defense mechanism against predator attacks is to fill its elastic stomach with water until it becomes a large, spherical, spiny ball. The pointed spines of pufferfish make the hungry predator face a ball of pointed spines instead of an easy meal. Predators, after encountering this warning, realize the danger and move away from the pufferfish [66].

Among the natural behaviors of pufferfish, conflicts between this fish and predators and the use of the defense mechanism of turning into a ball of pointed spines against the attacks of predators are much more significant. The modeling of these natural processes, which consists of (i) a predator’s attack on pufferfish and (ii) a pufferfish’s defense mechanism against predator attacks, is employed in the design of the proposed POA approach discussed below.

### 3.2. Algorithm Initialization

The proposed POA approach is a population-based technique that can achieve effective solutions for optimization problems by using its population search power in the problem-solving space in an iteration-based process. Each POA member determines the values for the decision variables of the problem according to its position in the search space. Therefore, each POA member is a candidate solution to the problem that can be modeled from a mathematical point of view using a vector, where each element of this vector corresponds to a decision variable. POA members together form the population of the algorithm. From a mathematical point of view, the community of these vectors can be modeled using a matrix according to Equation (1). The primary position of each POA member at the beginning of the algorithm is initialized using Equation (2).

(1) $$X={\left[\begin{array}{c} X_{1}\\ \vdots \\ X_{i}\\ \vdots \\ X_{N} \end{array}\right]}_{N\times m}={\left[\begin{array}{ccccc} x_{1,1} & \cdots & x_{1,d} & \cdots & x_{1,m}\\ \vdots & \ddots & \vdots & ⋰ & \vdots \\ x_{i,1} & \cdots & x_{i,d} & \cdots & x_{i,m}\\ \vdots & ⋰ & \vdots & \ddots & \vdots \\ x_{N,1} & \cdots & x_{N,d} & \cdots & x_{N,m} \end{array}\right]}_{N\times m},$$

(2) $$x_{i,d}=lb_{d}+r\cdot (ub_{d}-lb_{d}),$$

Here, X is the POA population matrix, $X_{i}$ is the ith POA member (candidate solution), $x_{i,d}$ is its dth dimension in the search space (decision variable), N is the number of population members, m is the number of decision variables, r is a random number in the interval $\left[0,\;1\right]$, and $lb_{d}$ and $ub_{d}$ are the lower bound and upper bound of the dth decision variable, respectively.

With each POA member as a candidate solution for the problem, the objective function of the problem can be evaluated. The set of evaluated values for the objective function of the problem can be represented using a vector according to Equation (3).

(3) $$F={\left[\begin{array}{c} F_{1}\\ \vdots \\ F_{i}\\ \vdots \\ F_{N} \end{array}\right]}_{N\times 1}={\left[\begin{array}{c} F(X_{1})\\ \vdots \\ F(X_{i})\\ \vdots \\ F(X_{N}) \end{array}\right]}_{N\times 1}$$

Here, F is the vector of the evaluated objective function and $F_{i}$ is the evaluated objective function based on the ith POA member.

The evaluated values for the objective function are suitable criteria to measure the quality of candidate solutions proposed by each POA member. The best evaluated value for the objective function corresponds to the best member (i.e., the best candidate solution) and the worst evaluated value for the objective function corresponds to the worst member (i.e., the worst candidate solution). Considering that the position of POA members in the problem-solving space is updated in each iteration, the best member should also be updated in each iteration based on the comparison of new evaluated values for the objective function.

### 3.3. Mathematical Modelling of POA

In the design of the proposed POA approach, the position of population members in the problem-solving space is updated based on the simulation of natural behaviors between pufferfish and its predators. In this natural process, the predator first attacks the pufferfish. Then, the pufferfish uses its defense mechanism and turns into a ball of pointed spines, leading to the threat and escape of the predator. Therefore, in each iteration, the position of POA population members is updated in two phases: (i) exploration based on the simulation of the predator’s attack towards the pufferfish and (ii) exploitation based on the simulation of the defense mechanism of the pufferfish against the predator.

#### 3.3.1. Phase 1: Predator Attack towards Pufferfish (Exploration Phase)

In the first phase of POA, the position of the population members is updated based on the simulation of the predator attack strategy towards the pufferfish. Because of its slow speed, pufferfish are easy prey for hungry hunters. The position change of the predator during the attack towards the pufferfish is simulated to update the position of the POA members in the problem-solving space. Modeling the movement of the predator towards the pufferfish leads to extensive changes in the position of the POA members and as a result increases the exploration power of the algorithm for global search.

In POA design for each population member as a predator, the position of other population members that have a better value for the objective function is considered as the position of the candidate pufferfish for attack. The set of pufferfish for each population member is identified using Equation (4).

(4) $$CP_{i}=\left\{X_{k}:F_{k}<F_{i}\;and\;k\neq i\right\},\;where\;i=1,\;2,\;\ldots ,\;N\;and\;k\in \left\{1,\;2,\;\ldots ,\;N\right\},$$

Here, $CP_{i}$ is the set of candidate pufferfish locations for the ith predator, $X_{k}$ is the population member with a better objective function value than the ith predator, and $F_{k}$ is its objective function value.

In the design of POA, it is assumed that among the candidate pufferfish determined in the CP set, the predator selects a pufferfish completely randomly, which is considered as the selected pufferfish (SP). Based on the modeling of the movement of the predator towards the pufferfish, a new position in the problem-solving space is calculated for each POA member using Equation (5). Then, if the objective function value is improved in the new position, this new position replaces the previous position of the corresponding member according to Equation (6).

(5) $$x_{i,j}^{P1}=x_{i,j}+r_{i,j}\cdot (SP_{i,j}-I_{i,j}\cdot x_{i,j}),\;\;$$

(6) $$X_{i}=\left\{\begin{array}{c} X_{i}^{P1},\;\;F_{i}^{P1}\leq F_{i};\\ X_{i},\;\;else\;, \end{array}\right.$$

Here, $SP_{i}$ is the selected pufferfish for the ith predator selected randomly from the $CP_{i}$ set (i.e., $SP_{i}$ is an element of the $CP_{i}$ set), $SP_{i,j}$ is its jth dimension, $X_{i}^{P1}$ is the new position calculated for the ith predator based on first phase of the proposed POA, $x_{i,j}^{P1}$ is its jth dimension, $F_{i}^{P1}$ is its objective function value, $r_{i,j}\;$are random numbers from the interval $\left[0,\;1\right]$, and $I_{i,j}$ are numbers which are randomly selected as 1 or 2.

#### 3.3.2. Phase 2: Defense Mechanism of Pufferfish against Predators (Exploitation Phase)

In the second phase of POA, the position of population members is updated based on the simulation of a pufferfish’s defense mechanism against predator attacks. When a pufferfish is attacked by a predator, it turns into a ball of pointed spines by filling its very elastic stomach with water. In this situation, the predator who faced such a warning instead of an easy meal runs away from the position of the pufferfish. Modeling the predator moving away from the pufferfish leads to small changes in the position of the POA members and as a result increases the exploitation power of the algorithm for local search.

Based on the modeling of the predator’s position change when moving away from the predator, a new position is calculated for each POA member using Equation (7). Then, this new position, if it improves the value of the objective function, replaces the corresponding member according to Equation (8).

The reason for using Equation (8) is that in POA design, effort has been made to improve the algorithm. In fact, when a new position is calculated for the POA member, it is checked from a comparison of the objective function values whether this new position for the corresponding member leads to a better solution to the problem or not. If the answer is positive, the new position is acceptable for the corresponding POA member, otherwise the new position is inappropriate (because it leads to a weaker solution) and the corresponding member remains in the previous position. Therefore, Equation (8) shows that the update process for each POA member is conditional on improving the value of the objective function.

(7) $$x_{i,j}^{P2}=x_{i,j}+\left(1-2\;r_{i,j}\right)\cdot \;\frac{ub_{j}-lb_{j}}{t},$$

(8) $$X_{i}=\left\{\begin{array}{c} X_{i}^{P2},\;\;F_{i}^{P2}\leq F_{i};\\ X_{i},\;\;else\;, \end{array}\right.$$

Here, $X_{i}^{P2}$ is the new position calculated for the ith predator based on the second phase of the proposed POA, $x_{i,j}^{P2}$ is its jth dimension, $F_{i}^{P2}$ is its objective function value, $r_{i,j}$ are random numbers from the interval $\left[0,\;1\right]$, and t is the iteration counter.

### 3.4. Repetition Process, Pseudocode, and Flowchart of POA

**Figure 2:**
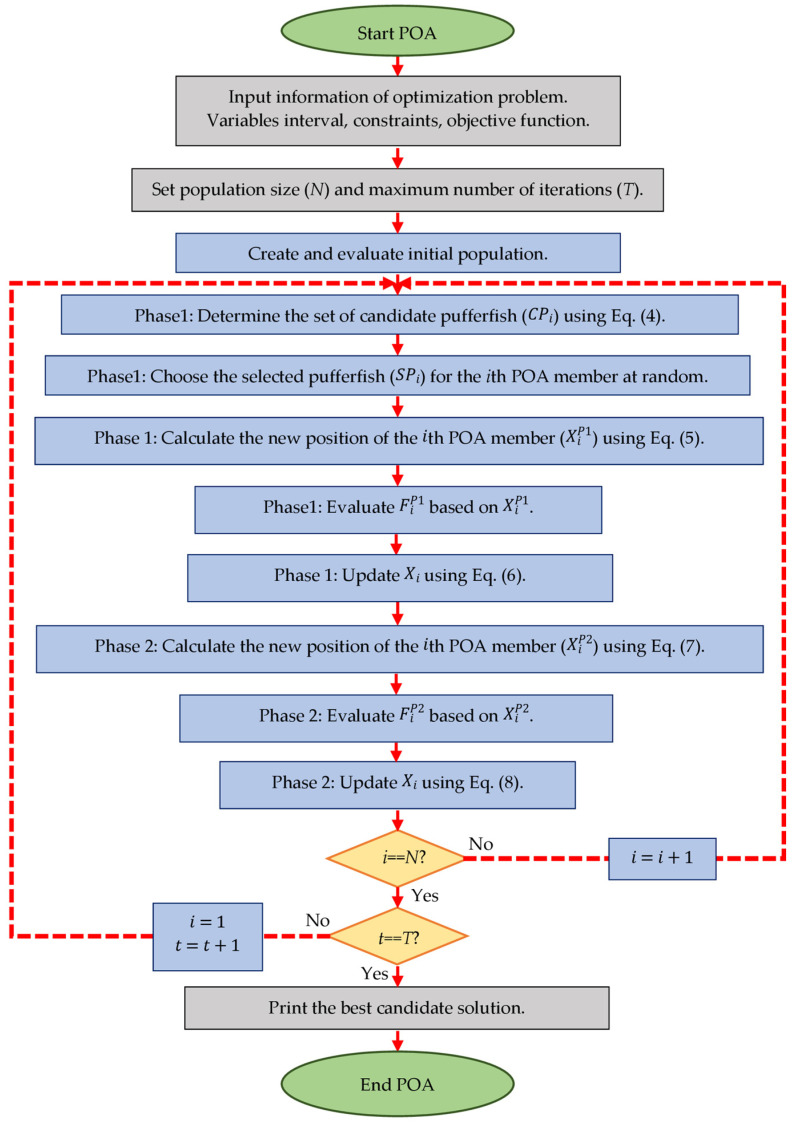
Flowchart of POA.

By updating the position of all POA members based on the exploration and exploitation phases, the first iteration of the algorithm is completed. After that, the algorithm enters the next iteration and the process of updating the position of POA members continues using Equations (4) through (8) until the last iteration of the algorithm. In each iteration, the position of the best POA member is updated and stored based on the comparison of the evaluated values for the objective function. At the end of the full implementation of the algorithm, the position of the best POA member is presented as a solution to the problem. The implementation steps of POA are shown as a flowchart in Figure 2 and its pseudocode is presented in Algorithm 1.


**Algorithm 1.** Pseudocode of POA.Start POA.1:Input problem information: variables, objective function, and constraints.2:Set POA population size (*N*) and iterations (*T*).3:Generate the initial population matrix at random using Equation (2). $x_{i,d}\leftarrow lb_{d}+r\cdot (ub_{d}-lb_{d})$.4:Evaluate the objective function.5:
For t=1 to *T*6:
For i=1 to N7:
Phase 1: Predator attack towards pufferfish (exploration phase).8:

Determine the candidate pufferfish set for the *i*th POA member using Equation (4). $CP_{i}\leftarrow \left\{X_{k_{i}}:F_{k_{i}}<F_{i}\;and\;k_{i}\neq i\right\}$.9:

Select the target pufferfish for the *i*th POA member at random.10:

Calculate new position of *i*th POA member using Equation (5). $x_{i,d}^{P1}\leftarrow x_{i,d}+r\cdot \left(SP_{i,d}-I\cdot x_{i,d}\right)$.11:

Update *i*th POA member using Equation (6). $X_{i}\leftarrow \left\{\begin{array}{c} X_{i}^{P1},\;\;F_{i}^{P1}<F_{i};\\ X_{i},\;\;else. \end{array}\right.$12:
Phase 2: Defense mechanism of pufferfish against predators (exploitation phase).13:

Calculate new position of *i*th POA member using Equation (7). $x_{i,d}^{P2}\leftarrow x_{i,d}+(1-2r)\cdot \frac{\left(ub_{d}-lb_{d}\right)}{t}$.14:

Update *i*th POA member using Equation (8). $X_{i}\leftarrow \left\{\begin{array}{c} X_{i}^{P2},\;\;F_{i}^{P2}<F_{i};\\ X_{i},\;\;else. \end{array}\right.$15:
end16:

Save the best candidate solution so far.17:
end 18: Output the best quasi-optimal solution obtained with the POA.End POA.


### 3.5. POA for Handling the Constrained Problems

Many practical optimization problems are constrained problems that can be solved using metaheuristic algorithms. To apply POA in this type of optimization problem, two strategies have been considered: (i) replacing the inappropriate solution with a feasible solution that is randomly generated with respect to the constraints and (ii) using the penalty coefficient.

In the first case, when the constraints of the problem are not met after updating a solution, this solution is completely removed from the algorithm population and a new feasible solution is generated randomly and replaces that inappropriate solution.

In the second case, in the case of an inappropriate solution that does not meet the constraints of the problem, the objective function corresponding to that inappropriate solution is added with a penalty amount, and as a result, this solution is automatically recognized by the algorithm as a non-optimal solution.

### 3.6. Computational Complexity of POA

In this subsection, the computational complexity of the proposed POA approach is analyzed. The preparation and initialization steps of POA have a computational complexity equal to *O*(*Nm*), where *N* is the number of POA population members and *m* is the number of decision variables of the problem. In POA design, in each iteration, the position of population members is updated in two phases. Therefore, the POA update process has a computational complexity equal to *O*(*2NmT*), where *T* is the maximum number of iterations of the algorithm. According to this, the total computational complexity of the proposed POA approach is equal to *O*(*Nm*(*1* + *2T*)). If fixed numbers are ignored, the computational complexity of POA can be deduced to be *O*(*NmT*).

## 4. Simulation Studies and Results

In this section, the performance of the proposed POA approach to solve optimization problems is evaluated. In this regard, POA is implemented to handle the CEC 2017 test suite for problem dimensions equal to 10, 30, 50, and 100.

### 4.1. Performance Comparison

The performance quality of POA in solving optimization problems has been compared with the performance of twelve well-known metaheuristic algorithms: GA [30], PSO [11], GSA [37], TLBO [52], MVO [47], GWO [22], WOA [24], MPA [27], TSA [29], RSA [23], AVOA [21], and WSO [20]. The values of the control parameters of the metaheuristic algorithms are given in Table 1. Simulations are implemented in the software MATLAB R2022a using a 64-bit Core i7 processor with 3.20 GHz and 16 GB main memory. The implementation results of the metaheuristic algorithms on the benchmark functions are reported with six statistical indicators: mean, best, worst, standard deviation (std), median, and rank. The values obtained for the mean index have been used as criteria in the ranking of metaheuristic algorithms in handling each of the benchmark functions.

### 4.2. Evaluation CEC 2017 Test Suite

In this subsection, the performance of POA and competitor algorithms in handling the CEC 2017 test suite is evaluated. The CEC 2017 test suite has thirty standard benchmark functions consisting of (i) three unimodal functions of C17-F1 to C17-F3, (ii) seven multimodal functions of C17-F4 to C17-F10, (iii) ten hybrid functions of C17-F11 to C17-F20, and (iv) ten composition functions of C17-F21 to C17-F30. Among these functions, C17-F2 is excluded from the simulation calculations due to its unstable behavior. A full description, details, and more information about the CEC 2017 test suite is available in [67].

**Figure 3:**
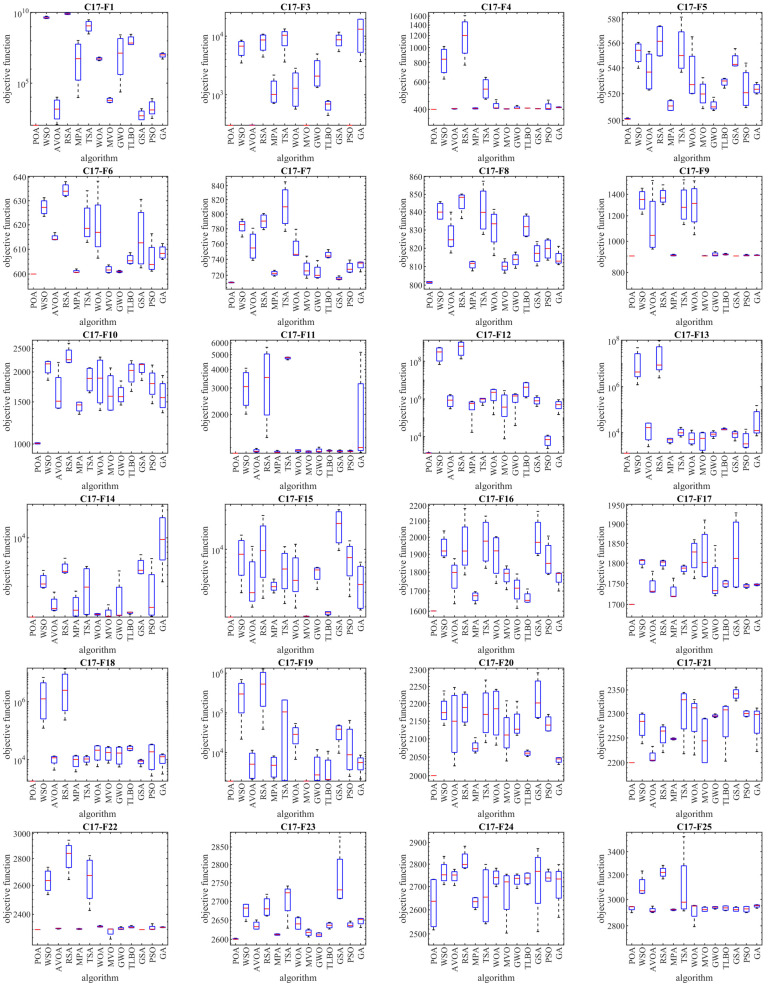

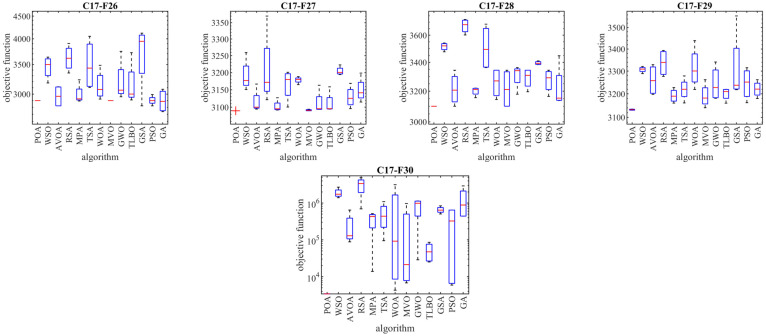
Boxplot diagrams of POA and competitor algorithms’ performances on CEC 2017 test suite (dimension = 10).

**Figure 4:**
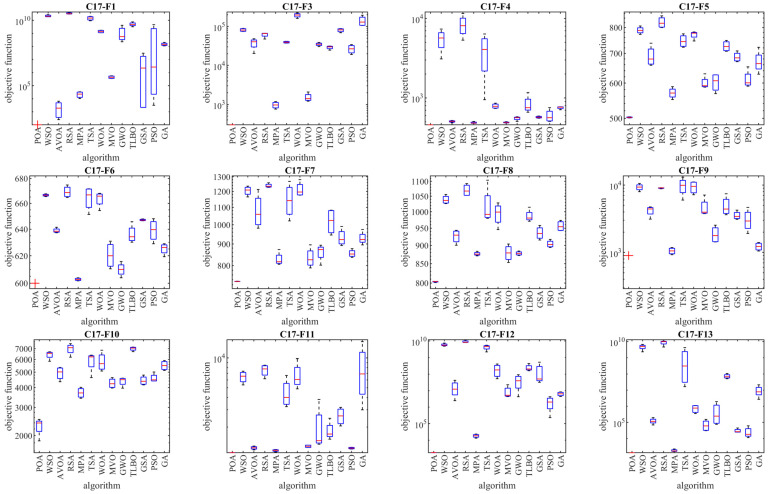

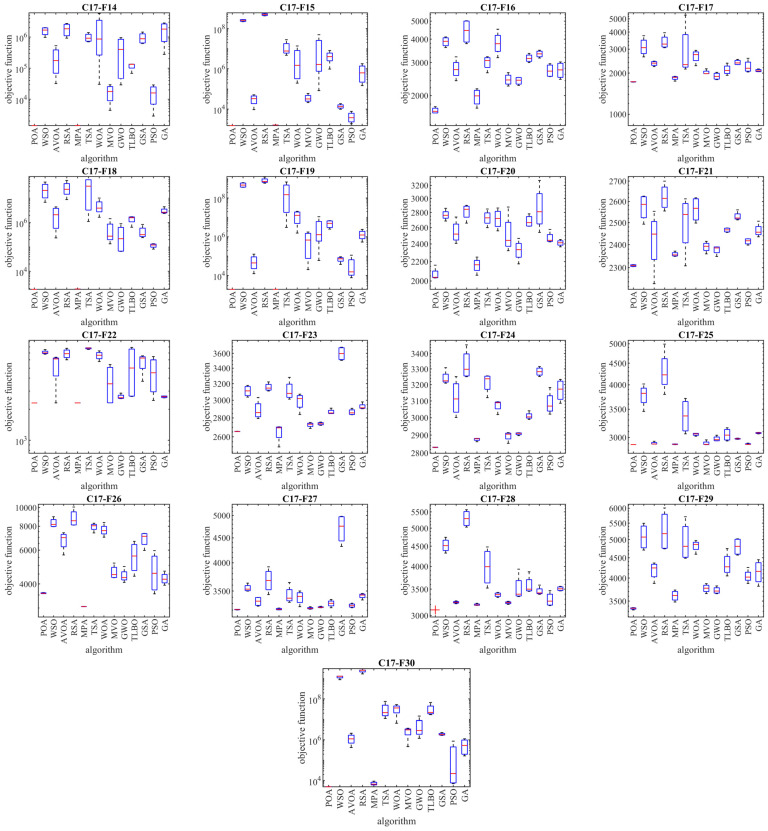
Boxplot diagrams of POA and competitor algorithms’ performances on CEC 2017 test suite (dimension = 30).

**Figure 5:**
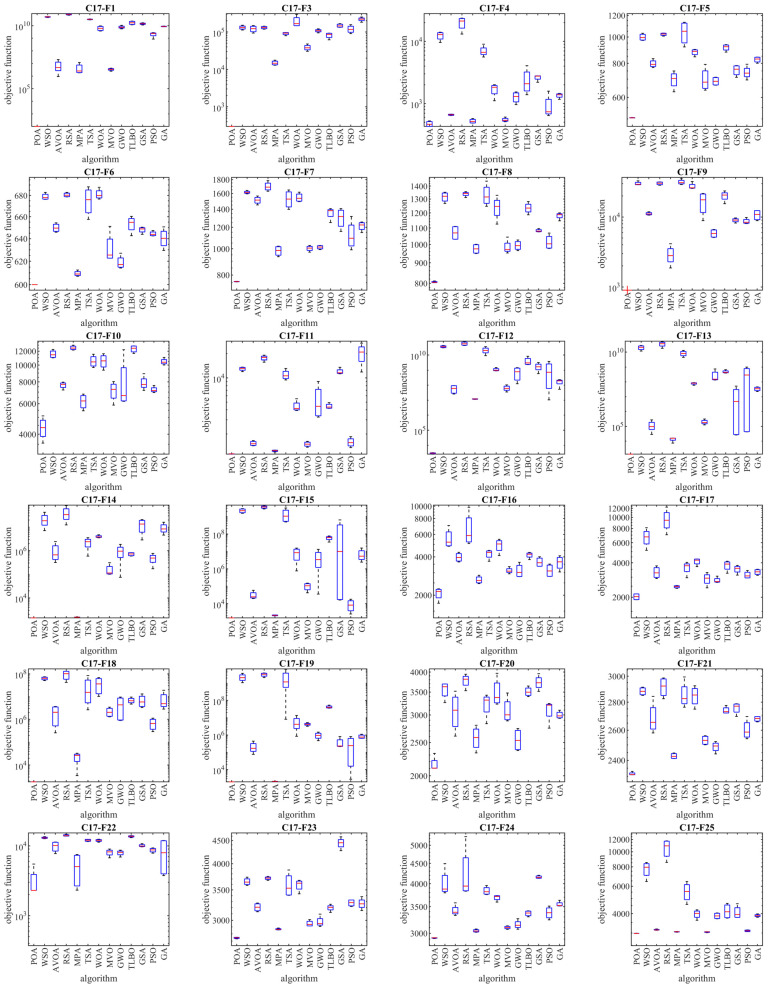

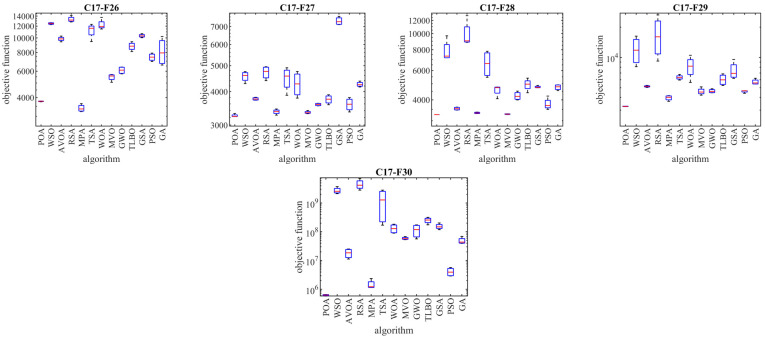
Boxplot diagrams of POA and competitor algorithms’ performances on CEC 2017 test suite (dimension = 50).

**Figure 6:**
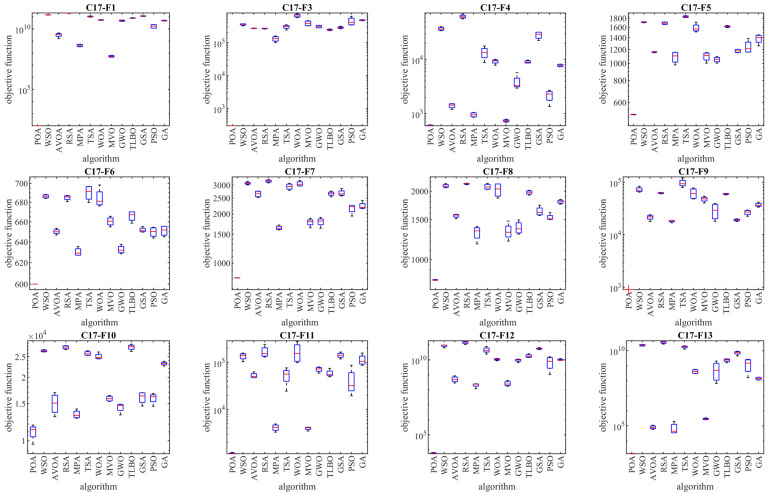

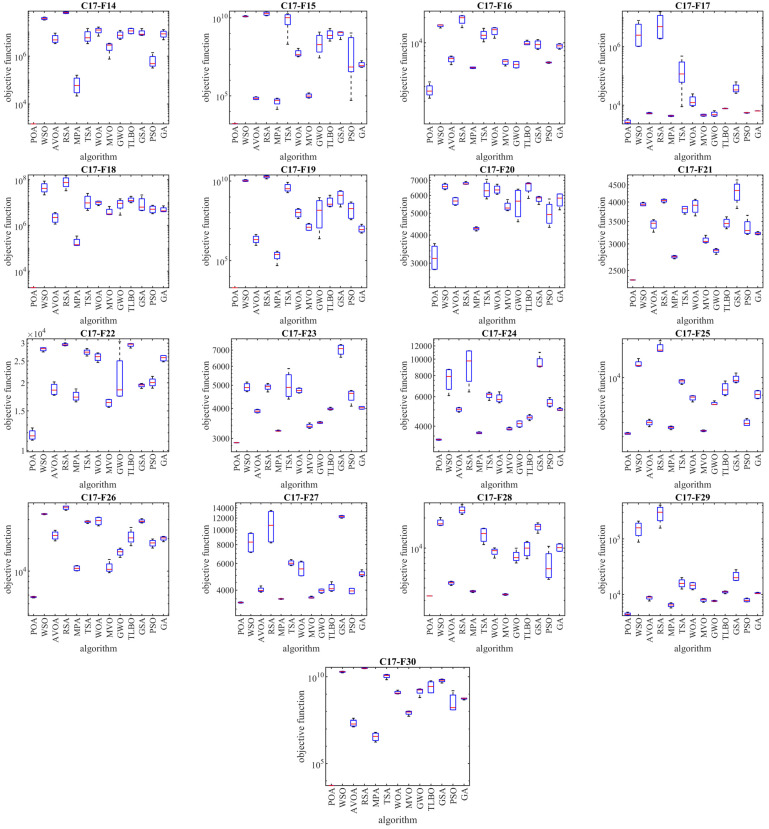
Boxplot diagrams of POA and competitor algorithms’ performances on CEC 2017 test suite (dimension = 100).

The results of employing POA and competitor algorithms to optimize the CEC 2017 test suite are reported in Table 2, Table 3, Table 4 and Table 5. The boxplot diagrams resulting from the performance of metaheuristic algorithms are plotted in Figure 3, Figure 4, Figure 5 and Figure 6. What is evident from the optimization results, in handling the CEC 2017 test suite for the problem dimension equal to 10 (*m* = 10), is that POA is the first best optimizer for the following functions: C17-F1, C17-F3 to C17-F21, C17-F23, C17-F24, and C17-F27 to C17-F30. For the problem dimension equal to 30 (*m* = 30), the proposed POA approach is the first best optimizer for the following functions: C17-F1, C17-F3 to C17-F22, C17-F24, C17-F25, and C17-F27 to C17-F30. For the problem dimension equal to 50 (*m* = 50), the proposed POA approach is the first best optimizer for the following functions: C17-F1, C17-F3 to C17-F25, and C17-F27 to C17-F30. For the problem dimension equal to 100 (*m* = 100), the proposed POA approach is the first best optimizer for the following functions: C17-F1 and C17-F3 to C17-F30.

Based on the optimization results, POA has been able to achieve effective solutions for the benchmark functions with a high ability in exploration, exploitation, and the balance between them during the search process in the problem-solving space. Simulation results show that the proposed POA approach, by providing better results in most of the benchmark functions and getting the rank of the first best optimizer, has provided superior performance compared to competitor algorithms in order to handle the CEC 2017 test suite for problem dimensions equal to 10, 30, 50, and 100.

As described, the CEC 2017 test suite consists of thirty standard benchmark functions of various types. The unimodal functions C17-F1 and C17-F3 have only one global optimal solution without having any local optimum solutions. For this reason, unimodal functions are suitable criteria to evaluate the exploitation ability of metaheuristic algorithms. The findings obtained from the simulation results show that the proposed POA approach has a higher ability in exploitation for local search management by providing better results compared to competing algorithms. Multimodal functions C17-F4 to C17-F10 have several local optimal solutions in addition to the global optimum. For this reason, multimodal functions challenge the ability of metaheuristic algorithms in exploration and global search. The simulation findings of the performance of metaheuristic algorithms on functions C17-F4 to C17-F10 show that the proposed POA approach with a high exploration ability to manage the global search in the problem-solving space has provided superior performance compared to competing algorithms.

Hybrid functions C17-F11 to C17-F20 and composition functions C17-F21 to C17-F30 are suitable criteria for evaluating the ability of metaheuristic algorithms to balance exploration and exploitation during the search process in the problem-solving space. The simulation results of functions C17-F11 to C17-F30 show that the proposed POA approach with its high ability in balancing exploration and exploitation has been able to provide superior performance compared to competing algorithms. The findings obtained from the performance of the proposed POA approach and competing algorithms on the CEC 2017 test suite for problem dimensions equal to 10, 30, 50, and 100 confirm that POA has a higher ability in exploration, exploitation, and balancing them during the search process compared to competing algorithms.

The analysis of the boxplot diagrams intuitively shows that POA has been able to provide better solutions in most of the benchmark functions compared to competing algorithms. Comparing the height of the boxplot charts provides appropriate information about the standard deviation. Examining this issue shows how the results were scattered in independent performances. Therefore, what can be concluded from the intuitive analysis of the boxplot diagrams is that POA has provided better results and lower standard deviation in most of the benchmark functions, compared to competing algorithms, in handling the CEC 2017 test suite.

### 4.3. Statistical Analysis

In this subsection, using statistical analysis on the results obtained from metaheuristic algorithms, it has been checked whether the superiority of POA against competitor algorithms is significant from a statistical point of view. For this purpose, the Wilcoxon rank sum test [68] is employed, which is a non-parametric statistical test and is used to determine the significant difference between the means of two data samples. In this test, the presence or absence of a significant difference is determined using a criterion called the *p*-value.

The implementation results of the Wilcoxon rank sum test on the performance of POA against each of the competitor algorithms in dealing with the CEC 2017 test suite are reported in Table 6. Based on the results of the statistical analysis, in cases where the *p*-value is calculated to be less than 0.05, POA has a significant statistical superiority over the competitor algorithm. Therefore, POA has a significant statistical superiority in handling the CEC 2017 test suite for problem dimensions equal to 10, 30, 50, and 100 compared to all twelve competitor algorithms.

## 5. POA for Real-World Applications

In this section, the performance of the proposed POA approach in handling optimization tasks in real-world applications is evaluated. For this purpose, twenty-two constrained optimization problems from the CEC 2011 test suite and four engineering design problems are selected. The titles of these real-world applications are parameter estimation for frequency-modulated (FM) sound waves, Lennard-Jones potential problem, the bifunctional catalyst blend optimal control problem, optimal control of a non-linear stirred tank reactor, tersoff potential for model Si (B), tersoff potential for model Si (C), spread spectrum radar polly phase code design, transmission network expansion planning (TNEP) problem, large scale transmission pricing problem, circular antenna array design problem, the ELD problems (consisting of DED instance 1, DED instance 2, ELD instance 1, ELD instance 2, ELD instance 3, ELD instance 4, ELD instance 5, hydrothermal scheduling instance 1, hydrothermal scheduling instance 2, hydrothermal scheduling instance 3), messenger: spacecraft trajectory optimization problem, and cassini 2: spacecraft trajectory optimization problem. From this set, the C11-F3 function has been removed in the simulation studies from the CEC 2011 test suite, as well as four engineering design problems of pressure vessel design, speed reducer design, welded beam design, and tension/compression spring design.

### 5.1. Evaluation of CEC 2011 Test Suite

In this subsection, the performance of POA and competitor algorithms in handling the CEC 2011 test suite is evaluated. The CEC 2011 test suite contains twenty-two constrained optimization problems from real-world applications (Appendix A). A full description, details, and information about the CEC 2011 test suite are available in [69].

**Figure 7:**
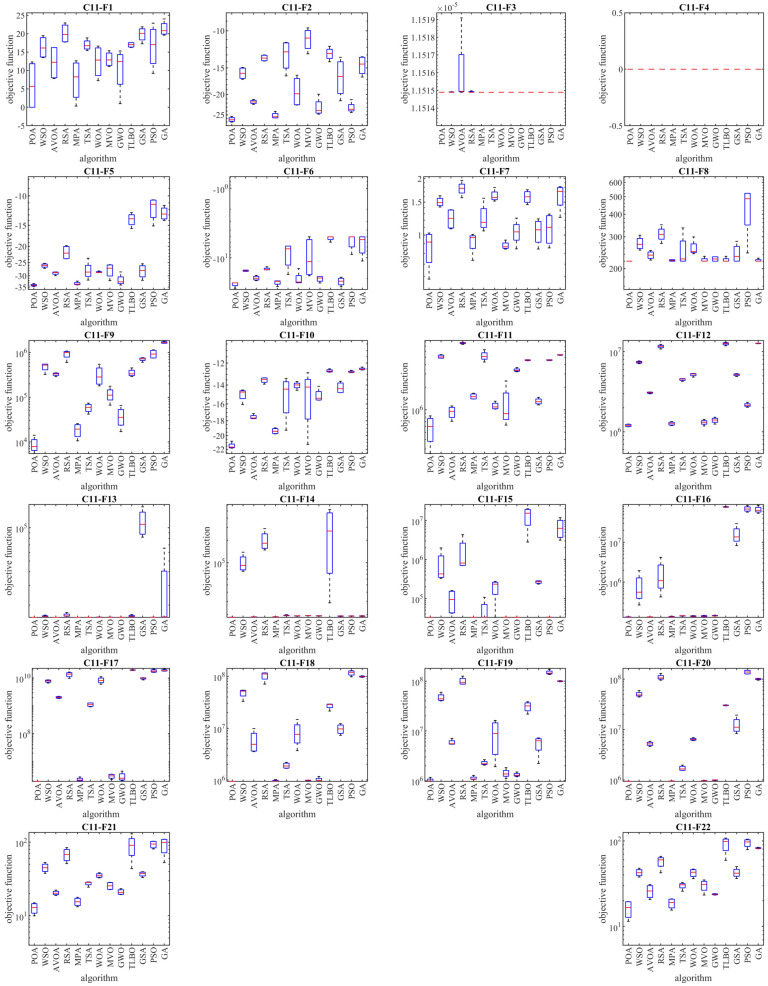
Boxplot diagrams of POA and competitor algorithms’ performances on CEC 2011 test suite.

The optimization results of the CEC 2011 test suite using POA and competitor algorithms are reported in Table 7. The boxplot diagrams obtained from the performance of metaheuristic algorithms are plotted in Figure 7. The optimization results show that POA, with its high ability in exploration, exploitation, and balancing them, has been able to achieve effective results for optimization problems and be the first best optimizer for problems C11-F1 to C11-F22. What can be concluded from the simulation results is that POA has provided superior performance by providing better results in most of the optimization problems and getting the rank of the first best optimizer to deal with the CEC 2011 test suite compared to competitor algorithms. In addition, the statistical results obtained from the Wilcoxon rank sum test confirm that POA has significant statistical superiority compared to competitor algorithms.

### 5.2. Pressure Vessel Design Problem

**Figure 8:**
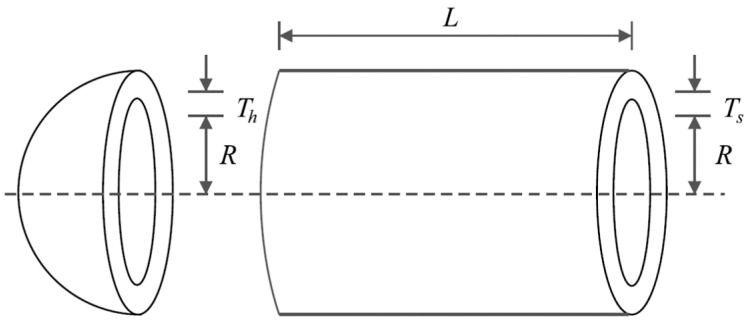
Schematic of pressure vessel design.

Pressure vessel design is a real-world application with the issue of minimizing construction cost. The schematic of this design is shown in Figure 8 and its mathematical model is given below [70]:

*Consider*: $X=\left[x_{1},x_{2},x_{3},x_{4}\right]=\left[T_{s},T_{h},R,L\right]$.

*Minimize*: $f\left(x\right)=0.6224x_{1}x_{3}x_{4}+1.778x_{2}x_{3}^{2}+3.1661x_{1}^{2}x_{4}+19.84x_{1}^{2}x_{3}.$

*Subject to*:

$$g_{1}\left(x\right)=-x_{1}+0.0193x_{3}\;\leq \;0,\;\;g_{2}\left(x\right)=-x_{2}+0.00954x_{3}\leq \;0,$$

$$g_{3}\left(x\right)=-\pi x_{3}^{2}x_{4}-\frac{4}{3}\pi x_{3}^{3}+1296000\leq \;0,\;\;g_{4}\left(x\right)=x_{4}-240\;\leq \;0.$$

With

$$0\leq x_{1},x_{2}\leq 100\;{and\;10\leq x}_{3},x_{4}\leq 200.$$

**Figure 9:**
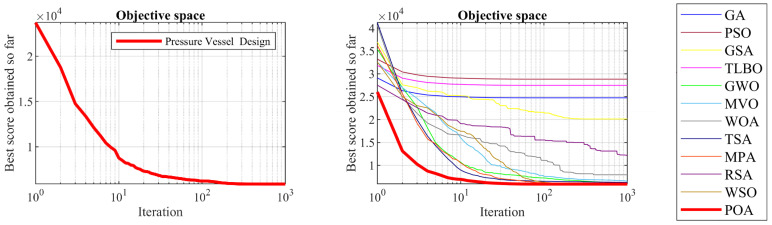
POA’s performance convergence curve on pressure vessel design.

The results of the implementation of POA and competitor algorithms on the pressure vessel design problem are reported in Table 8 and Table 9. The convergence curve of POA while achieving the optimal design is plotted in Figure 9. Based on the obtained results, POA has provided the optimal design with the values of the design variables equal to 0.7780271, 0.3845792, 40.312284, and 200 and the value of the objective function equal to 5882.8955. Simulation results show that POA has provided superior performance by achieving better results to optimize the pressure vessel design problem compared to competitor algorithms.

### 5.3. Speed Reducer Design Problem

**Figure 10:**
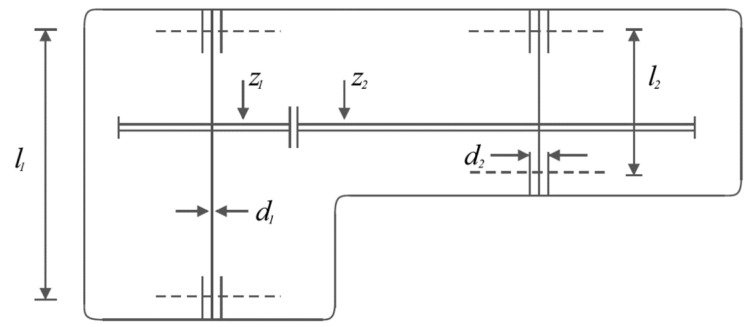
Schematic of speed reducer design.

Speed reducer design is a real-world application with the issue of minimizing the weight of the speed reducer. Schematic of this design is shown in Figure 10 and its mathematical model is given below [71,72]:

*Consider:*$X=\left[x_{1,}x_{2},x_{3},x_{4},x_{5}{,x}_{6},x_{7}\right]=\left[b,m,p,l_{1},l_{2},d_{1},d_{2}\right]$.

*Minimize:*$f\left(x\right)=0.7854x_{1}x_{2}^{2}\left(3.3333x_{3}^{2}+14.9334x_{3}-43.0934\right)-1.508x_{1}\left(x_{6}^{2}+x_{7}^{2}\right)+7.4777\left(x_{6}^{3}+x_{7}^{3}\right)+0.7854(x_{4}x_{6}^{2}+x_{5}x_{7}^{2})$.

*Subject to:*

$$g_{1}\left(x\right)=\frac{27}{x_{1}x_{2}^{2}x_{3}}-1\leq 0,g_{2}\left(x\right)=\frac{397.5}{x_{1}x_{2}^{2}x_{3}}-1\leq 0,$$

$$g_{3}\left(x\right)=\frac{1.93x_{4}^{3}}{x_{2}x_{3}x_{6}^{4}}-1\leq 0,g_{4}\left(x\right)=\frac{1.93x_{5}^{3}}{x_{2}x_{3}x_{7}^{4}}-1\leq 0,$$

$$g_{5}\left(x\right)=\frac{1}{110x_{6}^{3}}\sqrt{{\left(\frac{745x_{4}}{x_{2}x_{3}}\right)}^{2}+16.9\times 10^{6}}-1\leq 0,$$

$$g_{6}(x)=\frac{1}{85x_{7}^{3}}\sqrt{{\left(\frac{745x_{5}}{x_{2}x_{3}}\right)}^{2}+157.5\times 10^{6}}-1\leq 0,$$

$$g_{7}\left(x\right)=\frac{x_{2}x_{3}}{40}-1\leq 0,g_{8}\left(x\right)=\frac{{5x}_{2}}{x_{1}}-1\leq 0,$$

$$g_{9}\left(x\right)=\frac{x_{1}}{12x_{2}}-1\leq 0,g_{10}\left(x\right)=\frac{{1.5x}_{6}+1.9}{x_{4}}-1\leq 0,$$

$$g_{11}\left(x\right)=\frac{{1.1x}_{7}+1.9}{x_{5}}-1\leq 0.$$

With

$$2.6\leq x_{1}\leq 3.6,0.7\leq x_{2}\leq 0.8,17\leq x_{3}\leq 28,7.3\leq x_{4}\leq 8.3,7.8\leq x_{5}\;\leq 8.3,2.9\leq x_{6}\leq 3.9,\;and\;5\leq x_{7}\leq 5.5.$$

**Figure 11:**
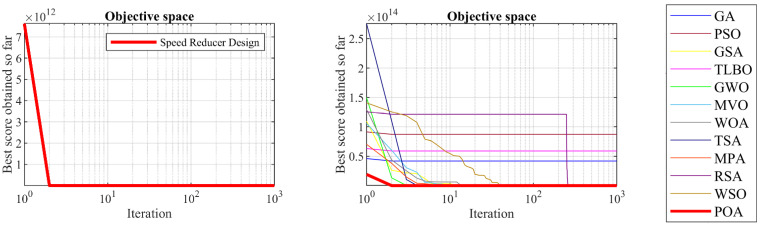
POA’s performance convergence curve on speed reducer design.

The results of employing POA and competitor algorithms on the speed reducer design problem are presented in Table 10 and Table 11. The convergence curve of POA while achieving the optimal design for the speed reducer problem is drawn in Figure 11. Based on the obtained results, POA has provided the optimal design with the values of the design variables equal to 3.5, 0.7, 17, 7.3, 7.8, 3.3502147, and 5.2866832 and the value of the objective function equal to 2996.3482. What is evident from the analysis of simulation results is that POA has provided superior performance by achieving better results to solve the speed reducer design problem compared to competitor algorithms.

### 5.4. Welded Beam Design

**Figure 12:**
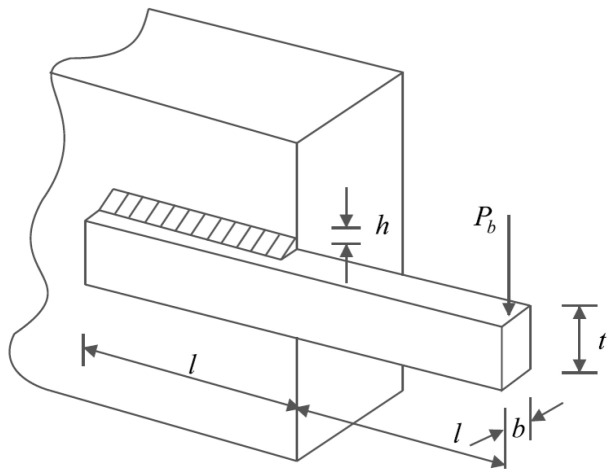
Schematic of welded beam design.

Welded beam design is a real-world application with the issue of minimizing the fabrication cost of the welded beam. The schematic of this design is shown in Figure 12 and its mathematical model is given below [24]:

*Consider*: $X=\left[x_{1},x_{2},x_{3},x_{4}\right]=\left[h,l,t,b\right]$.

*Minimize*: $f(x)=1.10471x_{1}^{2}x_{2}+0.04811x_{3}x_{4}(14.0+x_{2})$.

*Subject to*:

$$g_{1}\left(x\right)=\tau \left(x\right)-13600\;\leq \;0,\;\;g_{2}\left(x\right)=\sigma \left(x\right)-30000\;\leq \;0,$$

$$g_{3}\left(x\right)=x_{1}-x_{4}\leq \;0,\;\;g_{4}(x)=0.10471x_{1}^{2}+0.04811x_{3}x_{4}\;(14+x_{2})-5.0\;\leq \;0,$$

$$g_{5}\left(x\right)=0.125-x_{1}\leq \;0,\;\;g_{6}\left(x\right)=\delta \;\left(x\right)-0.25\;\leq \;0,$$

$$g_{7}\left(x\right)=6000-p_{c}\;\left(x\right)\leq \;0.$$

*where*

$$\tau \left(x\right)=\sqrt{{\left(\tau^{\prime }\right)}^{2}+\left(2\tau \tau^{\prime }\right)\frac{x_{2}}{2R}+{\left(\tau \prime \prime \right)}^{2}\;},\;\;\tau^{\prime }=\frac{6000}{\sqrt{2}x_{1}x_{2}},\;\;\tau \prime \prime =\frac{MR}{J},$$

$$M=6000\left(14+\frac{x_{2}}{2}\right),\;\;R=\sqrt{\frac{x_{2}^{2}}{4}+{\left(\frac{x_{1}+x_{3}}{2}\right)}^{2}},$$

$$J=2\left\{x_{1}x_{2}\sqrt{2}\left[\frac{x_{2}^{2}}{12}+{\left(\frac{x_{1}+x_{3}}{2}\right)}^{2}\right]\right\}\;,\;\;\;\sigma \left(x\right)=\frac{504000}{x_{4}x_{3}^{2}}\;,$$

$$\delta \;\left(x\right)=\frac{65856000}{\left(30\cdot 10^{6}\right)x_{4}x_{3}^{3}}\;,\;\;p_{c}\;\left(x\right)=\frac{4.013\left(30\cdot 10^{6}\right)\sqrt{\frac{x_{3}^{2}x_{4}^{6}}{36}}}{196}\left(1-\frac{x_{3}}{28}\sqrt{\frac{30\cdot 10^{6}}{4(12\cdot 10^{6})}}\right)\;.$$

With

$$0.1\leq x_{1},\;x_{4}\leq 2\;\;\;and\;0.1\leq x_{2},\;x_{3}\leq 10.$$

**Figure 13:**
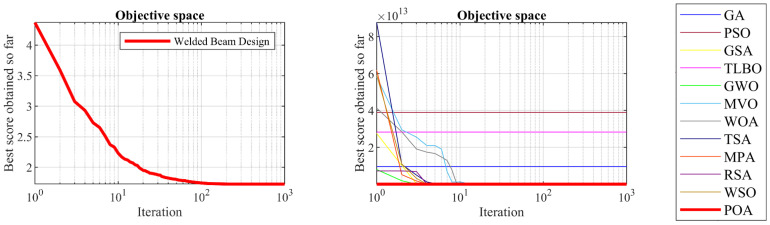
POA’s performance convergence curve on welded beam design.

The results of dealing with the welded beam design problem using POA and competitor algorithms are reported in Table 12 and Table 13. The POA convergence curve while achieving the optimal design for the welded beam problem is plotted in Figure 13. Based on the obtained results, POA has provided the optimal design with the values of the design variables equal to 0.2057296, 3.4704887, 9.0366239, and 0.2057296 and the value of the objective function equal to 1.7246798. Analysis of the simulation results shows that POA provides superior performance for solving the welded beam design problem by achieving better results compared to competitor algorithms.

### 5.5. Tension/Compression Spring Design

**Figure 14:**
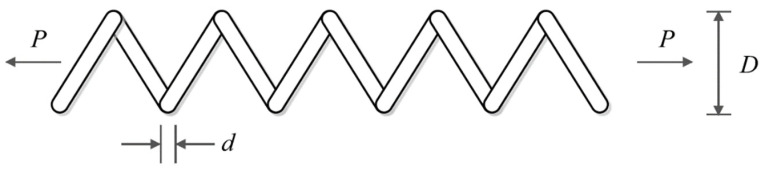
Schematic of tension/compression spring design.

Tension/compression spring design is a real-world application with the issue of minimizing construction cost. The schematic of this design is shown in Figure 14 and its mathematical model is given below [24]:

*Consider*: $X=\left[x_{1},x_{2},x_{3}\right]=\left[d,D,P\right].$

*Minimize*: $f\left(x\right)=\left(x_{3}+2\right)x_{2}x_{1}^{2}.$

*Subject to*:

$$g_{1}\left(x\right)=1-\frac{x_{2}^{3}x_{3}}{71785x_{1}^{4}}\;\leq \;0,\;\;g_{2}\left(x\right)=\frac{4x_{2}^{2}-x_{1}x_{2}}{12566(x_{2}x_{1}^{3})}+\frac{1}{5108x_{1}^{2}}-1\leq \;0,$$

$$g_{3}\left(x\right)=1-\frac{140.45x_{1}}{x_{2}^{2}x_{3}}\leq \;0,\;\;\;g_{4}\left(x\right)=\frac{x_{1}+x_{2}}{1.5}-1\;\leq \;0.$$

With

$$0.05\leq x_{1}\leq 2,\;{0.25\leq x}_{2}\leq 1.3\;\;\;\;and\;\;\;\;2\leq \;x_{3}\leq 15$$

**Figure 15:**
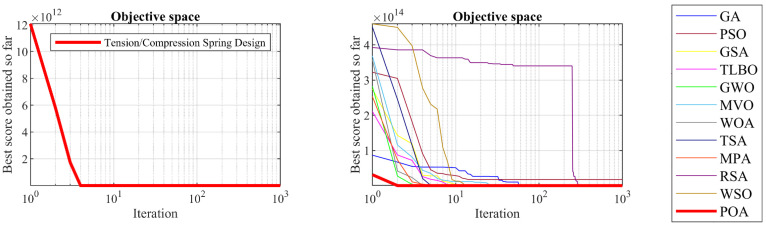
POA’s performance convergence curve on tension/compression spring.

The optimization results of the tension/compression spring design problem using POA and competitor algorithms are reported in Table 14 and Table 15. The convergence curve of POA while achieving the optimal design for the tension/compression spring problem is drawn in Figure 15. Based on the obtained results, POA has provided the optimal design with the values of the design variables equal to 0.0516891, 0.3567177, and 11.288966 and the value of the objective function equal to 0.0126019. What can be concluded from the simulation results is that POA provides superior performance by achieving better results in order to deal with the tension/compression spring design problem compared to competitor algorithms.

## 6. Conclusions and Future Works

A new bio-inspired metaheuristic algorithm, called the Pufferfish Optimization Algorithm (POA), which imitates the natural behavior between pufferfish and their predators in the sea, is introduced in this paper. The fundamental inspiration for POA is derived from the attacks of hungry predators on pufferfish and the defense mechanism of pufferfish against these attacks. The theory of POA is described and mathematically modeled in two phases, (i) exploration based on the simulation of the predator attack on pufferfish and (ii) exploitation based on the simulation of the escape of the predator from the spiny spherical pufferfish. The performance of POA is evaluated in handling the CEC 2017 test suite for problem dimensions equal to 10, 30, 50, and 100. The optimization results show that POA has a high ability in exploration, exploitation, and the balance between them during the search process to provide effective solutions. To measure the ability of POA in optimization, the obtained results are compared with the performance of twelve well-known metaheuristic algorithms. Simulation results show that POA provides superior performance compared to competitor algorithms by achieving better results for most of the benchmark functions. The use of the Wilcoxon rank sum test statistical analysis confirmed that this superiority of POA is also significant from a statistical point of view. In addition, the effectiveness of POA in handling real-world applications was challenged in handling twenty-two constrained optimization problems from the CEC 2011 test suite and four engineering design problems. The optimization results show that POA offers effective performance to handle optimization tasks in real-world applications.

Based on the simulation results, in handling the CEC 2017 test suite for the problem dimension equal to 10, the proposed POA approach had the best performance in 24/29 functions, i.e., 82.75%. For the problem dimension equal to 30, POA was successful in 27/29 functions, i.e., 93.10%. For the problem dimension equal to 50, POA performed best in 28/29 functions, i.e., 96.55%. For the problem dimension equal to 100, POA performed best in 29/29 functions, i.e., 100%. Also, the proposed POA approach in dealing with real-world applications consisting of the CEC 2011 test suite and four engineering design problems presented the best performance in 26/26 optimization problems, i.e., 100%.

The proposed POA approach has several advantages for global optimization problems. The first advantage of POA is that there is no control parameter in the design of this algorithm. Therefore, there is no need to control the parameters in any way. The second advantage of POA is its high effective efficiency in dealing with a variety of optimization problems in various sciences as well as complex high-dimensional problems. The third advantage of the proposed POA method is that it shows its great ability to balance exploration and exploitation in the search process, which allows it to have high-speed convergence to provide suitable values for the decision variables in optimization tasks, especially in complex problems. The fourth advantage of the proposed POA is its powerful performance in handling real-world optimization applications. However, there are several disadvantages and limitations regarding POA. The first one is that because POA is a stochastic approach, there is no guarantee to achieve the global optimum using the proposed POA approach. The second disadvantage of POA is that based on the NFL theorem, there is no assumption about the success or failure of its implementation on an optimization problem. The third disadvantage is that there is always the possibility that newer metaheuristic algorithms will be designed that perform better compared to POA.

The introduction of POA enables several research proposals for future work. The most special of these research proposals is the development of multi-objective and binary versions of the proposed POA approach. Also, the employment of POA to deal with optimization issues in different sciences and real-world applications is one of the other research proposals of this study for future work.

## Figures and Tables

**Table 1:** Control parameter values.

Algorithm	Parameter	Value
GA		
	Type	Real coded
	Selection	Roulette wheel (Proportionate)
	Crossover	Whole arithmetic (Probability = 0.8, $\alpha \in \left[-0.5,\;1.5\right]$)
	Mutation	Gaussian (Probability = 0.05)
PSO		
	Topology	Fully connected
	Cognitive and social constant	(*C*_1_, *C*_2_) =(2, 2)
	Inertia weight	Linear reduction from 0.9 to 0.1
	Velocity limit	10% of dimension range
GSA		
	Alpha, *G*_0_, *R_norm_*, *R_power_*	20, 100, 2, 1
TLBO		
	*T_F_*: teaching factor	*T_F_* = round $\left[\left(1+rand\right)\right]$
	random number	*rand* is a random number between $\left[0-1\right].$
GWO		
	Convergence parameter (*a*)	*a*: Linear reduction from 2 to 0.
MVO		
	Wormhole existence probability (WEP)	Min(WEP) = 0.2 and Max(WEP) = 1.
	Exploitation accuracy over the iterations (*p*)	p=6.
WOA		
	Convergence parameter (*a*)	*a*: Linear reduction from 2 to 0.
	*r* is a random vector in $\left[0-1\right].$	
	*l* is a random number in $\left[-1,1\right].$	
TSA		
	P_min_ and P_max_	1, 4
	*c1*, *c2*, *c3*	Random numbers lie in the range of $\left[0-1\right].$
MPA		
	Constant number	*P* = 0.5
	Random vector	*R* is a vector of uniform random numbers in $\left[0,\;1\right].$
	Fish Aggregating Devices (*FADs*)	*FADs* = 0.2
	Binary vector	*U* = 0 or 1
RSA		
	Sensitive parameter	β=0.01
	Sensitive parameter	α=0.1
	Evolutionary Sense (ES)	ES: randomly decreasing values between 2 and −2
AVOA		
	L_1_, L_2_	0.8, 0.2
	w	2.5
	P_1_, P_2_, P_3_	0.6, 0.4, 0.6
WSO		
	F_min_ and F_max_	0.07, 0.75
	*τ, a_o_, a*_1_ *, a*_2_	4.125, 6.25, 100, 0.0005

**Table 2:** Optimization results of CEC 2017 test suite (dimension = 10); background color has been used in order to make the table more reader-friendly and to separate the results of benchmark functions from each other; The best results are specified using bold.

		POA	WSO	AVOA	RSA	MPA	TSA	WOA	MVO	GWO	TLBO	GSA	PSO	GA
C17-F1	mean	**100**	4.55 × 10^9^	3225.251	8.52 × 10^9^	29,456,372	1.45 × 10^9^	5,384,532	6295.129	73,640,145	1.23 × 10^8^	639.77	2641.639	9,894,286
	best	**100**	3.82 × 10^9^	113.0384	7.37 × 10^9^	9369.202	3.11 × 10^8^	3,920,729	4010.164	23,221.73	54,735,477	100.0161	305.0863	5,123,646
	worst	**100**	5.71 × 10^9^	9961.716	1.02 × 10^10^	1.07 × 10^8^	3.17 × 10^9^	7,089,395	9268.077	2.68 × 10^8^	2.96 × 10^8^	1510.948	7789.605	14,204,094
	std	**0**	8.41 × 10^8^	4725.098	1.29 × 10^9^	53,343,077	1.31 × 10^9^	1,377,344	2529.889	1.33 × 10^8^	1.2 × 10^8^	626.9959	3559.58	3,898,254
	median	**100**	4.34 × 10^9^	1413.125	8.29 × 10^9^	5,399,185	1.17 × 10^9^	5,264,003	5951.137	13,496,678	70,182,972	474.0581	1235.933	10,124,702
	rank	**1**	12	4	13	8	11	6	5	9	10	2	3	7
C17-F3	mean	**300**	6378.9	301.5805	8102.009	1224.369	9399.415	1493.376	300.0456	2610.921	655.7709	8611.105	300	12,379.73
	best	**300**	3479.574	300	4391.427	710.0549	3610.069	566.4824	300.0106	1325.137	442.915	5437.139	300	3679.862
	worst	**300**	8532.601	303.3805	10,823.2	2165.316	13,268.46	2829.352	300.1038	4963.289	794.8168	11,686.32	300	19,538.86
	std	**0**	2278.712	1.88337	3020.559	689.6101	4212.539	1094.985	0.042055	1724.025	158.4443	2647.296	4.77 × 10^−14^	8508.885
	median	**1.310345**	10.96552	6.103448	12.06897	3.655172	9.862069	8.241379	4	6.482759	6.586207	8.206897	6.310345	6.793103
	rank	**1**	9	4	10	6	12	7	3	8	5	11	2	13
C17-F4	mean	**400**	832.9399	403.9689	1194.33	405.6187	547.3643	421.0073	402.7854	409.8048	407.6604	403.8033	416.9675	412.2951
	best	**400**	627.2234	401.0368	771.6337	402.0435	465.0221	405.381	401.3315	405.0867	407.0049	402.975	400.0883	409.7553
	worst	**400**	1019.23	405.4518	1608.364	409.5054	643.505	461.4439	404.0891	423.6902	408.0743	405.0755	458.7854	415.4025
	std	**0**	180.4109	2.136488	366.8366	3.780469	89.85733	27.77266	1.472703	9.507988	0.471	0.989405	28.92907	2.538467
	median	**400**	842.6533	404.6935	1198.66	405.4629	540.4651	408.6021	402.8604	405.2212	407.7813	403.5813	404.4981	412.0112
	rank	**1**	12	4	13	5	11	10	2	7	6	3	9	8
C17-F5	mean	**501.2464**	552.1378	537.357	561.6212	511.0763	554.4922	534.7626	520.197	511.1956	528.9305	545.6336	523.7417	523.836
	best	**500.9951**	539.7089	522.8013	549.3927	507.3653	536.6275	519.9448	508.7872	507.3485	524.2608	541.4915	509.5614	519.8255
	worst	**501.9917**	560.5654	553.1728	574.2389	515.3498	581.634	565.1408	532.364	517.3034	531.87	555.5081	543.8277	528.6581
	std	**0.510361**	9.580768	16.32801	14.21183	4.349562	20.50914	21.74587	10.11023	4.390641	3.433171	6.847497	16.20352	4.062694
	median	**500.9993**	554.1385	536.7271	561.4266	510.7951	549.8536	526.9824	519.8183	510.0652	529.7956	542.7673	520.7889	523.4302
	rank	**1**	11	9	13	2	12	8	4	3	7	10	5	6
C17-F6	mean	**600**	627.3309	614.6691	634.4767	601.0111	621.0302	619.6218	601.8208	600.9545	605.8124	614.5724	606.2928	608.69
	best	**600**	623.5055	613.8184	631.7558	600.6021	612.7676	606.3745	600.3998	600.5048	604.0305	602.47	601.1473	605.8484
	worst	**600**	631.2956	616.8301	638.0792	602.0311	634.2348	638.2827	603.6532	601.4559	608.5909	630.6117	616.3121	612.2851
	std	**0**	3.472219	1.484036	2.918649	0.700246	9.511675	13.80004	1.501618	0.404385	2.135493	13.37183	7.064953	2.930533
	median	**600**	627.2613	614.0139	634.0359	600.7057	618.5592	616.915	601.6152	600.9287	605.3141	612.604	603.856	608.3133
	rank	**1**	12	9	13	3	11	10	4	2	5	8	6	7
C17-F7	mean	**711.1267**	783.4441	757.2336	790.1017	722.5726	810.5305	754.2906	727.8586	723.7378	745.795	716.2033	729.4378	732.9366
	best	**710.6726**	769.04	738.836	778.8415	719.0263	776.5166	745.0046	716.3701	716.5132	741.9062	714.3682	723.4728	724.2721
	worst	**711.7995**	793.0312	780.7714	800.7603	726.2643	845.5942	779.3306	744.1291	738.5278	752.6074	719.2987	739.1632	736.7494
	std	**0.526035**	10.46384	19.76913	10.5544	3.115174	30.80953	17.19406	12.0106	10.37385	4.88509	2.205896	7.383528	6.02182
	median	**711.0174**	785.8527	754.6635	790.4025	722.4998	810.0057	746.4136	725.4676	719.955	744.3332	715.5731	727.5577	735.3625
	rank	**1**	11	10	12	3	13	9	5	4	8	2	6	7
C17-F8	mean	**801.4928**	840.4137	826.6076	845.7371	810.9673	841.1529	831.0484	810.2577	813.6633	832.19	817.0678	819.5292	814.4624
	best	**800.995**	835.9776	817.349	836.3057	807.6532	827.5049	815.903	806.4482	809.0741	826.2583	810.3401	813.4552	811.0084
	worst	**801.9912**	845.801	839.933	850.1049	812.8626	857.434	841.4662	814.2402	817.8153	838.8887	823.5791	825.0745	820.994
	std	**0.590448**	5.260983	9.771921	6.548268	2.434302	13.71341	11.28228	3.284991	3.76151	6.627541	5.787728	5.924469	4.572311
	median	**801.4926**	839.938	824.5741	848.2689	811.6767	839.8363	833.4122	810.1712	813.8819	831.8065	817.1759	819.7935	812.9235
	rank	**1**	11	8	13	3	12	9	2	4	10	6	7	5
C17-F9	mean	**900**	1342.665	1144.15	1380.729	904.4051	1307.355	1302.765	900.6792	910.1139	910.0229	**900**	903.5951	904.3318
	best	**900**	1213.579	945.5294	1299.548	900.2775	1127.127	1047.555	900.0009	900.4858	906.1292	**900**	900.7621	902.3713
	worst	**900**	1461.794	1545.028	1497.235	911.3074	1551.625	1540.964	902.6395	928.0779	916.9536	**900**	910.4403	907.6936
	std	**0**	110.3068	285.318	86.45428	5.099179	188.6618	213.3405	1.342755	13.29542	4.886051	**0**	4.746802	2.472394
	median	**900**	1347.644	1043.021	1363.067	903.0177	1275.335	1311.271	900.0381	905.9459	908.5044	**900**	901.589	903.6311
	rank	**1**	11	8	12	5	10	9	2	7	6	**1**	3	4
C17-F10	mean	**1006.179**	2094.711	1653.575	2325.702	1434.373	1867.549	1861.168	1656.066	1609.581	1984.581	2073.645	1794.689	1601.404
	best	**1000.284**	1844.829	1407.465	2183.265	1329.997	1635.581	1379.004	1382.572	1453.891	1657.353	1839.94	1472.001	1349.864
	worst	**1012.668**	2216.253	2188.593	2626.801	1496.489	2080.065	2300.463	2076.287	1832.601	2225.617	2161.619	2134.667	1932.365
	std	**6.836865**	177.0655	377.3195	212.4787	80.45788	240.0084	458.2015	344.9953	165.4887	248.0571	160.3531	279.4831	256.8364
	median	**1005.882**	2158.882	1509.12	2246.371	1455.502	1877.275	1882.603	1582.703	1575.916	2027.676	2146.51	1786.043	1561.693
	rank	**1**	12	5	13	2	9	8	6	4	10	11	7	3
C17-F11	mean	**1100**	3052.848	1140.664	3518.066	1122.675	4754.993	1142.722	1123.061	1146.339	1142.683	1132.859	1136.493	2175.454
	best	**1100**	2007.707	1114.294	1400.652	1111.067	4630.721	1110.865	1104.65	1118.127	1131.718	1116.47	1127.039	1112.613
	worst	**1100**	4072.84	1185.335	5609.441	1149.281	4823.178	1161.289	1141.008	1207.627	1160.621	1157.526	1154.514	5190.671
	std	**0**	952.1951	32.11656	1942.817	18.53391	87.85611	23.91512	18.65818	42.85208	12.81477	17.99745	12.70843	2065.104
	median	**1100**	3065.423	1131.514	3531.087	1115.176	4783.037	1149.367	1123.294	1129.801	1139.198	1128.719	1132.21	1199.267
	rank	**1**	11	6	12	2	13	8	3	9	7	4	5	10
C17-F12	mean	**1352.959**	2.97 × 10^8^	925,391.7	5.93 × 10^8^	477,319.8	874,188.8	1,979,065	865,306	1,189,969	4,247,558	857,970	7016.058	508,801.4
	best	**1318.646**	66,857,398	299,477.1	1.32 × 10^8^	16,909.18	453,430	144,583.9	7633.405	38,406.71	1,136,852	399,108.9	2327.135	147,522.3
	worst	**1438.176**	5.19 × 10^8^	1,678,010	1.04 × 10^9^	746,882.4	1,073,191	3,283,074	2,717,573	1,862,542	7,519,307	1,450,813	11,928.71	897,999.2
	std	**58.85078**	2.35 × 10^8^	662,257.5	4.7 × 10^8^	330,296.3	300,169.8	1,498,477	1,285,727	825,776.5	3,472,325	457,249.7	4491.941	316,505.5
	median	**1327.506**	3.01 × 10^8^	862,039.8	6.02 × 10^8^	572,743.7	985,067.2	2,244,302	368,008.7	1,429,464	4,167,037	790,979.2	6904.195	494,842
	rank	**1**	12	8	13	3	7	10	6	9	11	5	2	4
C17-F13	mean	**1305.324**	14,453,034	15,617.23	28,897,755	4775.974	10,914.64	6578.518	5863.709	8864.918	14,267.13	8673.501	5773.513	45,976.95
	best	**1303.114**	1,205,133	2496.902	2,399,357	3335.283	6585.883	2965.887	1373.109	5677.498	13,483.27	4451.162	2207.669	7389.402
	worst	**1308.508**	47,973,526	26,610.71	95,934,824	5794.402	17,170.59	12,945.79	10,613.54	12,301.39	16,181.02	12,131.5	14,257.57	151,548.5
	std	**2.334346**	23,002,094	12,803.89	46,001,701	1204.556	4692.115	4672.716	4916.238	2788.105	1323.027	3334.195	5873.675	72,330.17
	median	**1304.837**	4,316,738	16,680.66	8,628,419	4987.106	9951.053	5201.196	5734.095	8740.391	13,702.11	9055.67	3314.406	12,484.95
	rank	**1**	12	10	13	2	8	5	4	7	9	6	3	11
C17-F14	mean	**1400.746**	3425.125	1923.395	4721.354	1854.633	3071.795	1500.297	1544.788	2196.524	1560.721	4905.279	2742.875	11,128.99
	best	**1400**	2876.326	1634.989	4160.638	1429.482	1474.157	1469.026	1419.609	1452.607	1497.896	4094.539	1427.371	3357.938
	worst	**1400.995**	4457.139	2601.909	6025.968	2665.305	4920.32	1533.639	1899.666	4398.761	1586.247	6578.375	5981.161	21,956.75
	std	**0.510957**	743.7893	468.0622	899.962	595.2986	1882.86	33.93495	243.0516	1507.96	43.22455	1195.352	2235.262	8092.2
	median	**1400.995**	3183.518	1728.342	4349.405	1661.872	2946.352	1499.262	1429.938	1467.363	1579.371	4474.102	1781.483	9600.644
	rank	**1**	10	6	11	5	9	2	3	7	4	12	8	13
C17-F15	mean	**1500.331**	8823.289	4695.909	11,912.34	3583.354	6130.176	5470.285	1535.266	5130.307	1676.091	20,332.03	7808.495	4066.484
	best	**1500.001**	2966.931	1981.77	2538.513	2950.103	2189.717	1932.997	1521.862	3241.986	1570.783	9684.199	2654.209	1828.644
	worst	**1500.5**	14,888.97	10,862.78	25,783.44	4354.214	10,794.45	11,552.87	1545.424	6043.794	1751.572	30,396.01	12,686.41	6981.342
	std	**0.241803**	5273.186	4255.399	10,424.41	598.2652	3797.989	4306.832	10.57788	1322.269	91.12372	10,162.76	4306.45	2631.154
	median	**1500.413**	8718.629	2969.542	9663.698	3514.55	5768.27	4197.636	1536.889	5617.724	1691.004	20,623.96	7946.681	3727.975
	rank	**1**	11	6	12	4	9	8	2	7	3	13	10	5
C17-F16	mean	**1600.76**	1939.004	1776.431	1950.451	1670.982	1976.428	1894.978	1782.044	1708.223	1664.815	1998.193	1872.397	1770.358
	best	**1600.356**	1880.829	1635.665	1784.641	1635.301	1820.937	1738.957	1706.622	1613.492	1642.982	1892.192	1787.207	1699.991
	worst	**1601.12**	2038.394	1874.634	2180.76	1696.723	2131.631	2002.906	1833.931	1789.792	1710.474	2162.104	2006.809	1796.503
	std	**0.32447**	72.16946	103.3883	171.8207	27.14079	144.7973	128.8395	55.29935	74.70755	32.32517	126.0765	104.4724	48.224
	median	**1600.781**	1918.398	1797.713	1918.201	1675.953	1976.572	1919.025	1793.812	1714.804	1652.902	1969.237	1847.786	1792.468
	rank	**1**	10	6	11	3	12	9	7	4	2	13	8	5
C17-F17	mean	**1700.099**	1802.856	1742.924	1799.64	1730.095	1786.022	1819.437	1820.178	1757.693	1749.156	1823.538	1744.089	1747.136
	best	**1700.02**	1788.431	1729.01	1785.395	1718.446	1773.277	1761.955	1766.126	1720.64	1740.612	1740.352	1738.531	1744.49
	worst	**1700.332**	1808.997	1779.963	1807.391	1763.055	1795.177	1859.36	1910.802	1844.423	1757.517	1929.847	1749.707	1749.175
	std	**0.159367**	9.935453	25.42708	10.05161	22.58051	9.661591	43.46574	70.36918	59.68794	8.584843	99.23511	4.911284	2.168843
	median	**1700.022**	1806.997	1731.362	1802.888	1719.44	1787.818	1828.216	1801.892	1732.855	1749.248	1811.976	1744.06	1747.44
	rank	**1**	10	3	9	2	8	11	12	7	6	13	4	5
C17-F18	mean	**1805.36**	2,399,086	10,241.21	4,782,098	9564.09	10,411.16	19,850.53	17,869.42	16,995.77	25,051.26	8441.913	18,649.31	11,044.94
	best	**1800.003**	123,923.2	4355.372	237,008.5	3779.841	6555.448	5705.371	7595.718	5596.977	20,423.43	5658.557	2707.215	3173.378
	worst	**1820.451**	6,951,476	13,378.89	13,881,607	14,152.9	13,959.13	31,012.63	28,581.17	28,478.96	31,262.26	10,239.97	34,482.83	15,801.01
	std	**10.33599**	3,247,833	4155.94	6,493,306	4845.014	3162.788	12,525.29	10,147.75	11,917.16	5120.503	2008.171	16,847.52	5665.568
	median	**1800.492**	1,260,473	11,615.29	2,504,888	10,161.81	10,565.02	21,342.07	17,650.4	16,953.58	24,259.68	8934.562	18,703.59	12,602.68
	rank	**1**	12	4	13	3	5	10	8	7	11	2	9	6
C17-F19	mean	**1900.445**	325,504.3	5935.585	591,041.3	5003.989	105,644.8	29,515.62	1912.455	4823.948	4247.239	34,230.4	21,241.26	5494.354
	best	**1900.039**	21,793.92	2132.331	38,754.53	2250.713	1941.321	6733.843	1907.913	1937.739	2020.488	9623.886	2508.153	2162.926
	worst	**1901.559**	685,556.3	11,420.32	1,269,320	8208.202	210,717.9	53,779.76	1920.425	11,894.35	10,787.05	49512	64,828.83	8599.616
	std	**0.764786**	298,070.4	4638.779	570,173.4	3118.72	122,971.7	19,836.71	6.083054	4892.154	4478.154	18,348.08	30,181.34	2727.618
	median	**1900.09**	297,333.5	5094.842	528,045.5	4778.521	104,959.9	28,774.43	1910.741	2731.853	2090.709	38,892.85	8814.03	5607.437
	rank	**1**	12	7	13	5	11	9	2	4	3	10	8	6
C17-F20	mean	**2000.312**	2180.452	2143.185	2187.214	2077.529	2174.084	2173.426	2117.168	2142.643	2060.475	2212.957	2141.857	2042.189
	best	**2000.312**	2137.814	2026.344	2138.12	2061.102	2089.596	2082.569	2039.442	2109.914	2051.221	2157.634	2121.612	2030.104
	worst	**2000.312**	2236.68	2247.197	2233.726	2103.132	2269.357	2241.644	2207.7	2206.499	2069.219	2291.089	2168.583	2048.706
	std	**0**	42.30789	102.0477	48.32448	18.49766	78.21301	78.10245	70.95967	44.71939	7.750585	66.68667	23.97825	8.809191
	median	**2000.312**	2173.657	2149.599	2188.505	2072.941	2168.692	2184.746	2110.765	2127.08	2060.729	2201.552	2138.617	2044.974
	rank	**1**	11	8	12	4	10	9	5	7	3	13	6	2
C17-F21	mean	**2200**	2276.888	2211.596	2256.371	2248.035	2305.12	2292.26	2244.631	2295.146	2283.72	2341.352	2299.769	2282.443
	best	**2200**	2238.172	2203.467	2220.119	2245.945	2217.83	2215.447	2200.006	2291.612	2203.123	2326.674	2292.999	2222.304
	worst	**2200**	2301.147	2232.763	2276.984	2250.166	2344.556	2329.374	2290.374	2299.319	2316.211	2355.904	2306.111	2311.539
	std	**0**	29.46026	14.53934	25.83102	1.834601	60.80115	53.26074	52.93258	3.255673	55.58281	12.54634	6.624074	41.70537
	median	**2200**	2284.115	2205.076	2264.191	2248.015	2329.046	2312.11	2244.072	2294.826	2307.773	2341.415	2299.982	2297.965
	rank	**1**	6	2	5	4	12	9	3	10	8	13	11	7
C17-F22	mean	2300.073	2634.35	2307.561	2817.565	2304.217	2647.819	2320.013	**2288.058**	2307.239	2316.461	2300.016	2311.164	2315.079
	best	2300	2534.378	2303.667	2642.042	2300.793	2425.338	2316.081	**2240.701**	2301.065	2311.233	2300	2300.536	2312.63
	worst	2300.29	2733.762	2309.368	2946.395	2307.868	2822.497	2326.426	2304.494	2318.833	2326.313	**2300.063**	2338.212	2318.869
	std	0.149013	90.41136	2.697547	131.6308	3.057073	182.0508	4.737604	32.43726	8.39374	7.097258	**0.032379**	18.56028	2.740047
	median	**2300**	2634.631	2308.604	2840.912	2304.104	2671.721	2318.773	2303.519	2304.53	2314.148	2300	2302.954	2314.408
	rank	3	11	6	13	4	12	10	**1**	5	9	2	7	8
C17-F23	mean	**2600.919**	2675.602	2635.603	2684.856	2612.206	2704.076	2641.197	2617.205	2611.715	2636.003	2761.645	2637.469	2647.453
	best	**2600.003**	2646.262	2626.198	2660.766	2610.465	2629.043	2626.035	2606.13	2607.025	2626.737	2706.83	2631.721	2630.546
	worst	**2602.87**	2692.243	2650.446	2718.92	2614.369	2741.335	2658.505	2626.838	2617.257	2644.148	2878.017	2647.364	2654.735
	std	**1.356104**	22.27213	11.80346	27.9914	1.988046	52.16587	17.88037	9.21113	5.534287	7.906614	82.549	7.351876	11.7669
	median	**2600.403**	2681.952	2632.883	2679.868	2611.995	2722.963	2640.125	2617.926	2611.288	2636.562	2730.867	2635.396	2652.266
	rank	**1**	10	5	11	3	12	8	4	2	6	13	7	9
C17-F24	mean	**2630.488**	2762.071	2745.924	2815.196	2630.627	2662.311	2740.035	2674.963	2730.073	2736.013	2728.969	2744.203	2708.47
	best	2516.677	2708.447	2704.758	2780.272	2600.833	2541.706	2699.899	**2503.766**	2691.635	2707.8	2509.432	2723.673	2568.696
	worst	2732.32	2835.794	2776.692	2882.878	**2652.69**	2798.978	2781.865	2754.804	2755.634	2760.795	2871.458	2777.392	2798.205
	std	119.6573	55.18024	30.9873	48.1502	**25.83034**	134.8991	37.08252	119.6302	28.56004	28.06907	159.5899	26.51961	100.7278
	median	2636.477	2752.021	2751.124	2798.817	**2634.491**	2654.28	2739.188	2720.64	2736.512	2737.728	2767.493	2737.874	2733.49
	rank	**1**	12	11	13	2	3	9	4	7	8	6	10	5
C17-F25	mean	2932.639	3104.863	2916.531	3222.13	2920.238	3101.7	**2911.526**	2923.775	2937.735	2933.388	2923.919	2924.813	2949.13
	best	2898.047	3047.474	2898.929	3166.094	2913.328	2911.802	**2793.487**	2907.692	2925.184	2913.237	2909.09	2898.569	2931.825
	worst	2945.793	3232.527	2948.134	3280.582	**2926.531**	3537.034	2954.542	2943.657	2945.485	2950.891	2943.393	2946.433	2959.685
	std	23.71545	89.3546	22.24281	48.57388	**5.736208**	301.5004	80.86904	19.04683	9.501574	19.65598	17.75496	25.26368	12.48227
	median	2943.359	3069.725	**2909.53**	3220.923	2920.547	2978.982	2949.038	2921.876	2940.134	2934.712	2921.596	2927.126	2952.505
	rank	7	12	2	13	3	11	**1**	4	9	8	5	6	10
C17-F26	mean	2900	3453.143	2967.207	3620.709	2993.976	3506.647	3138.198	2900.124	3207.466	3158.072	3709.372	2903.417	**2897.657**
	best	2900	3178.785	2821.729	3348.132	2893.364	3105.541	2922.904	2900.095	2958.264	2910.145	2821.729	2821.729	**2737.911**
	worst	**2900**	3636.374	3116.144	3904.391	3231.247	4052.733	3484.204	2900.163	3747.685	3721.239	4119.597	2991.938	3076.415
	std	**3.81 × 10^−13^**	208.2441	172.5583	246.3436	163.2219	475.7115	252.054	0.030928	373.311	388.1521	617.6728	71.48489	176.0918
	median	2900	3498.708	2965.477	3615.157	2925.647	3434.157	3072.842	2900.12	3061.957	3000.451	3948.081	2900	**2888.152**
	rank	2	10	5	12	6	11	7	3	9	8	13	4	**1**
C17-F27	mean	**3089.518**	3190.876	3115.176	3208.701	3102.289	3165.276	3178.234	3091.294	3111.9	3111.042	3204.412	3128.703	3148.844
	best	**3089.518**	3150.567	3094.39	3121.245	3091.812	3100.385	3164.856	3089.68	3093.658	3094.456	3194.176	3095.896	3114.621
	worst	**3089.518**	3259.329	3166.45	3370.363	3126.797	3200.841	3188.133	3094.102	3162.963	3158.321	3222.553	3168.53	3198.444
	std	**2.7 × 10^−13^**	48.5967	35.21142	113.3577	16.90366	46.74527	9.978096	2.136325	34.99952	32.38107	12.96996	31.37261	36.39941
	median	**3089.518**	3176.804	3099.932	3171.599	3095.273	3179.939	3179.972	3090.697	3095.489	3095.696	3200.46	3125.193	3141.156
	rank	**1**	11	6	13	3	9	10	2	5	4	12	7	8
C17-F28	mean	**3100**	3514.354	3214.418	3670.974	3199.652	3508.782	3257.035	3216.62	3305.907	3289.216	3394.852	3272.889	3223.029
	best	**3100**	3476.288	3100	3601.782	3156.265	3362.699	3144.295	3100.104	3179.602	3195.809	3383.704	3164.824	3137.728
	worst	**3100**	3541.169	3344.066	3720.92	3220.577	3684.645	3344.495	3344.066	3362.306	3344.269	3410.343	3344.246	3447.511
	std	**0**	29.78092	110.8815	56.79583	30.57208	171.5013	105.6768	138.4256	87.16382	72.781	12.67838	83.55076	154.2956
	median	**3100**	3519.979	3206.803	3680.597	3210.882	3493.892	3269.675	3211.154	3340.86	3308.394	3392.68	3291.243	3153.438
	rank	**1**	12	3	13	2	11	6	4	9	8	10	7	5
C17-F29	mean	**3132.241**	3306.737	3260.455	3336.776	3191.911	3219.805	3314.628	3191.559	3244.171	3199.94	3312.094	3244.905	3220.644
	best	**3130.076**	3288.739	3197.72	3276.369	3160.311	3161.023	3219.005	3140.557	3181.05	3160.038	3218.075	3161.935	3179.846
	worst	**3134.841**	3320.542	3328.725	3392.986	3227.229	3278.448	3438.587	3261.751	3340.734	3219.468	3555.08	3314.906	3261.648
	std	**2.549599**	13.65937	69.3717	61.79221	30.11811	49.33958	94.5213	52.61156	78.09502	28.5812	167.0404	71.15231	35.44834
	median	**3132.023**	3308.834	3257.687	3338.874	3190.052	3219.874	3300.46	3181.964	3227.45	3210.127	3237.611	3251.389	3220.542
	rank	**1**	10	9	13	3	5	12	2	7	4	11	8	6
C17-F30	mean	**3418.734**	1,893,250	247,550.8	3,081,076	348,132.8	515,542.3	832,010.7	254,381.3	784,805.7	51,366.55	656,487.9	325,092.3	1,280,488
	best	**3394.682**	1,395,567	88,283.28	694,121.4	13,900.54	94,669.51	4300.199	6784.595	28,699.43	25,097.75	504,846.8	5916.503	441,195.3
	worst	**3442.907**	2,702,421	644,006.3	4,866,186	513,544.8	1,089,459	3,139,392	968,316.2	1,135,568	85,836.23	838,197.2	644,037	2,916,441
	std	**28.52304**	582,331.1	272,203.3	1,794,176	233,063.8	434,164.2	1,581,899	489,011.3	534,148.8	30,469.33	142,278.9	377,756.8	1,198,358
	median	**3418.673**	1,737,505	128,956.8	3,381,998	432,543	439,020.4	92,175.23	21,212.13	987,477.9	47,266.11	641,453.7	325,207.8	882,158.5
	rank	**1**	12	3	13	6	7	10	4	9	2	8	5	11
Sum rank		**38**	318	177	350	106	286	239	116	188	191	238	183	197
Mean rank		**1.310345**	10.96552	6.103448	12.06897	3.655172	9.862069	8.241379	4	6.482759	6.586207	8.206897	6.310345	6.793103
Total rank		**1**	12	4	13	2	11	10	3	6	7	9	5	8

**Table 3:** Optimization results of CEC 2017 test suite (dimension = 30); background color has been used in order to make the table more reader-friendly and to separate the results of benchmark functions from each other; The best results are specified using bold.

		POA	WSO	AVOA	RSA	MPA	TSA	WOA	MVO	GWO	TLBO	GSA	PSO	GA
C17-F1	mean	**100**	2.19 × 10^10^	2613.281	3.43 × 10^10^	22,347.55	1.49 × 10^10^	1.42 × 10^9^	448,448	1.39 × 10^9^	5.15 × 10^9^	8,761,210	1.17 × 10^9^	1.49 × 10^8^
	best	**100**	1.89 × 10^10^	249.9507	3.06 × 10^10^	10,293.31	9.38 × 10^9^	1.12 × 10^9^	348,397.6	2.29 × 10^8^	3.25 × 10^9^	2127.199	3140.724	1.11 × 10^8^
	worst	**100**	2.74 × 10^10^	6400.558	4.22 × 10^10^	33,968.45	2.04 × 10^10^	1.76 × 10^9^	570,375.9	4.19 × 10^9^	7.67 × 10^9^	30,586,212	4.68 × 10^9^	2.05 × 10^8^
	std	**8.43 × 10^−15^**	4.1 × 10^9^	2961.181	5.49 × 10^9^	11,751.58	5.27 × 10^9^	3.38 × 10^8^	112,689.2	1.93 × 10^9^	1.9 × 10^9^	15,098,863	2.4 × 10^9^	41,823,286
	median	**100**	2.07 × 10^10^	1901.307	3.22 × 10^10^	22,564.22	1.5 × 10^10^	1.4 × 10^9^	437,509.3	5.74 × 10^8^	4.83 × 10^9^	2,228,251	2,665,370	1.39 × 10^8^
	rank	**1**	12	2	13	3	11	9	4	8	10	5	7	6
C17-F3	mean	**300**	81,233.41	37,329.8	61,434.22	969.2666	39,412.41	193,462.9	1530.474	34,798.53	28,973.43	79,991.48	26,646.5	139,514.4
	best	**300**	74,187.68	20,290.72	47,586.37	759.5804	37,343.78	160,065.6	1213.136	30,406.4	24,674.27	68,874.59	19,026.26	105,582.7
	worst	**300**	89,185.68	48,259.67	66,731.14	1181.838	41,525.55	222,246.5	2083.641	38,857.75	31,375.4	88,084.82	34,209.61	193,825.8
	std	**0**	7603.304	12,317.32	9512.63	194.7523	2152.061	26,564.6	396.5439	3561.027	3099.143	8899.439	7103.868	43,017.94
	median	**300**	80,780.14	40,384.41	65,709.68	967.8242	39,390.15	195,769.8	1412.559	34,964.98	29,922.02	81,503.25	26,675.06	129,324.6
	rank	**1**	11	7	9	2	8	13	3	6	5	10	4	12
C17-F4	mean	**458.5616**	5454.175	505.3711	8271.423	487.4112	3867.585	790.9394	490.4413	553.0887	833.3965	572.1942	596.6992	753.4735
	best	**458.5616**	3096.677	486.3042	5328.848	478.6691	949.7377	736.6592	483.8844	506.6625	660.6389	555.214	506.2318	709.9571
	worst	**458.5616**	7356.503	520.5268	11,531.91	505.5639	6379.264	858.7068	501.689	579.2485	1167.212	591.7098	754.0269	773.3006
	std	**0**	1813.388	14.58486	2644.597	12.6856	2355.048	57.19553	8.087422	32.75768	232.9876	16.34613	116.8234	30.48773
	median	**458.5616**	5681.76	507.3267	8112.464	482.706	4070.67	784.1958	488.0959	563.2219	752.8677	570.9265	563.2689	765.3182
	rank	**1**	12	4	13	2	11	9	3	5	10	6	7	8
C17-F5	mean	**502.4874**	788.675	688.8314	821.4349	569.9836	746.1304	770.5924	600.2459	602.3281	726.4291	686.6635	611.2496	669.5814
	best	**500.995**	772.1701	658.1836	799.8365	551.0609	722.4248	746.244	587.598	568.2726	707.4411	670.418	590.5804	628.3879
	worst	**503.9798**	806.4481	737.7376	849.8832	588.9952	774.1923	781.9335	629.526	626.3604	748.2691	708.7927	652.7534	722.3131
	std	**1.319286**	14.65498	36.97029	24.54438	16.31498	25.13527	16.8415	20.18613	29.46007	20.29882	17.62707	28.98926	39.98674
	median	**502.4874**	788.041	679.7021	818.0099	569.9392	743.9522	777.0961	591.9299	607.3398	725.0032	683.7216	600.8324	663.8123
	rank	**1**	12	8	13	2	10	11	3	4	9	7	5	6
C17-F6	mean	**600**	666.4978	638.8884	669.1329	602.7229	664.118	663.4798	620.3129	609.9019	636.0709	647.051	639.0789	625.1306
	best	**600**	665.3934	637.2348	664.7104	601.6637	651.3463	654.3941	610.402	603.8971	630.1431	646.43	628.9354	619.2719
	worst	**600**	667.5953	641.4742	674.6851	603.8923	671.6263	667.9951	630.8845	615.81	645.7316	647.876	648.0269	628.9638
	std	**6.74 × 10^−14^**	0.932484	1.88193	4.731746	0.99459	9.794226	6.374473	9.903149	5.03593	7.038561	0.654628	8.680735	4.336506
	median	**600**	666.5013	638.4222	668.568	602.6678	666.7496	665.7649	619.9825	609.9503	634.2045	646.9491	639.6766	626.1434
	rank	**1**	12	7	13	2	11	10	4	3	6	9	8	5
C17-F7	mean	**733.478**	1204.463	1078.151	1238.467	828.2657	1142.796	1211.954	834.3939	860.7791	1019.025	932.0193	854.6732	928.9018
	best	**732.8186**	1164.527	981.5636	1227.053	805.6758	1021.289	1175.895	789.8107	801.9995	945.5342	892.7306	836.9552	895.7613
	worst	**734.5199**	1235.118	1213.361	1258.108	873.3993	1268.082	1279.518	896.1273	894.4226	1083.087	990.8162	877.4305	974.77
	std	**0.774451**	31.41462	105.2435	14.2092	31.48748	109.9771	49.47859	46.76886	41.54959	73.85197	44.20094	17.97128	33.98216
	median	**733.2867**	1209.104	1058.839	1234.354	816.9939	1140.907	1196.201	825.8188	873.347	1023.739	922.2653	852.1535	922.538
	rank	**1**	11	9	13	2	10	12	3	5	8	7	4	6
C17-F8	mean	**803.3298**	1038.449	925.7533	1069.623	876.5654	1016.808	992.9782	878.8546	877.7404	986.4922	935.6434	904.0603	956.231
	best	**801.2023**	1025.817	900.5847	1052.536	871.0406	979.7928	945.8382	853.3074	872.0014	970.7384	915.381	894.3161	942.8596
	worst	**804.1574**	1055.496	943.5544	1092.03	883.5203	1103.866	1028.054	903.9133	884.2305	1014.274	958.174	917.1052	973.4873
	std	**1.459319**	13.93036	20.10293	20.64133	5.327237	60.20137	36.04833	22.81096	5.469996	19.61526	19.47002	10.42038	15.81918
	median	**803.9798**	1036.242	929.4371	1066.963	875.8503	991.7858	999.0104	879.0988	877.3648	980.4783	934.5094	902.4099	954.2886
	rank	**1**	12	6	13	2	11	10	4	3	9	7	5	8
C17-F9	mean	**900**	9295.993	4173.342	9012.957	1054.217	9733.216	9347.75	4699.464	1882.552	4964.801	3552.341	3108.773	1228.1
	best	**900**	7964.504	3122.833	8794.493	924.8592	5988.134	7180.18	3773.249	1433.066	3627.866	3103.889	1915.365	1050.822
	worst	**900**	10,549.7	4734.648	9123.556	1181.76	13,095.64	11,118.81	7107.103	2539.514	7419.807	4243.209	4661.288	1404.044
	std	**6.74 × 10^−14^**	1104.682	740.9093	152.1923	121.9623	3015.762	2035.1	1652.669	551.4373	1762.665	515.4767	1196.496	170.3034
	median	**900**	9334.885	4417.945	9066.889	1055.124	9924.545	9546.007	3958.751	1778.814	4405.766	3431.134	2929.219	1228.767
	rank	**1**	11	7	10	2	13	12	8	4	9	6	5	3
C17-F10	mean	**2293.267**	6412.998	4935.853	6985.307	3713.286	5861.942	5808.81	4264.686	4380.976	7001.727	4430.57	4591.072	5513.346
	best	**1851.756**	5855.591	4355.06	6195.64	3430.104	4624.483	5082.697	3975.742	3968.251	6711.848	4160.289	4401.901	5126.171
	worst	**2525.027**	6695.583	5351.312	7529.713	4017.336	6380.625	6852.131	4623.226	4585.773	7167.196	4793.76	5012.506	5916.111
	std	308.512	389.0405	476.6797	581.2411	280.4434	851.4741	802.03	315.2446	291.3034	**210.3384**	296.6351	296.959	387.4678
	median	**2398.142**	6550.409	5018.519	7107.938	3702.853	6221.329	5650.205	4229.888	4484.939	7063.932	4384.117	4474.942	5505.55
	rank	**1**	11	7	12	2	10	9	3	4	13	5	6	8
C17-F11	mean	**1102.987**	6454.855	1232.689	7540.948	1158.917	4470.63	6715.96	1279.834	2016.264	1842.932	2604.326	1225.604	7847.806
	best	**1100.995**	5343.535	1176.99	6172.532	1119.088	3225.48	4877.451	1243.515	1342.749	1510.021	2056.133	1200.781	2992.546
	worst	**1105.977**	7366.873	1286.378	8465.077	1187.506	6656.979	9855.263	1314.717	3807.505	2457.777	3166.256	1248.844	14,582.53
	std	**2.210814**	913.6114	46.85703	1078.653	30.27007	1583.349	2228.596	41.35443	1228.211	431.1576	537.3454	23.86209	5101.831
	median	**1102.487**	6554.506	1233.693	7763.091	1164.537	4000.031	6065.562	1280.551	1457.402	1701.966	2597.458	1226.396	6908.076
	rank	**1**	10	4	12	2	9	11	5	7	6	8	3	13
C17-F12	mean	**1744.553**	5.88 × 10^9^	17,450,698	9.13 × 10^9^	18,387.77	4.24 × 10^9^	2.07 × 10^8^	9,395,467	43,971,467	2.53 × 10^8^	1.67 × 10^8^	2,145,195	6,431,729
	best	**1721.81**	4.86 × 10^9^	2,455,842	8.13 × 10^9^	13,217.15	2.18 × 10^9^	52,999,424	4,362,972	4,268,282	1.62 × 10^8^	32,203,594	232,101.1	4,453,485
	worst	**1764.937**	7.46 × 10^9^	42,619,251	1.15 × 10^10^	23,387.01	5.55 × 10^9^	4.14 × 10^8^	22,731,848	92,210,162	4.39 × 10^8^	5.32 × 10^8^	4,264,822	8,418,583
	std	**20.69875**	1.14 × 10^9^	18,158,260	1.64 × 10^9^	4451.061	1.49 × 10^9^	1.71 × 10^8^	9,145,700	39,378,893	1.29 × 10^8^	2.5 × 10^8^	1,786,631	1,847,128
	median	**1745.733**	5.59 × 10^9^	12,363,849	8.44 × 10^9^	18,473.46	4.62 × 10^9^	1.81 × 10^8^	5,243,524	39,703,712	2.06 × 10^8^	51,055,526	2,041,929	6,427,424
	rank	**1**	12	6	13	2	11	9	5	7	10	8	3	4
C17-F13	mean	**1315.791**	4.78 × 10^9^	125,372.3	8.82 × 10^9^	1795.796	1.22 × 10^9^	756,831.1	76,309.37	631,742.6	73,765,671	30,734.88	27,296.78	9,967,217
	best	**1314.587**	2.33 × 10^9^	69,505.05	4.63 × 10^9^	1565.812	16,503,809	357,223.2	30,683.32	76,461.92	51,226,748	24,971.07	11,416.68	2,704,385
	worst	**1318.646**	6.69 × 10^9^	198,170	1.08 × 10^10^	2245.82	4.25 × 10^9^	1,118,905	153,076.9	1,959,916	1.09 × 10^8^	44,874.71	61,408.81	21,439,242
	std	**1.988738**	1.86 × 10^9^	54879	2.91 × 10^9^	315.5748	2.09 × 10^9^	407,890.3	59,034.82	921,235.2	25,573,819	9789.797	23,630.35	8,246,276
	median	**1314.967**	5.05 × 10^9^	116,907.1	9.92 × 10^9^	1685.776	3.15 × 10^8^	775,598	60,738.64	245,296.1	67,531,342	26,546.87	18,180.81	7,862,621
	rank	**1**	12	6	13	2	11	8	5	7	10	4	3	9
C17-F14	mean	**1423.017**	1,583,673	226,835.8	1,835,217	1437.554	981,560.3	1,857,980	17,224.06	445,626.9	117,098.7	955,849.2	15,909.45	1,677,408
	best	**1422.014**	976,681.6	31,922.39	922,514.9	1434.585	702,426.8	30,232.29	4403.604	28,944.95	68,150.13	620,319.3	2886.6	277,836.4
	worst	**1423.993**	2,004,673	524,857.3	2,732,711	1441.555	1,386,589	5,675,562	29,161.92	954,830.9	134,697.7	1,443,086	28,859.22	2,827,823
	std	**0.830071**	494,194.3	223,386.9	894,383.3	3.225506	322,490.9	2,662,657	10,953.49	482,778.6	33,529.54	397,688.3	11,653.16	1,207,941
	median	**1423.03**	1,676,669	175,281.7	1,842,822	1437.038	918,612.9	863,063.4	17,665.35	399,365.9	132,773.6	879,995.7	15,945.99	1,801,986
	rank	**1**	10	6	12	2	9	13	4	7	5	8	3	11
C17-F15	mean	**1503.129**	2.54 × 10^8^	31,519.17	4.99 × 10^8^	1599.839	12,002,926	4,212,356	35,971.42	13,215,344	4,286,963	13,666.1	4238.662	798,198
	best	**1502.462**	2.2 × 10^8^	9374.391	4.31 × 10^8^	1568.393	4,728,541	194,271.7	20,925.85	82,288.08	973,598.4	9778.652	1846.111	146,710.5
	worst	**1504.265**	2.81 × 10^8^	51,033.83	5.51 × 10^8^	1613.784	27,920,922	13,676,518	59,349.91	49,479,680	8,069,607	18,446.14	7667.485	1,788,072
	std	**0.878736**	31,317,095	18,070.21	60,593,995	21.68729	10,995,701	6,568,827	17,104.85	24,843,447	2,988,423	3721.739	2648.141	771,437.9
	median	**1502.893**	2.58 × 10^8^	32,834.22	5.07 × 10^8^	1608.589	7,681,119	1,489,318	31,804.95	1,649,704	4,052,323	13,219.8	3720.525	629,004.7
	rank	**1**	12	5	13	2	10	8	6	11	9	4	3	7
C17-F16	mean	**1663.469**	3880.794	2780.727	4429.79	1967.727	3007.036	3818.944	2436.145	2399.236	3167.806	3336.348	2722.277	2738.09
	best	**1614.72**	3612.046	2400.514	3787.816	1713.465	2646.388	3186.838	2248.521	2266.9	2999.485	3173.393	2527.152	2442.737
	worst	**1744.118**	4112.827	3219.231	5016.989	2173.201	3223.217	4523.221	2651.18	2497.872	3357.115	3491.913	2958.075	3023.933
	std	**63.65095**	235.5644	345.9519	676.9997	211.8471	263.5324	566.1256	178.2288	118.7481	160.2248	145.9345	225.7184	288.5744
	median	**1647.519**	3899.152	2751.581	4457.177	1992.122	3079.27	3782.858	2422.44	2416.086	3157.312	3340.043	2701.941	2742.844
	rank	**1**	12	7	13	2	8	11	4	3	9	10	5	6
C17-F17	mean	**1728.099**	3134.688	2354.083	3389.138	1842.605	3022.162	2667.351	2025.542	1901.862	2120.603	2395.923	2238.525	2088.621
	best	**1718.761**	2631.554	2231.916	3070.875	1748.388	2142.768	2266.432	1981.833	1792.086	1929.693	2310.34	2040.211	2049.706
	worst	**1733.659**	3757.185	2449.769	3953.478	1894.947	5325.838	2938.747	2151.766	2027.281	2368.201	2522.618	2569.374	2147.494
	std	**6.88979**	492.2332	98.22265	410.9112	66.42675	1579.623	295.4104	86.44565	114.642	190.9079	105.9575	243.7852	46.51756
	median	**1729.987**	3075.006	2367.323	3266.098	1863.543	2310.021	2732.113	1984.285	1894.041	2092.259	2375.366	2172.258	2078.642
	rank	**1**	12	8	13	2	11	10	4	3	6	9	7	5
C17-F18	mean	**1825.696**	23,729,550	2,212,009	27,283,974	1885.211	30,340,275	4,927,404	534,595.3	350,552.7	1,391,192	430,211.5	114,852.6	3,044,058
	best	**1822.524**	6,835,900	235,823.5	8,821,140	1866.68	1,112,837	1,660,690	134,742.3	65,780.31	646,005.5	241,319.2	81,807.06	2,376,553
	worst	**1828.42**	46,083,790	4,413,170	53,602,004	1896.441	57,496,084	10,169,814	1,446,703	900,247.2	1,748,911	837,349.8	136,220	4,461,888
	std	**2.775128**	17,820,592	2,010,817	19,503,687	13.58535	32,158,616	3,755,642	628,519	403,355.5	520,829.7	282,413.8	24,434.14	981,973
	median	**1825.92**	20,999,256	2,099,521	23,356,376	1888.861	31,376,090	3,939,556	278,467.7	218,091.7	1,584,927	321,088.5	120,691.7	2,668,895
	rank	**1**	11	8	12	2	13	10	6	4	7	5	3	9
C17-F19	mean	**1910.989**	4.85 × 10^8^	56,833.4	8.17 × 10^8^	1921.731	2.46 × 10^8^	11,962,405	784,633.2	3,367,690	4,802,159	68,581.12	37,464.21	1,353,797
	best	**1908.84**	3.63 × 10^8^	12,363.24	5.9 × 10^8^	1919.401	3,053,269	1,556,878	20,101.42	59,431.52	2,492,766	37,340.59	7629.39	535,107.5
	worst	**1913.095**	6.31 × 10^8^	126,214.8	1.24 × 10^9^	1925.522	6.81 × 10^8^	20,655,538	1,763,741	10,858,942	6,826,083	92,183.96	111,581	2,404,782
	std	**1.984351**	1.38 × 10^8^	50,937.84	2.95 × 10^8^	2.730401	3.21 × 10^8^	8,945,361	871,528.6	5,164,447	2,189,230	23,450.06	50,925.51	809,908.7
	median	**1911.01**	4.73 × 10^8^	44,377.77	7.2 × 10^8^	1921.001	1.5 × 10^8^	12,818,601	677,345.4	1,276,194	4,944,894	72,399.97	15,323.22	1,237,648
	rank	**1**	12	4	13	2	11	10	6	8	9	5	3	7
C17-F20	mean	**2065.787**	2766.527	2545.205	2811.832	2159.069	2725.378	2714.989	2519.588	2326.117	2681.848	2859.476	2470.911	2410.483
	best	**2029.521**	2685.094	2405.958	2657.449	2056.898	2598.867	2558.17	2320.368	2175.006	2617.435	2539.35	2423.632	2364.938
	worst	**2161.126**	2858.293	2738.536	2900.126	2248.615	2848.489	2862.147	2876.053	2465.314	2781.176	3277.45	2576.545	2452.06
	std	65.37076	72.89626	145.7221	111.8625	81.15997	105.2842	133.9402	251.4598	122.4135	80.36502	318.3825	73.05095	**37.17676**
	median	**2036.25**	2761.36	2518.162	2844.877	2165.382	2727.077	2719.818	2440.965	2332.073	2664.39	2810.552	2441.733	2412.468
	rank	**1**	11	7	12	2	10	9	6	3	8	13	5	4
C17-F21	mean	**2308.456**	2572.955	2420.169	2621.231	2357.321	2499.307	2562.737	2390.021	2377.222	2466.006	2528.957	2414.753	2463.582
	best	2304.034	2493.073	**2232.502**	2554.499	2348.478	2307.582	2498.155	2359.397	2347.988	2455.094	2512.979	2398.004	2435.213
	worst	**2312.987**	2625.981	2553.058	2700.04	2371.332	2612.772	2617.742	2414.764	2389.966	2475.425	2559.852	2426.276	2506.717
	std	**4.579845**	64.4505	138.5267	65.15362	10.30986	138.3342	60.89915	23.7242	20.46152	10.28472	21.56359	14.12901	31.25484
	median	**2308.402**	2586.384	2447.558	2615.193	2354.736	2538.437	2567.526	2392.961	2385.467	2466.752	2521.499	2417.367	2456.2
	rank	**1**	12	6	13	2	9	11	4	3	8	10	5	7
C17-F22	mean	**2300**	7084.865	5222.881	6880.473	2302.464	7749.913	6598.274	3692.769	2640.103	5149.572	5690.404	4475.754	2638.563
	best	**2300**	6799.132	2302.572	6013.125	2301.607	7555.353	5792.796	2305.473	2531.242	2656.684	3732.182	2432.779	2575.489
	worst	**2300**	7530.718	6351.172	7752.205	2303.911	7841.768	7316.866	5415.585	2863.893	7913.53	6551.003	6452.917	2687.306
	std	**0**	321.1263	2002.835	767.6903	1.061178	138.2371	650.5143	1668.406	156.2621	2939.887	1349.643	1898.886	56.91863
	median	**2300**	7004.805	6118.891	6878.281	2302.169	7801.265	6641.717	3525.01	2582.639	5014.037	6239.216	4508.66	2645.728
	rank	**1**	12	8	11	2	13	10	5	4	7	9	6	3
C17-F23	mean	2655.081	3109.491	2885.363	3155.951	**2647.452**	3113.614	2987.155	2724.949	2736.577	2865.867	3597.108	2863.007	2926.383
	best	2653.745	3036.821	2792.667	3110.28	**2499.658**	3013.031	2837.165	2687.2	2719.693	2847.605	3505.506	2834.598	2901.442
	worst	**2657.377**	3178.552	3031.739	3222.42	2703.755	3280.56	3071.559	2749.403	2754.474	2908.217	3687.511	2906.401	2980.154
	std	**1.697988**	68.35427	107.5776	50.12905	101.4466	121.159	106.8838	27.37701	15.40831	29.53252	98.94313	33.94444	37.1707
	median	**2654.6**	3111.295	2858.524	3145.551	2693.198	3080.433	3019.947	2731.596	2736.071	2853.823	3597.708	2855.513	2911.968
	rank	2	10	7	12	**1**	11	9	3	4	6	13	5	8
C17-F24	mean	**2831.409**	3241.513	3119.205	3326.308	2875.638	3212.496	3073.051	2894.483	2907.337	3010.226	3283.04	3085.487	3166.225
	best	**2829.992**	3209.481	3001.28	3250.677	2862.509	3119.874	3018.482	2853.563	2896.635	2990.26	3251.748	3021.176	3086.14
	worst	**2832.366**	3308.124	3251.029	3457.948	2881.393	3255.852	3095.358	2913.975	2913.166	3041.037	3315.281	3182.405	3233.528
	std	**1.176599**	46.17052	112.6287	98.47879	9.095286	65.19122	37.54706	28.41498	7.691137	22.24128	28.82213	71.00122	70.21629
	median	**2831.64**	3224.223	3112.255	3298.303	2879.325	3237.129	3089.182	2905.197	2909.774	3004.803	3282.564	3069.184	3172.615
	rank	**1**	11	8	13	2	10	6	3	4	5	12	7	9
C17-F25	mean	**2886.698**	3778.564	2905.323	4308.259	2890.58	3380.167	3051.922	2906.017	2976.962	3045.87	2978.771	2893.691	3073.77
	best	2886.691	3459.543	2893.27	3796.357	2884.863	3059.868	3021.001	**2884.861**	2944.721	2943.505	2968.969	2887.465	3059.545
	worst	**2886.707**	4017.439	2938.478	4990.555	2895.827	3712.732	3068.179	2960.374	3036.993	3162.281	2989.401	2908.295	3083.95
	std	**0.007812**	239.2238	22.7185	510.8381	5.088898	327.7512	22.78511	37.31992	43.94737	107.454	8.665444	10.04843	11.07641
	median	**2886.698**	3818.637	2894.771	4223.061	2890.815	3374.033	3059.254	2889.418	2963.067	3038.848	2978.357	2889.502	3075.793
	rank	**1**	12	4	13	2	11	9	5	6	8	7	3	10
C17-F26	mean	3578.65	8312.608	6748.721	8809.795	**3047.747**	7936.265	7634.154	4600.167	4412.45	5561.102	6871.666	4652.067	4267.871
	best	3559.841	7952.123	5663.995	8103.995	**3043.527**	7376.99	7009.695	4310.505	4075.318	4388.821	5978.83	3551.522	3939.486
	worst	3607.686	8958.698	7389.877	10,063.25	**3054.149**	8287.494	8358.253	5135.989	4931.721	6669.841	7336.144	5966.081	4664.532
	std	23.3936	481.0495	778.935	945.3017	**5.206666**	401.4653	568.6926	395.2051	374.9892	1073.57	649.2089	1158.03	312.2156
	median	3573.536	8169.805	6970.507	8535.966	**3046.657**	8040.289	7584.334	4477.086	4321.379	5592.874	7085.844	4545.332	4233.733
	rank	2	12	8	13	**1**	11	10	5	4	7	9	6	3
C17-F27	mean	**3207.018**	3548.906	3332.744	3680.504	3213.451	3432.917	3393.896	3227.682	3243.298	3301.015	4702.192	3267.723	3421.148
	best	**3200.749**	3499.138	3259.867	3442.317	3202.083	3318.525	3250.554	3212.231	3234.816	3234.55	4320.546	3234.544	3356.112
	worst	**3210.656**	3633.364	3396.858	3923.406	3229.739	3644.348	3500.711	3249.91	3256.502	3362.958	4979.658	3304.29	3460.169
	std	**4.773736**	61.58738	73.72286	211.864	13.2029	149.0222	110.3439	16.30374	9.514926	54.62672	331.9083	31.12272	46.40822
	median	**3208.335**	3531.561	3337.126	3678.146	3210.992	3384.398	3412.159	3224.293	3240.938	3303.276	4754.281	3266.03	3434.155
	rank	**1**	11	7	12	2	10	8	3	4	6	13	5	9
C17-F28	mean	**3100**	4523.86	3240.506	5294.879	3196.528	3996.778	3386.937	3232.705	3521.426	3582.827	3457.186	3294.198	3509.347
	best	**3100**	4323.582	3214.275	5032.036	3182.459	3523.101	3335.125	3202.018	3352.242	3455.219	3396.665	3179.941	3465.061
	worst	**3100**	4740.947	3267.785	5570.181	3222.193	4479.995	3433.761	3260.726	3934.763	3874.887	3584.199	3469.229	3557.427
	std	**2.7 × 10^−13^**	183.7042	22.46907	264.0432	18.23778	455.0824	44.09313	24.78707	284.7302	202.2402	88.03279	137.5115	45.08074
	median	**3100**	4515.456	3239.983	5288.649	3190.73	3992.009	3389.432	3234.039	3399.349	3500.6	3423.941	3263.81	3507.45
	rank	**1**	12	4	13	2	11	6	3	9	10	7	5	8
C17-F29	mean	**3353.75**	5087.826	4182.435	5274.316	3611.29	4953.596	4822.947	3768.517	3724.077	4334.459	4802.573	4044.32	4145.088
	best	**3325.385**	4705.704	3878.416	4739.449	3481.774	4488.326	4594.079	3656.101	3654.319	4051.046	4571.263	3877.567	3815.679
	worst	**3370.797**	5496.181	4365.531	6006.428	3731.924	5708.04	4971.577	3872.603	3822.108	4745.9	5022.554	4253.432	4448.855
	std	**20.22231**	390.4582	222.0926	640.5091	114.0033	585.6057	165.544	94.16507	76.66849	304.191	246.5089	159.5929	290.9356
	median	**3359.41**	5074.711	4242.896	5175.694	3615.731	4809.01	4863.066	3772.683	3709.94	4270.446	4808.237	4023.14	4157.909
	rank	**1**	12	7	13	2	11	10	4	3	8	9	5	6
C17-F30	mean	**5007.854**	1.2 × 10^9^	1,198,183	2.37 × 10^9^	7256.376	32,265,568	32,925,121	2,597,860	5,356,352	31,786,061	1,900,575	229,623.9	590,194.9
	best	**4955.449**	8.85 × 10^8^	423,016.4	1.7 × 10^9^	6158.427	11,031,639	6,566,732	467,071	1,195,436	17,015,110	1,659,159	7179.109	163,864.3
	worst	**5086.396**	1.32 × 10^9^	2,121,157	2.62 × 10^9^	9416.184	75,389,656	52,758,912	3,719,132	14,462,906	66,673,306	2,286,605	867,053.8	1,128,316
	std	**60.57214**	2.17 × 10^8^	729,213.9	4.58 × 10^8^	1570.007	30,004,969	19,776,654	1,489,740	6,293,655	24,022,890	277,434.8	436,680.2	482,284
	median	**4994.785**	1.3 × 10^9^	1,124,279	2.58 × 10^9^	6725.446	21,320,489	36,187,421	3,102,619	2,883,533	21,727,915	1,828,269	22,131.29	534,300
	rank	**1**	12	5	13	2	10	11	7	8	9	6	3	4
Sum rank		**31**	334	182	361	57	305	284	128	151	232	231	139	204
Mean rank		**1.068966**	11.51724	6.275862	12.44828	1.965517	10.51724	9.793103	4.413793	5.206897	8	7.965517	4.793103	7.034483
Total rank		**1**	12	6	13	2	11	10	3	5	9	8	4	7

**Table 4:** Optimization results of CEC 2017 test suite (dimension = 50); background color has been used in order to make the table more reader-friendly and to separate the results of benchmark functions from each other; The best results are specified using bold.

		POA	WSO	AVOA	RSA	MPA	TSA	WOA	MVO	GWO	TLBO	GSA	PSO	GA
C17-F1	mean	**100**	4.98 × 10^10^	7,694,163	7.8 × 10^10^	4,687,637	3.17 × 10^10^	6.41 × 10^9^	3,383,888	7.78 × 10^9^	1.73 × 10^10^	1.43 × 10^10^	2.11 × 10^9^	8.65 × 10^9^
	best	**100**	4.44 × 10^10^	916,036.2	6.82 × 10^10^	1,809,188	2.92 × 10^10^	3.78 × 10^9^	2,421,905	5.61 × 10^9^	1.17 × 10^10^	1.14 × 10^10^	8.65 × 10^8^	8.24 × 10^9^
	worst	**100**	5.33 × 10^10^	20,360,589	8.52 × 10^10^	11,885,877	3.41 × 10^10^	9.59 × 10^9^	4,212,013	1.07 × 10^10^	2.33 × 10^10^	1.71 × 10^10^	2.81 × 10^9^	9.32 × 10^9^
	std	**0**	4.01 × 10^9^	8,873,644	7.62 × 10^9^	4,964,317	2.09 × 10^9^	2.82 × 10^9^	756,708.6	2.16 × 10^9^	5.76 × 10^9^	2.39 × 10^9^	8.78 × 10^8^	5.21 × 10^8^
	median	**100**	5.07 × 10^10^	4,750,013	7.92 × 10^10^	2,527,742	3.18 × 10^10^	6.13 × 10^9^	3,450,817	7.44 × 10^9^	1.7 × 10^10^	1.43 × 10^10^	2.38 × 10^9^	8.53 × 10^9^
	rank	**1**	12	4	13	3	11	6	2	7	10	9	5	8
C17-F3	mean	**300**	131,835.8	121,939.9	131,347.9	14,965.02	90,792.26	194,620.8	38,514.36	108,129.9	81,796.38	148,054.1	120,427.1	219,121.3
	best	**300**	113,081.2	93,706.17	119,156.7	12,934.34	79,774.46	146,792.7	30,547.5	95,001.03	61,871.66	133,707	90,521.94	182,644.6
	worst	**300**	151,612.4	148,352	143,174.6	17,653.06	96,796.99	296,860.1	47,889.01	121,370	93,323.17	167,270.6	156,910.3	251,776.2
	std	**0**	16,662.92	25,349.19	10,952.68	2175.535	8080.823	72,592.69	7427.946	11,067.16	14,750.72	16,664.14	29,574.49	29,078.14
	median	**300**	131,324.8	122,850.8	131,530.3	14,636.35	93,298.79	167,415.2	37,810.47	108,074.4	85,995.34	145,619.5	117,138.1	221,032.1
	rank	**1**	10	8	9	2	5	12	3	6	4	11	7	13
C17-F4	mean	**470.3679**	12,353.44	659.3073	19,835.21	520.8641	6991.207	1692.379	547.5027	1273.318	2403.23	2627.084	924.1037	1347.608
	best	**428.5127**	9616.635	640.999	13,121.57	484.6049	5610.825	1108.464	510.0485	960.669	1386.37	2200.404	641.0513	1168.399
	worst	**525.7252**	14,063.24	675.5487	23,684.52	573.5045	9011.58	2005.852	605.8885	1535.439	4062.787	2789.659	1582.677	1455.999
	std	50.91462	2040.528	**16.66823**	4949.499	43.26782	1475.615	412.7291	42.51684	267.1199	1208.173	293.3794	453.5134	128.9881
	median	**463.6168**	12,866.95	660.3407	21,267.38	512.6735	6671.212	1827.6	537.0369	1298.581	2081.882	2759.135	736.3432	1383.016
	rank	**1**	12	4	13	2	11	8	3	6	9	10	5	7
C17-F5	mean	**504.7261**	998.7714	797.874	1023.055	696.7875	1037.896	879.3746	698.9245	687.8787	914.9902	754.902	740.7178	826.1298
	best	**503.9798**	971.6799	772.4922	1007.523	628.9187	919.5867	845.571	637.7742	665.0593	880.0784	711.1297	695.3349	801.0767
	worst	**505.9698**	1032.226	832.4122	1033.829	750.0726	1132.417	900.3438	792.9468	711.594	937.7921	785.3274	794.0634	843.8261
	std	**0.978409**	29.84483	26.55012	12.4602	52.17824	106.2494	25.07713	71.31889	25.48733	26.49006	35.95038	41.72614	20.88025
	median	**504.4773**	995.59	793.2958	1025.434	704.0793	1049.791	885.7917	682.4885	687.4307	921.0452	761.5754	736.7365	829.8082
	rank	**1**	11	7	12	3	13	9	4	2	10	6	5	8
C17-F6	mean	**600**	678.7743	649.8125	680.4897	609.3938	674.4157	681.0236	631.1676	618.8765	653.1538	647.9508	644.3503	640.179
	best	**600**	676.2919	645.8267	678.6046	607.0893	657.5593	676.5779	622.7099	614.1754	642.6254	643.8879	642.4606	629.4886
	worst	**600**	682.8851	654.3317	682.9158	612.4396	688.2301	687.8313	651.0589	627.047	660.3253	650.3646	647.2969	650.6368
	std	**0**	3.126532	4.035656	2.072733	2.353657	13.99634	5.020365	13.86957	5.932199	7.779141	2.927148	2.245176	9.089876
	median	**600**	677.9601	649.5457	680.2192	609.0231	675.9367	679.8427	625.4507	617.1418	654.8323	648.7754	643.8218	640.2954
	rank	**1**	11	8	12	2	10	13	4	3	9	7	6	5
C17-F7	mean	**756.7298**	1615.316	1510.391	1698.01	982.2029	1524.212	1545.236	1003.011	1012.807	1355.063	1298.967	1123.67	1212.925
	best	**754.7543**	1594.636	1451.321	1630.929	934.7738	1397.907	1493.027	971.7793	993.2567	1249.798	1158.917	991.2395	1147.95
	worst	**758.3522**	1641.438	1565.593	1783.808	1022.213	1648.02	1617.358	1028.408	1028.664	1406.086	1406.26	1316.364	1255.178
	std	**1.595411**	20.01803	49.79641	67.43425	43.39772	119.7991	59.49773	24.56744	16.92836	73.00096	114.5055	144.1241	48.62485
	median	**756.9065**	1612.595	1512.325	1688.651	985.9122	1525.461	1535.28	1005.929	1014.653	1382.184	1315.346	1093.538	1224.285
	rank	**1**	12	9	13	2	10	11	3	4	8	7	5	6
C17-F8	mean	**805.721**	1315.026	1069.907	1337.934	975.5652	1329.453	1236.115	985.028	994.979	1233.995	1083.154	1013.704	1180.568
	best	**802.9849**	1267.365	1031.039	1311.392	949.5886	1246.589	1125	952.6691	965.8927	1187.074	1075.982	977.9443	1146.507
	worst	**810.9446**	1351.319	1110.142	1355.805	1001.572	1440.841	1328.075	1043.891	1026.654	1281.178	1095.97	1068.267	1200.173
	std	**3.672737**	39.08297	45.64879	19.32054	27.95425	86.06199	86.03151	41.35436	27.97589	39.98849	9.254532	43.73755	24.0814
	median	**804.4773**	1320.71	1069.223	1342.271	975.5499	1315.192	1245.693	971.7758	993.6848	1233.865	1080.331	1004.301	1187.795
	rank	**1**	11	6	13	2	12	10	3	4	9	7	5	8
C17-F9	mean	**900**	30,080.05	11,149.25	30,239.59	2906.627	31,546.08	27,482.14	16,432.75	5843.369	20,055.75	8985.748	8670.898	10,745.45
	best	**900**	28,896.41	10,632.96	28,419.95	1871.357	29,089.26	25,588.4	8863.034	5099.754	15,480.28	8200.157	8043.964	8867.123
	worst	**900**	32,836.97	11,866.94	31,723.8	4145.523	35,169.81	32,120.64	21,685.31	6634.453	23,572.38	9691.888	9834.376	12,353.76
	std	**9.53 × 10^−14^**	1918.301	548.1654	1606.899	964.9071	2690.339	3186.233	6193.736	818.5837	3451.708	636.8787	826.9251	1897.767
	median	**900**	29,293.4	11,048.55	30,407.3	2804.813	30,962.63	26,109.75	17,591.33	5819.635	20,585.17	9025.474	8402.626	10,880.47
	rank	**1**	11	7	12	2	13	10	8	3	9	5	4	6
C17-F10	mean	**4347.157**	11,532.14	7659.93	12,543.53	6175.008	10,518.17	10,524.61	7108.216	7941.727	12,367.61	7886.242	7215.482	10,459.41
	best	**3555.132**	10,983.86	7189.875	12,178.38	5457.78	9682.889	9353.445	5887.021	6184.118	11,645.95	7154.486	6946.725	10,015.5
	worst	**5099.795**	12,270.5	7977.589	12,987.68	6806.409	11,524.62	11,596.38	8039.728	12,231.37	12865	8919.848	7634.923	11,036.08
	std	662.2242	616.1048	346.5361	367.537	628.0243	833.1579	1005.392	939.4287	2967.615	603.7201	761.6	**303**	437.5358
	median	**4366.851**	11,437.1	7736.128	12,504.04	6217.921	10,432.59	10,574.3	7253.058	6675.708	12,479.74	7735.318	7140.14	10,393.04
	rank	**1**	11	5	13	2	9	10	3	7	12	6	4	8
C17-F11	mean	**1128.435**	12,982.43	1525.243	17,632.41	1233.86	10,936.77	4425.966	1494.635	5284.769	4439.173	11,990.04	1580.223	20,145.96
	best	**1121.25**	11,976.46	1426.183	15,706.17	1192.498	9426.317	3923.366	1369.325	3249.087	4175.03	11,255.98	1353.431	11,853.62
	worst	**1133.132**	13,620.52	1651.403	19,094.94	1259.489	13,091.47	5491.437	1620.754	9037.193	4919.792	13,565.41	1850.293	26,952.06
	std	**5.590435**	746.9688	107.1157	1454.228	30.74393	1622.687	740.6431	112.3004	2746.288	352.4488	1090.518	218.3901	6413.416
	median	**1129.678**	13,166.37	1511.693	17,864.27	1241.727	10,614.66	4144.53	1494.231	4426.397	4330.935	11,569.38	1558.583	20,889.09
	rank	**1**	11	4	12	2	9	6	3	8	7	10	5	13
C17-F12	mean	**2905.102**	3.63 × 10^10^	61,090,788	5.93 × 10^10^	11,987,913	2.15 × 10^10^	1.1 × 10^9^	65,947,360	7.97 × 10^8^	4.21 × 10^9^	1.81 × 10^9^	1.34 × 10^9^	1.7 × 10^8^
	best	**2527.376**	3.05 × 10^10^	25,877,157	4.32 × 10^10^	11,292,468	9.08 × 10^9^	9.08 × 10^8^	35,519,720	1.25 × 10^8^	2.37 × 10^9^	5.95 × 10^8^	10,571,947	53,696,816
	worst	**3168.37**	4.35 × 10^10^	94,397,377	8.13 × 10^10^	12,550,029	3.62 × 10^10^	1.5 × 10^9^	1.05 × 10^8^	1.48 × 10^9^	8.27 × 10^9^	3.25 × 10^9^	3.86 × 10^9^	2.36 × 10^8^
	std	**281.1232**	6.05 × 10^9^	37,699,246	1.8 × 10^10^	602,939	1.15 × 10^10^	2.78 × 10^8^	29,972,887	6.95 × 10^8^	2.84 × 10^9^	1.13 × 10^9^	1.84 × 10^9^	82,068,331
	median	**2962.331**	3.56 × 10^10^	62,044,309	5.63 × 10^10^	12,054,577	2.04 × 10^10^	9.97 × 10^8^	61,665,741	7.91 × 10^8^	3.09 × 10^9^	1.69 × 10^9^	7.36 × 10^8^	1.96 × 10^8^
	rank	**1**	12	3	13	2	11	7	4	6	10	9	8	5
C17-F13	mean	**1340.1**	2.05 × 10^10^	124,306	3.59 × 10^10^	13,820.12	8.4 × 10^9^	79,029,047	201,080.5	2.97 × 10^8^	4.87 × 10^8^	15,429,091	3.97 × 10^8^	34,568,913
	best	**1333.781**	1.18 × 10^10^	28,752.02	1.81 × 10^10^	7417.988	4.46 × 10^9^	59,414,617	125,544.1	1.35 × 10^8^	3.97 × 10^8^	26,219.81	42,641.1	22,533,768
	worst	**1343.015**	2.79 × 10^10^	273,649	5.16 × 10^10^	16,228.46	1.31 × 10^10^	89,739,751	313,654.4	7.48 × 10^8^	6.66 × 10^8^	52,008,316	1 × 10^9^	46,202,757
	std	**4.398296**	7.27 × 10^9^	107,558.8	1.44 × 10^10^	4388.247	3.74 × 10^9^	13,773,809	82,205.87	3.09 × 10^8^	1.24 × 10^8^	25,476,621	5.03 × 10^8^	10,856,438
	median	**1341.801**	2.11 × 10^10^	97,411.5	3.69 × 10^10^	15,817.02	8.03 × 10^9^	83,480,911	182,561.7	1.53 × 10^8^	4.43 × 10^8^	4,840,914	2.93 × 10^8^	34,769,564
	rank	**1**	12	3	13	2	11	7	4	8	10	5	9	6
C17-F14	mean	**1429.458**	21,629,558	1,019,175	40,326,460	1540.597	2,239,125	3,972,952	159,269.4	959,763.4	721,378.3	12,622,182	478,448.8	9,341,412
	best	**1425.995**	7,065,176	315,847.7	12,368,356	1529.06	591,649.7	3,517,514	100,976.4	74,947.42	594,856.7	2,861,955	172,016.9	4,596,836
	worst	**1431.939**	42,342,695	2,427,168	81,648,276	1561.417	3,551,308	4,721,198	308,929.3	1,851,840	832,267.8	20,724,323	766,155.9	16,077,250
	std	**2.692311**	15,290,806	985,718.2	30,267,916	15.13539	1,260,429	533,876.2	102,826.2	745,089.6	127,191.3	8,317,365	249,747.6	4,978,439
	median	**1429.95**	18,555,180	666,841	33,644,603	1535.955	2,406,771	3,826,549	113,586	956,132.9	729,194.4	13,451,225	487,811.2	8,345,781
	rank	**1**	12	7	13	2	8	9	3	6	5	11	4	10
C17-F15	mean	**1530.66**	2.17 × 10^9^	31,917.24	3.49 × 10^9^	2139.059	1.42 × 10^9^	8,268,173	101,399.5	4,957,875	58,803,800	1.65 × 10^8^	9300.399	7,146,830
	best	**1526.359**	1.54 × 10^9^	19,800.39	2.72 × 10^9^	2028.222	4.88 × 10^8^	762,321.9	42,127.59	35528	34,481,112	16,215.58	2567.105	2,428,749
	worst	**1532.953**	2.84 × 10^9^	58,561.65	4.13 × 10^9^	2261.16	3.09 × 10^9^	15,438,015	151,119.9	13,057,937	76,544,059	6.38 × 10^8^	18,031.22	15,510,530
	std	**3.013514**	6.32 × 10^8^	18,454.03	6.41 × 10^8^	126.8756	1.24 × 10^9^	6,625,549	49,760.11	5,836,236	18,067,125	3.25 × 10^8^	7059.598	5,942,171
	median	**1531.664**	2.15 × 10^9^	24,653.45	3.55 × 10^9^	2133.428	1.05 × 10^9^	8,436,177	106,175.3	3,369,019	62,095,015	9,951,567	8301.635	5,324,020
	rank	**1**	12	4	13	2	11	8	5	6	9	10	3	7
C17-F16	mean	**2062.891**	5581.005	3973.119	6665.639	2638.306	4212.793	4921.738	3117.378	3114.958	4131.227	3641.003	3128.543	3606.047
	best	**1728.601**	4843.246	3647.374	5095.094	2465.812	3691.913	4094.726	2941.736	2798.314	3806.364	3373.11	2808.407	3042.777
	worst	**2242.663**	7043.283	4340.84	9804.628	2866.62	4493.657	5496.223	3348.255	3623.096	4333.498	4005.457	3509.443	4074.333
	std	239.2227	1047.135	333.2	2222.094	183.478	370.2944	622.3757	**173.8423**	404.3577	234.4206	326.9663	379.38	461.5267
	median	**2140.15**	5218.746	3952.13	5881.418	2610.396	4332.801	5048.001	3089.76	3019.212	4192.523	3592.723	3098.161	3653.54
	rank	**1**	12	8	13	2	10	11	4	3	9	7	5	6
C17-F17	mean	**2021.151**	6688.485	3302.53	9549.641	2469.247	3635.175	4115.561	2895.834	2810.65	3794.819	3520.846	3129.661	3324.428
	best	**1900.43**	5149.529	2912.359	7040.035	2391.62	2961.829	3704.817	2417.094	2698.009	3243.61	3124.039	2948.398	3112.599
	worst	**2138.267**	8109.573	3761.455	12,292.24	2533.313	4035.847	4319.845	3292.78	3057.291	4131.374	3794.717	3425.605	3513.462
	std	137.8644	1253.581	404.4641	2220.67	**64.57045**	478.8141	295.3164	373.1915	170.8534	403.7638	294.9474	232.6045	193.1295
	median	**2022.954**	6747.419	3268.154	9433.143	2476.028	3771.512	4218.79	2936.732	2743.65	3902.147	3582.314	3072.321	3335.826
	rank	**1**	12	6	13	2	9	11	4	3	10	8	5	7
C17-F18	mean	**1830.62**	63,127,397	2,010,961	93,646,402	22,187.1	29,230,906	37,674,620	2,202,290	4,773,593	6,839,233	7,013,146	687,616.1	7,898,737
	best	**1822.239**	50,515,236	260,770.1	42,104,533	3423.859	2,627,757	10,203,343	1,297,604	910,388.1	4,703,720	3,316,065	293,249.4	2,830,469
	worst	**1841.673**	74,439,111	3,683,100	1.3 × 10^8^	33,031.5	83,511,815	68,196,717	3,428,095	9,521,953	9,506,696	13,105,733	1,127,379	18,988,156
	std	**8.365267**	10,614,200	1,781,089	44,344,961	13,284.68	38,179,569	29,459,324	1,046,951	4,615,210	2,086,960	4,583,614	392,927.4	7,665,119
	median	**1829.285**	63,777,620	2,049,987	1.01 × 10^8^	26,146.52	15,392,026	36,149,211	2,041,730	4,331,015	6,573,259	5,815,394	664,918.1	4,888,162
	rank	**1**	12	4	13	2	10	11	5	6	7	8	3	9
C17-F19	mean	**1925.185**	2.27 × 10^9^	216,902	3.2 × 10^9^	2055.895	2.23 × 10^9^	5,705,765	4,273,969	970,130.2	42,276,479	377,244.8	328,564.4	827,342.9
	best	**1924.437**	1.08 × 10^9^	76,334.3	2.16 × 10^9^	2004.909	8,154,682	858,452.1	3,253,382	475,008.9	35,891,244	217,067.2	2737.203	647,243.5
	worst	**1926.121**	3.79 × 10^9^	447,006	3.96 × 10^9^	2081.044	6.51 × 10^9^	13,447,694	5,300,348	1,491,455	53,685,590	826,313.6	820,181.3	1,120,630
	std	**0.812781**	1.17 × 10^9^	165,222.7	8.22 × 10^8^	35.7305	2.99 × 10^9^	5,556,410	858,370.3	436,484.8	8,135,776	307,601.2	400,334.2	229,452.6
	median	**1925.091**	2.11 × 10^9^	172,133.8	3.34 × 10^9^	2068.813	1.2 × 10^9^	4,258,458	4,271,074	957,028.3	39,764,541	232,799.3	245,669.4	770,749.2
	rank	**1**	12	3	13	2	11	9	8	7	10	5	4	6
C17-F20	mean	**2160.172**	3551.92	3081.871	3775.339	2576.451	3223.189	3486.468	3093.513	2546.611	3507.687	3729.636	3101.167	3000.111
	best	**2104.423**	3261.669	2609.066	3531.444	2331.268	2834.542	3227.925	2888.455	2368.84	3394.291	3515.385	2752.085	2935.112
	worst	**2323.891**	3694.096	3519.588	3942.003	2804.926	3426.054	3967.93	3476.746	2745.71	3642.959	3955.542	3240.533	3098.743
	std	112.1118	208.9768	401.3669	178.7862	209.3622	272.2615	345.9497	275.9436	195.8616	112.6999	185.5858	239.6098	**75.4523**
	median	**2106.186**	3625.957	3099.416	3813.954	2584.806	3316.08	3375.009	3004.426	2535.946	3496.748	3723.807	3206.026	2983.294
	rank	**1**	11	5	13	3	8	9	6	2	10	12	7	4
C17-F21	mean	**2314.895**	2882.029	2683.598	2914.293	2427.113	2852.813	2845.191	2531.126	2487.319	2739.199	2756.115	2602.493	2678.824
	best	**2309.045**	2850.955	2580.935	2825.697	2410.875	2762.782	2748.623	2500.882	2440.923	2720.286	2695.833	2541.586	2658.424
	worst	**2329.683**	2913.741	2843.263	2985.933	2446.777	2994.244	2924.65	2561.963	2522.542	2775.614	2788.092	2694.353	2694.385
	std	**10.1546**	31.61239	116.3349	79.13377	18.72946	102.2821	77.95723	31.952	35.48531	26.72565	43.52908	69.35481	18.71277
	median	**2310.426**	2881.71	2655.098	2922.772	2425.4	2827.113	2853.746	2530.829	2492.905	2730.448	2770.268	2587.017	2681.244
	rank	**1**	12	7	13	2	11	10	4	3	8	9	5	6
C17-F22	mean	**3095.169**	13,039.49	9826.909	14,101.28	4984.116	12,002.22	11,940.92	8030.367	7926.015	13,639.71	10,070.5	8665.845	7892.464
	best	**2300**	12,675.96	7743.335	13,787.62	2316.91	11,514.62	11,300.89	6733.052	6917.145	13,106.75	9686.142	7857.368	3745.8
	worst	**5480.678**	13,528.23	11,335.17	14,662.71	7520.843	12,392.91	12,442.24	8996.724	8664.386	14,007.7	10,745.47	9334.598	11,904.18
	std	1633.424	**370.3083**	1769.517	416.5127	2773.424	454.7955	493.6015	974.6443	753.354	395.6171	479.7048	655.9321	4664.853
	median	**2300**	12,976.88	10,114.57	13,977.39	5049.356	12,050.67	12,010.28	8195.845	8061.265	13,722.19	9925.192	8735.706	7959.936
	rank	**1**	11	7	13	2	10	9	5	4	12	8	6	3
C17-F23	mean	**2743.354**	3650.732	3205.096	3714.708	2866.69	3585.901	3588.039	2950.211	2976.3	3196.58	4438.605	3277.074	3264.832
	best	**2729.988**	3582.13	3133.303	3675.628	2856.807	3406.452	3431.629	2915.367	2908.134	3122.479	4273.643	3218.486	3152.818
	worst	**2752.657**	3734.727	3274.637	3747.738	2884.393	3873.109	3674.903	3010.714	3095.707	3254.528	4584.936	3325.963	3382.982
	std	**10.28788**	68.28771	69.37518	31.17598	12.52007	227.7969	111.7276	46.44885	84.38634	56.19525	131.1238	58.53935	96.7561
	median	**2745.387**	3643.036	3206.223	3717.733	2862.78	3532.023	3622.811	2937.381	2950.679	3204.656	4447.92	3281.923	3261.764
	rank	**1**	11	6	12	2	9	10	3	4	5	13	8	7
C17-F24	mean	**2919.043**	4010.998	3421.319	4243.979	3042.809	3837.26	3689.154	3101.868	3155.655	3366.463	4156.081	3378.972	3549.094
	best	**2909.046**	3793.635	3327.519	3829.967	3017.92	3756.028	3595.113	3069.759	3072.317	3300.91	4126.692	3243.297	3515.085
	worst	**2924.412**	4494.655	3578.969	5255.436	3075.399	3956.391	3733.16	3132.026	3264.201	3416.819	4199.726	3512.298	3633.155
	std	**7.008951**	334.167	112.0403	699.6368	26.28215	94.77305	66.02717	27.46018	82.16196	55.82139	34.99998	122.7717	57.76433
	median	**2921.358**	3877.852	3389.395	3945.257	3038.958	3818.31	3714.171	3102.843	3143.051	3374.062	4148.953	3380.147	3524.068
	rank	**1**	11	7	13	2	10	9	3	4	5	12	6	8
C17-F25	mean	**2983.145**	7719.408	3147.649	10,531.67	3054.748	5532.008	3969.86	3043.705	3868.398	4154.663	4073.43	3099.795	3880.771
	best	**2980.235**	6438.194	3123.497	8547.655	3036.952	4583.589	3624.628	3014.213	3703.685	3744.2	3779.306	3061.562	3789.69
	worst	**2991.831**	8529.623	3186.335	11,749.48	3069.982	6433.187	4229.797	3059.654	4040.107	4654.361	4629.915	3141.104	3983.99
	std	**5.947342**	952.1256	27.72478	1545.063	13.98796	816.7002	264.6084	21.23968	180.3098	472.2477	410.9092	41.14992	82.36824
	median	**2980.257**	7954.908	3140.382	10,914.77	3056.028	5555.628	4012.508	3050.476	3864.899	4110.046	3942.249	3098.256	3874.702
	rank	**1**	12	5	13	3	11	8	2	6	10	9	4	7
C17-F26	mean	3776.432	12,485.24	9857.964	13,316.65	**3397.497**	11,251.34	12,257.59	5477.849	6089.917	8800.124	10,340.32	7448.785	8179.371
	best	3748.807	12,285.6	9425.481	12,793.3	**3226.788**	9462.749	11,475.07	5063.435	5759.105	8117.119	10,039.97	6963.818	6593.561
	worst	3793.643	12,649.4	10,292.56	14,122.22	**3644.808**	12,325.1	13,716.48	5703.791	6397.569	9439.729	10,681.34	7925.866	10,230.13
	std	**19.97732**	172.6557	364.1075	593.2476	194.1546	1275.012	1021.872	296.969	342.6607	569.9985	274.3567	443.6232	1775.382
	median	3781.639	12,502.99	9856.909	13,175.53	**3359.195**	11,608.75	11,919.41	5572.084	6101.497	8821.824	10,319.99	7452.727	7946.896
	rank	2	12	8	13	**1**	10	11	3	4	7	9	5	6
C17-F27	mean	**3251.26**	4558.072	3757.226	4717.941	3363.125	4482.345	4271.988	3345.037	3579.706	3740.707	7336.491	3584.334	4258.896
	best	**3227.701**	4286.485	3715.225	4397.922	3268.38	3875.972	3783.542	3308.467	3538.728	3574.105	7117.882	3358.875	4162.671
	worst	**3313.631**	4750.001	3808.977	4953.475	3444.89	4909.919	4755.568	3401.624	3618.589	3892.62	7638.472	3799.69	4378.755
	std	42.83953	208.2423	45.64993	270.5635	75.15709	460.5976	467.4793	40.91249	**40.33389**	144.8357	255.2247	204.2976	94.75696
	median	**3231.854**	4597.9	3752.351	4760.183	3369.614	4571.744	4274.421	3335.029	3580.753	3748.051	7294.805	3589.385	4247.08
	rank	**1**	11	7	12	3	10	9	2	4	6	13	5	8
C17-F28	mean	**3258.849**	7875.995	3541.328	9941.846	3337.808	6631.38	4578.289	3281.782	4224.934	4939.068	4779.577	3776.84	4762.924
	best	**3258.849**	7154.555	3471.646	8859.818	3306.732	5466.288	4063.599	3263.28	3996	4413.751	4728.187	3507.802	4548.706
	worst	**3258.849**	9699.368	3617.366	12,815.67	3375.969	7830.362	4775.276	3297.805	4512.748	5401.291	4880.971	4213.028	4921.505
	std	**0**	1258.68	73.85199	1971.415	34.88036	1231.024	353.5291	17.38953	247.5649	416.8153	71.31116	313.4874	185.5852
	median	**3258.849**	7325.028	3538.151	9045.946	3334.266	6614.436	4737.14	3283.021	4195.493	4970.615	4754.575	3693.265	4790.743
	rank	**1**	12	4	13	3	11	7	2	6	10	9	5	8
C17-F29	mean	**3263.038**	12,013.08	5155.336	16,966.75	3965.861	6335.854	8144.708	4593.855	4625.547	6027.632	7414.208	4596.644	5701.722
	best	**3247.132**	8097.379	5034.352	9211.089	3662.325	5959.515	5651.218	4217.754	4449.501	5261.059	6198.558	4399.641	5441.194
	worst	**3278.787**	16,305.79	5272.736	26,537.5	4170.548	6783.875	10,517.49	5099.099	4881.193	6868.259	9561.805	4667.765	6212.377
	std	**17.92966**	3881.513	100.1932	7927.577	236.6456	351.4129	2058.726	378.9936	203.6804	780.7956	1556.614	134.9533	371.68
	median	**3263.116**	11,824.57	5157.127	16,059.22	4015.285	6300.014	8205.061	4529.282	4585.747	5990.604	6948.234	4659.585	5576.659
	rank	**1**	12	6	13	2	9	11	3	5	8	10	4	7
C17-F30	mean	**623,575.2**	2.73 × 10^9^	18,352,940	4.58 × 10^9^	1,487,669	1.38 × 10^9^	1.32 × 10^8^	58,942,891	1.16 × 10^8^	2.51 × 10^8^	1.54 × 10^8^	4,120,584	48,887,890
	best	**582,411.6**	2.11 × 10^9^	11,254,579	2.81 × 10^9^	1,154,970	1.7 × 10^8^	89,570,536	53,274,378	56,426,470	1.75 × 10^8^	1.18 × 10^8^	2,907,481	39,468,838
	worst	**655,637.4**	3.71 × 10^9^	25,121,412	7.19 × 10^9^	2,358,780	2.81 × 10^9^	1.83 × 10^8^	67,789,140	1.73 × 10^8^	3.18 × 10^8^	2.02 × 10^8^	5,692,794	68,578,838
	std	**33,550.87**	7.17 × 10^8^	6,997,250	1.94 × 10^9^	599,306	1.4 × 10^9^	48,032,660	6,469,130	60,378,267	61,457,449	36,165,698	1,416,298	13,842,435
	median	**628,125.9**	2.56 × 10^9^	18,517,886	4.16 × 10^9^	1,218,463	1.28 × 10^9^	1.29 × 10^8^	57,354,023	1.18 × 10^8^	2.56 × 10^8^	1.48 × 10^8^	3,941,030	43,751,942
	rank	**1**	12	4	13	2	11	8	6	7	10	9	3	5
Sum rank		**30**	335	166	367	63	294	269	112	144	248	254	150	207
Mean rank		**1.034483**	11.55172	5.724138	12.65517	2.172414	10.13793	9.275862	3.862069	4.965517	8.551724	8.758621	5.172414	7.137931
Total rank		**1**	12	6	13	2	11	10	3	4	8	9	5	7

**Table 5:** Optimization results of CEC 2017 test suite (dimension = 100); background color has been used in order to make the table more reader-friendly and to separate the results of benchmark functions from each other; The best results are specified using bold.

		POA	WSO	AVOA	RSA	MPA	TSA	WOA	MVO	GWO	TLBO	GSA	PSO	GA
C17-F1	mean	**100**	1.39 × 10^11^	3.19 × 10^9^	1.94 × 10^11^	4.33 × 10^8^	1.05 × 10^11^	5.23 × 10^10^	54,852,229	4.76 × 10^10^	7.6 × 10^10^	1.14 × 10^11^	1.67 × 10^10^	4.68 × 10^10^
	best	**100**	1.36 × 10^11^	1.55 × 10^9^	1.91 × 10^11^	3.28 × 10^8^	9.25 × 10^10^	4.94 × 10^10^	45,706,387	4.13 × 10^10^	7.24 × 10^10^	1.04 × 10^11^	1.12 × 10^10^	4.43 × 10^10^
	worst	**100**	1.43 × 10^11^	4.59 × 10^9^	1.96 × 10^11^	5.47 × 10^8^	1.17 × 10^11^	5.85 × 10^10^	64,235,296	5.39 × 10^10^	8.38 × 10^10^	1.21 × 10^11^	2.27 × 10^10^	5.28 × 10^10^
	std	**1.19 × 10^−14^**	2.93 × 10^9^	1.28 × 10^9^	2.3 × 10^9^	1.09 × 10^8^	1.06 × 10^10^	4.32 × 10^9^	9,293,959	6.15 × 10^9^	5.41 × 10^9^	7.44 × 10^9^	6.48 × 10^9^	4.2 × 10^9^
	median	**100**	1.39 × 10^11^	3.31 × 10^9^	1.95 × 10^11^	4.29 × 10^8^	1.05 × 10^11^	5.06 × 10^10^	54,733,616	4.77 × 10^10^	7.4 × 10^10^	1.14 × 10^11^	1.64 × 10^10^	4.5 × 10^10^
	rank	**1**	12	4	13	3	10	8	2	7	9	11	5	6
C17-F3	mean	**300**	356,822.5	272,039.4	268,843.8	131,575.1	302,970	657,547.9	388,370.4	306,828.3	246,796	286,153.2	450,679.2	481,038.8
	best	**300**	325,159.2	265,681.1	259,332.9	100,728.8	242,793.7	575,512.9	322,609.5	280,769.3	231,497.7	264,862.3	341,428.2	461,303.6
	worst	**300**	373,192.5	278,132.7	274,437.1	159,198.2	345,991.9	761,479.5	464,924.1	336,002.8	261,138.9	313,191.9	632,641.2	496,677.8
	std	**0**	23,069.08	5405.063	7264.194	26,086.31	44888	82,337.72	74,421.55	30,350.08	12,441.73	20,597.91	138,498.8	16,046.35
	median	**300**	364,469	272,171.8	270,802.5	133,186.8	311,547.2	646,599.6	382,974	305,270.6	247,273.7	283,279.4	414,323.8	483,086.9
	rank	**1**	9	5	4	2	7	13	10	8	3	6	11	12
C17-F4	mean	**602.1722**	37,200.13	1395.351	62,656.05	949.0996	13,432.06	9212.415	733.9504	3820.981	9046.233	28,507.72	2159.921	7767.034
	best	**592.0676**	34,244.38	1188.769	56,805.13	853.8108	8822.324	7863.571	686.5776	2959.035	8625.057	22,690.11	1346.902	7344.033
	worst	**612.2769**	40,775.07	1525.893	69,800.05	1048.065	17,828.22	10,104.17	782.7016	5694.419	9775.514	32,247.61	2697.897	8247.69
	std	**11.98393**	2884.073	157.7992	5534.342	96.64646	3822.612	978.9794	41.15435	1294.375	562.1131	4740.337	598.0332	430.9479
	median	**602.1722**	36,890.53	1433.37	62,009.52	947.2614	13,538.85	9440.961	733.2611	3315.236	8892.181	29,546.57	2297.443	7738.206
	rank	**1**	12	4	13	3	10	9	2	6	8	11	5	7
C17-F5	mean	**512.9345**	1713.456	1158.356	1688.389	1085.416	1839.181	1587.534	1094.013	1050.695	1616.155	1176.387	1240.032	1378.034
	best	**510.9445**	1698.059	1148.207	1659.418	980.744	1818.63	1507.616	1004.061	1003.78	1593.486	1148.27	1158.131	1257.35
	worst	**514.9244**	1722.799	1165.849	1716.686	1156.185	1863.108	1712.415	1151.854	1090.71	1640.148	1202.516	1381.78	1450.591
	std	**1.865752**	11.03134	7.635601	29.64844	86.76592	21.19345	91.26585	68.35644	38.92925	19.58371	29.03695	107.9789	89.24287
	median	**512.9345**	1716.483	1159.684	1688.726	1102.367	1837.493	1565.052	1110.068	1054.144	1615.493	1177.382	1210.109	1402.098
	rank	**1**	12	5	11	3	13	9	4	2	10	6	7	8
C17-F6	mean	**600**	686.2561	650.1087	684.8479	630.2372	689.9604	684.22	660.4596	632.5629	665.8413	651.8302	649.7509	651.0804
	best	**600**	684.0572	646.8065	680.8364	627.1287	679.9345	676.264	654.9711	628.4932	658.6757	649.7166	643.883	645.0896
	worst	**600**	688.314	653.57	687.2264	635.5005	696.8682	698.2933	665.5673	637.7053	670.1556	655.2582	654.5349	655.6
	std	**0**	1.973412	2.863424	2.900847	4.076006	8.394353	10.15573	4.674826	4.103813	5.638744	2.510844	5.196738	5.435079
	median	**600**	686.3265	650.0291	685.6644	629.1598	691.5195	681.1614	660.6501	632.0266	667.2669	651.173	650.2929	651.8159
	rank	**1**	12	5	11	2	13	10	8	3	9	7	4	6
C17-F7	mean	**811.392**	3078.549	2647.876	3172.9	1642.885	2936.941	3055.083	1776.654	1789.226	2661.299	2681.923	2157.269	2236.865
	best	**810.0205**	3007.362	2517.822	3098.601	1595.363	2790.616	2956.24	1643.858	1635.004	2543.213	2575.234	1939.203	2154.734
	worst	**813.1726**	3162.245	2757.1	3235.912	1709.012	3075.254	3198.987	1877.404	1903.283	2758.246	2858.635	2254.415	2416.55
	std	**1.500732**	65.36608	123.0115	60.62822	50.50046	131.9066	114.421	99.82659	115.2611	91.24165	126.9898	153.3934	124.6292
	median	**811.1874**	3072.294	2658.291	3178.543	1633.582	2940.946	3032.553	1792.676	1809.308	2671.868	2646.912	2217.73	2188.088
	rank	**1**	12	7	13	2	10	11	3	4	8	9	5	6
C17-F8	mean	**812.437**	2111.514	1558.999	2155.866	1311.287	2093.096	2028.263	1330.475	1380.248	1975.447	1630.458	1533.499	1796.9
	best	**808.9546**	2070.756	1514.017	2136.282	1172.108	2037.043	1865.023	1206.239	1294.809	1922.602	1563.68	1500.057	1755.117
	worst	**816.9143**	2160.449	1581.305	2168.607	1397.316	2164.109	2153.686	1478.289	1494.139	2018.868	1736.527	1610.497	1838.762
	std	**3.490503**	39.46893	31.82517	14.21747	101.6924	62.6837	151.6583	115.3078	92.24407	42.9253	79.30347	53.02921	36.57804
	median	**811.9395**	2107.427	1570.336	2159.287	1337.862	2085.616	2047.173	1318.687	1366.021	1980.159	1610.813	1511.721	1796.86
	rank	**1**	12	6	13	2	11	10	3	4	9	7	5	8
C17-F9	mean	**900**	72673	21,463.42	62,321.68	18,200.53	97,087.11	61,930.12	47,807.54	29,078.92	60,047.03	19,113.92	26,597.15	37,150.16
	best	**900**	64,890.1	17,889.47	60,247.62	16,949.52	79,614.15	48,186.59	40,332.69	18,044.02	57,514.84	17,799.99	22,511.34	33,658.16
	worst	**900**	83,934.4	24,142.23	64,020.16	18,766.82	121,069	78,018.06	54,357.12	39,496.25	61,393.32	20,131.39	29,609.34	41,831.24
	std	**9.53 × 10^−14^**	8439.843	2676.226	1688.451	863.4577	17,876.94	15,298.97	5930.986	10,756.32	1817.986	1001.368	3241.433	3525.3
	median	**900**	70,933.74	21,910.99	62,509.46	18,542.9	93,832.65	60,757.92	48,270.17	29,387.71	60,639.98	19,262.15	27,133.95	36,555.61
	rank	**1**	12	4	11	2	13	10	8	6	9	3	5	7
C17-F10	mean	**11,023.04**	26,654.96	15,020.3	27,732.34	13,324.39	25,915.94	25,070.06	15,849.77	14,388.1	27,740.18	16,039.68	15,915.96	23,258.07
	best	**9625.608**	26,353.3	12,995.83	27,038.62	12,781.35	25,353.33	24,414.69	15,359.08	13,258.65	26,507.18	14,606.66	14,564.86	22,622.29
	worst	**11,858.81**	27,030.57	16,910.95	28,251.05	14,141.58	26,715.13	26,344.54	16,388.95	14,902.23	28,667.37	16,907.43	16,756.64	23,837.44
	std	995.114	**320.0939**	1798.552	577.2643	634.1048	654.8937	901.4097	496.5617	784.6955	931.1423	1107.631	971.114	511.2961
	median	**11,303.87**	26,617.99	15,087.21	27,819.85	13,187.32	25,797.65	24,760.5	15,825.52	14,695.77	27,893.09	16,322.32	16,171.17	23,286.27
	rank	**1**	11	4	12	2	10	9	5	3	13	7	6	8
C17-F11	mean	**1162.329**	134,612.3	52,588.01	168,880.7	4126.681	53,605.52	170,451.2	3961.419	71,430.16	58,834.42	141,339.7	42,727.78	113,994.6
	best	**1139.568**	104,512.5	47,266.62	129,243.6	3290.22	24,535.81	99,263.93	3471.134	59,361.4	49,651.74	117,805.8	19,535.8	87,032.77
	worst	**1220.662**	156,634.3	62,800.07	240,550.6	4901.723	76,617.73	274,712.4	4194.313	80,462.48	74,953.97	164,872.9	87,137.65	157,108.2
	std	**40.09338**	23,016.42	7379.838	51,504.53	711.6997	22,173.17	83,830.02	340.274	9275.106	11,393.93	19,966.49	31,050.73	31,530.95
	median	**1144.542**	138,651.1	50,142.67	152,864.4	4157.391	56,634.27	153,914.3	4090.115	72,948.37	55,365.98	141,340.1	32,118.83	105,918.8
	rank	**1**	10	5	12	3	6	13	2	8	7	11	4	9
C17-F12	mean	**5974.805**	8.62 × 10^10^	5.38 × 10^8^	1.4 × 10^11^	2.13 × 10^8^	4.64 × 10^10^	1.08 × 10^10^	2.72 × 10^8^	9.35 × 10^9^	1.79 × 10^10^	5.46 × 10^10^	8.25 × 10^9^	1.01 × 10^10^
	best	**5383.905**	6.13 × 10^10^	2.85 × 10^8^	1.05 × 10^11^	1.19 × 10^8^	2.38 × 10^10^	8.75 × 10^9^	1.73 × 10^8^	6.48 × 10^9^	1.41 × 10^10^	4.74 × 10^10^	1.07 × 10^9^	9.19 × 10^9^
	worst	**6570.199**	9.61 × 10^10^	8.59 × 10^8^	1.63 × 10^11^	2.55 × 10^8^	7.7 × 10^10^	1.23 × 10^10^	4.27 × 10^8^	1.11 × 10^10^	2.47 × 10^10^	6.42 × 10^10^	1.57 × 10^10^	1.19 × 10^10^
	std	**507.8693**	1.71 × 10^10^	2.54 × 10^8^	2.73 × 10^10^	65,009,208	2.28 × 10^10^	1.54 × 10^9^	1.15 × 10^8^	2.06 × 10^9^	4.98 × 10^9^	7.21 × 10^9^	6.83 × 10^9^	1.27 × 10^9^
	median	**5972.559**	9.37 × 10^10^	5.04 × 10^8^	1.47 × 10^11^	2.39 × 10^8^	4.25 × 10^10^	1.1 × 10^10^	2.44 × 10^8^	9.9 × 10^9^	1.65 × 10^10^	5.34 × 10^10^	8.12 × 10^9^	9.62 × 10^9^
	rank	**1**	12	4	13	2	10	8	3	6	9	11	5	7
C17-F13	mean	**1407.28**	2.28 × 10^10^	80,574.27	3.49 × 10^10^	79,471.01	1.75 × 10^10^	4.27 × 10^8^	289,792.7	7.74 × 10^8^	2.3 × 10^9^	7.14 × 10^9^	1.44 × 10^9^	1.43 × 10^8^
	best	**1371.145**	1.98 × 10^10^	56,979.61	2.7 × 10^10^	34,182.78	1.24 × 10^10^	3.04 × 10^8^	255,398.6	66,792,621	1.59 × 10^9^	4.39 × 10^9^	1.59 × 10^8^	1.12 × 10^8^
	worst	**1439.935**	2.53 × 10^10^	109,768.5	3.95 × 10^10^	197,022.9	2.09 × 10^10^	5.78 × 10^8^	337,817.3	2.05 × 10^9^	2.79 × 10^9^	9.17 × 10^9^	2.61 × 10^9^	1.72 × 10^8^
	std	**35.69163**	2.91 × 10^9^	22,990.78	5.96 × 10^9^	80968	3.7 × 10^9^	1.45 × 10^8^	37,057.41	9.39 × 10^8^	5.59 × 10^8^	2.05 × 10^9^	1.24 × 10^9^	31,895,575
	median	**1409.02**	2.3 × 10^10^	77,774.48	3.65 × 10^10^	43,339.18	1.83 × 10^10^	4.14 × 10^8^	282,977.3	4.92 × 10^8^	2.42 × 10^9^	7.5 × 10^9^	1.5 × 10^9^	1.44 × 10^8^
	rank	**1**	12	3	13	2	11	6	4	7	9	10	8	5
C17-F14	mean	**1467.509**	37,197,454	5,466,685	65,255,522	74,687.09	7,286,846	11,918,194	2,485,352	7,878,524	11,393,244	9,418,373	667,883.9	8,603,616
	best	**1458.803**	32,123,384	3,315,151	59,516,459	21,503.76	3,309,974	6,860,812	750,232.9	4,983,390	8,488,751	7,259,554	317,528.2	4,814,013
	worst	**1472.733**	42,492,312	9,074,791	71,434,249	158,404.6	14,217,196	16,291,558	3,420,974	11,810,971	14,559,371	14,124,953	1,386,468	12,674,056
	std	**6.209197**	4,677,964	2,605,972	5,879,918	62,965.71	4,935,970	3,982,571	1,222,873	3,073,855	3,260,247	3,259,370	499,763.2	3,360,478
	median	**1469.25**	37,087,060	4,738,399	65,035,690	59,419.98	5,810,108	12,260,204	2,885,101	7,359,867	11,262,427	8,144,493	483,769.8	8,463,198
	rank	**1**	12	5	13	2	6	11	4	7	10	9	3	8
C17-F15	mean	**1609.893**	1.26 × 10^10^	69,370.46	1.93 × 10^10^	46,148.1	9.88 × 10^9^	57,422,101	103,903	4.11 × 10^8^	9.76 × 10^8^	1.02 × 10^9^	2.73 × 10^8^	10,394,502
	best	**1551.154**	1.17 × 10^10^	56,780.13	1.38 × 10^10^	13,517.63	2.05 × 10^8^	31,981,638	71,184.3	26,937,678	3.26 × 10^8^	4.07 × 10^8^	50,630.11	6,702,420
	worst	**1652.294**	1.42 × 10^10^	87,025.88	2.4 × 10^10^	69,938.59	1.85 × 10^10^	1.1 × 10^8^	152,770.1	1.23 × 10^9^	2.08 × 10^9^	1.3 × 10^9^	1.08 × 10^9^	17,711,015
	std	**45.3586**	1.12 × 10^9^	14,851.33	5.23 × 10^9^	24,462.7	8.16 × 10^9^	36,744,160	36,893.9	5.72 × 10^8^	7.92 × 10^8^	4.25 × 10^8^	5.52 × 10^8^	5,136,228
	median	**1618.063**	1.23 × 10^10^	66,837.92	1.97 × 10^10^	50,568.08	1.04 × 10^10^	43,686,519	95,828.73	1.93 × 10^8^	7.47 × 10^8^	1.18 × 10^9^	7,071,707	8,582,287
	rank	**1**	12	3	13	2	11	6	4	8	9	10	7	5
C17-F16	mean	**2711.795**	16,012.75	6339.766	19,049.54	5020.833	12,428.05	13,797.85	5899.538	5494.114	9913.052	9553.895	5807.791	9132.825
	best	**2171.69**	15,028.6	5463.708	15,158.15	4904.968	10,251.41	11,324.27	5278.217	4983.364	9463.009	8315.094	5671.273	8374.525
	worst	**3397.326**	16,418.68	6926.982	21,180.98	5123.19	14,905.49	15,213.89	6292.986	5983.629	10,735.61	10,910.6	5919.126	9778.948
	std	523.7732	679.046	642.8286	2818.137	118.6712	1964.904	1809.969	491.0011	564.8153	614.6226	1208.589	**105.6406**	647.8327
	median	**2639.081**	16,301.87	6484.187	19,929.51	5027.588	12,277.66	14,326.62	6013.474	5504.731	9726.794	9494.942	5820.383	9188.914
	rank	**1**	12	6	13	2	10	11	5	3	9	8	4	7
C17-F17	mean	**2716.564**	3,460,690	5278.977	6,807,697	4297.894	179,834.7	14,457.77	4560.73	5000.734	7657.1	38,547.08	5486.593	6354.464
	best	**2275.021**	1,014,586	5040.925	1,845,539	4049.324	8854.736	9018.615	4149.113	4121.38	7508	25,526.42	5266.846	6299.009
	worst	**3429.127**	7,873,139	5666.053	15,664,206	4526.101	476,948.7	24,245.76	4922.177	6445.983	7893.557	62,324.99	5629.111	6460.004
	std	528.3898	3,320,761	301.2767	6,677,440	227.8255	210,069.5	7045.023	392.0188	1070.156	175.195	16,751.95	158.8681	**73.75885**
	median	**2581.054**	2,477,518	5204.466	4,860,521	4308.075	116,767.6	12,283.34	4585.815	4717.786	7613.422	33,168.45	5525.207	6329.422
	rank	**1**	12	5	13	2	11	9	3	4	8	10	6	7
C17-F18	mean	**1903.746**	47,894,555	2,310,227	84,518,490	190,659.1	12,221,836	9,843,795	4,025,843	8,988,758	13,289,966	9,641,892	5,279,389	4,952,111
	best	**1881.15**	21,698,211	1,148,961	32,805,132	133,021.7	4,578,030	7,321,990	2,981,556	2,830,828	9,793,146	4,445,340	3,260,604	3,970,706
	worst	**1919.921**	86,612,495	3,652,415	1.55 × 10^8^	343,343.4	24,974,816	11,661,348	6,762,303	14,525,423	18,785,306	21,434,676	7,605,804	7,169,003
	std	**19.90425**	28,481,148	1,164,994	52,706,461	104,871	9,439,239	2,031,181	1,881,089	4,942,116	3,967,275	8,225,806	2,073,772	1,546,019
	median	**1906.955**	41,633,757	2,219,766	75,362,668	143,135.6	9,667,250	10,195,921	3,179,757	9,299,390	12,290,706	6,343,776	5,125,574	4,334,367
	rank	**1**	12	3	13	2	10	9	4	7	11	8	6	5
C17-F19	mean	**1972.839**	1.04 × 10^10^	2,362,491	1.83 × 10^10^	230,108.6	4.14 × 10^9^	1.1 × 10^8^	13,654,870	2.96 × 10^8^	5.49 × 10^8^	1.3 × 10^9^	2.21 × 10^8^	10,503,303
	best	**1967.139**	9.2 × 10^9^	904,673.4	1.34 × 10^10^	48,684.43	1.84 × 10^9^	43,630,380	7,964,383	2,348,641	2.38 × 10^8^	2.33 × 10^8^	36,773,561	5,362,045
	worst	**1977.869**	1.23 × 10^10^	4,348,722	2.28 × 10^10^	389,547.4	8.23 × 10^9^	1.85 × 10^8^	21,704,035	8.9 × 10^8^	1.26 × 10^9^	2.45 × 10^9^	4.79 × 10^8^	18,995,339
	std	**4.659759**	1.43 × 10^9^	1,495,119	4 × 10^9^	145,313.2	2.9 × 10^9^	67,427,821	6,966,481	4.26 × 10^8^	4.94 × 10^8^	1.13 × 10^9^	2.2 × 10^8^	6,213,630
	median	**1973.174**	1.01 × 10^10^	2,098,285	1.86 × 10^10^	241,101.2	3.25 × 10^9^	1.05 × 10^8^	12,475,530	1.46 × 10^8^	3.48 × 10^8^	1.25 × 10^9^	1.85 × 10^8^	8,827,915
	rank	**1**	12	3	13	2	11	6	5	8	9	10	7	4
C17-F20	mean	**3192.04**	6567.794	5653.156	6776.389	4267.781	6355.055	6365.416	5352.871	5569.311	6533.95	5772.471	4990.757	5732.389
	best	**2806.762**	6354.057	5417.394	6712.36	4167.712	5784.031	6063.448	5126.343	4577.831	5811.094	5463.899	4325.736	5172.25
	worst	**3662.121**	6787.248	5871.457	6910.434	4349.534	7064.472	6720.677	5744.821	6391.821	6826.52	5934.124	5777.557	6089.943
	std	451.2632	195.8611	240.0771	93.26987	**77.59644**	573.7388	296.3053	281.3659	910.8455	496.684	216.9234	643.6342	451.9964
	median	**3149.639**	6564.936	5661.887	6741.381	4276.94	6285.858	6338.769	5270.159	5653.796	6749.093	5845.931	4929.869	5833.682
	rank	**1**	12	6	13	2	9	10	4	5	11	8	3	7
C17-F21	mean	**2342.155**	3936.255	3428.272	4037.847	2744.718	3800.888	3885.327	3076.144	2861.886	3461.987	4286.766	3358.879	3224.172
	best	**2338.689**	3897.727	3253.086	3974.827	2708.006	3681.123	3637.319	3018.663	2793.484	3329.061	3827.764	3203.068	3194.14
	worst	**2346.015**	3994.366	3542.899	4085.116	2773.289	3882.291	4076.884	3183.716	2907.116	3613.682	4655.793	3653.096	3265.66
	std	**3.460098**	47.67605	128.2378	49.14714	28.36417	101.2622	202.301	75.94341	49.60032	124.534	354.7142	209.5381	31.7634
	median	**2341.959**	3926.465	3458.551	4045.723	2748.788	3820.069	3913.553	3051.099	2873.472	3452.602	4331.754	3289.676	3218.445
	rank	**1**	11	7	12	2	9	10	4	3	8	13	6	5
C17-F22	mean	**11739**	28,172.59	18,735.12	29,538.13	17,455.15	27,345.2	25,999.83	16,295.9	21,263.1	29,437.21	19,478.09	20,105.81	25,721.35
	best	**11,119.08**	27,395.55	17,607.65	29,162.03	16,460.94	26,279.36	24,653.63	15,563.07	17,435.82	28,520.7	18,881.33	18,940.91	24,806.45
	worst	**12,601.6**	28,629.67	20,191.76	30,067.56	18,790.81	28,390.93	27,133.25	16,899.95	30,390.26	29,867.78	19,882.71	21,371.61	26,403.85
	std	670.4039	589.6365	1219.607	**397.6551**	1012.878	888.746	1118.426	683.2184	6344.73	641.5669	435.4176	1030.321	823.7796
	median	**11,617.67**	28,332.57	18,570.54	29,461.46	17,284.43	27,355.26	26,106.21	16,360.29	18,613.17	29,680.17	19,574.16	20,055.37	25,837.55
	rank	**1**	11	4	13	3	10	9	2	7	12	5	6	8
C17-F23	mean	**2877.697**	4909.274	3897.417	4911.066	3224.427	5009.228	4754.862	3379.513	3490.336	3981.02	7024.793	4523.727	4024.355
	best	**2872.107**	4698.658	3829.733	4686.8	3212.144	4375.185	4635.595	3302.136	3462.519	3935.695	6528.447	4088.171	3968.4
	worst	**2884.013**	5155.534	3968.806	5086.335	3250.782	5868.487	4874.469	3478.908	3528.331	4046.2	7376.676	4755.132	4077.859
	std	**5.357202**	210.0399	66.98634	170.0295	18.22406	686.4955	117.7478	76.61245	30.8675	47.9752	393.8005	308.3436	61.38807
	median	**2877.334**	4891.451	3895.564	4935.565	3217.391	4896.619	4754.692	3368.504	3485.247	3971.092	7097.025	4625.801	4025.582
	rank	**1**	10	5	11	2	12	9	3	4	6	13	8	7
C17-F24	mean	**3327.407**	7654.069	5028.818	9295.819	3650.5	6109.359	5866.757	3860.667	4131.949	4512.935	9557.407	5518.107	5031.28
	best	**3295.518**	6080.871	4849.854	6396.632	3611.852	5694.234	5511.285	3798.2	3936.704	4319.754	9006.742	5206.928	4959.558
	worst	**3357.991**	8722.239	5178.953	11,219.82	3708.311	6378.761	6406.139	3951.043	4307.423	4701.792	10,990.06	5915.538	5169.385
	std	**30.42243**	1296.2	149.2755	2398.2	47.49002	300.5585	399.0602	73.00364	199.5073	160.6953	982.4706	324.0293	98.16265
	median	**3328.059**	7906.583	5043.231	9783.41	3640.918	6182.221	5774.802	3846.713	4141.834	4515.097	9116.414	5474.982	4998.089
	rank	**1**	11	6	12	2	10	9	3	4	5	13	8	7
C17-F25	mean	**3185.232**	13,234.48	3979.524	18,274.73	3601.961	9249.247	6611.679	3371.093	5886.412	7946.071	9713.328	3980.408	7098.013
	best	**3137.371**	12,601.16	3665.163	16,991.17	3447.531	8695.57	6085.761	3309.86	5764.028	6909.91	8992.442	3756.703	6495.826
	worst	**3261.571**	14,707.24	4270.313	21,165.62	3717.086	9610.909	6926.438	3431.056	6217.517	9331.78	10,981.67	4331.937	7700.584
	std	61.52949	1018.704	255.7634	2022.54	115.5731	424.0722	391.3741	**51.16903**	227.2854	1137.891	902.3143	285.8758	640.5351
	median	**3170.992**	12,814.75	3991.31	17,471.07	3621.612	9345.255	6717.259	3371.727	5782.052	7771.298	9439.599	3916.495	7097.821
	rank	**1**	12	4	13	3	10	7	2	6	9	11	5	8
C17-F26	mean	**5757.621**	33,814.21	21,454.77	38,754.91	10,644.44	28,664.88	29,186.46	10,818.08	14,996.27	20,816.13	29,105.02	18190	20,097.35
	best	**5645.905**	33,338.22	19,072.76	36,608.49	10,053.37	27,618.72	26,275.7	9665.832	13,418.35	17,195.2	27,945.26	16,373.82	18,751.66
	worst	**5844.642**	34,231.54	23,904.72	40,086.1	11,279.01	29,309.07	31,664.53	12,812.66	16,341.08	25,395.27	30,626.7	19,856.37	21,014.33
	std	**86.19253**	384.2725	2124.891	1711.43	626.4884	750.0704	2731.102	1414.348	1266.478	3492.437	1155.517	1506.606	1001.112
	median	**5769.969**	33,843.54	21,420.8	39,162.52	10,622.68	28,865.87	29,402.8	10,396.92	15,112.83	20,337.02	28,924.06	18,264.9	20,311.69
	rank	**1**	12	8	13	2	9	11	3	4	7	10	5	6
C17-F27	mean	**3309.493**	8327.753	4022.239	10,795.84	3497.578	6058.864	5560.881	3572.454	3954.812	4160.881	12,269.99	3948.446	5126.891
	best	**3278.01**	7097.49	3875.389	8220.487	3469.565	5810.983	4968.438	3536.2	3809.989	3928.691	11,978.11	3778.598	4908.649
	worst	**3344.5**	9574.168	4263.384	13,478.56	3524.275	6368.227	6219.759	3649.169	4072.225	4543.659	12,503.97	4115.756	5453.529
	std	29.13307	1382.852	172.1394	2911.656	**22.95773**	248.8617	690.9647	53.9399	132.5784	279.9852	242.9169	193.7832	239.7051
	median	**3307.732**	8319.677	3975.091	10,742.15	3498.236	6028.123	5527.664	3552.225	3968.516	4085.586	12,298.95	3949.714	5072.694
	rank	**1**	11	6	12	2	10	9	3	5	7	13	4	8
C17-F28	mean	**3322.242**	17,991.2	4478.19	24,108.64	3697.249	13,651.33	9207.387	3436.49	8286.21	9873.556	16,237.66	6929.717	10,135.48
	best	**3318.742**	16,786.97	4225.378	21,649.57	3592.713	10,824.52	7941.293	3366.715	7097.487	7824.721	14,079.56	4870.127	9275.217
	worst	**3327.816**	20,221.49	4664.684	27,186.66	3771.72	15,794.61	10,035.95	3504.902	9983.366	11,672.86	17,865.46	10,422.95	11,089.27
	std	**4.500714**	1604.85	191.0708	2384.987	77.38139	2446.604	916.0249	58.38879	1250.086	1842.249	1626.437	2598.878	997.1342
	median	**3321.205**	17,478.16	4511.349	23,799.16	3712.281	13,993.1	9426.153	3437.172	8031.994	9998.32	16,502.81	6212.894	10,088.71
	rank	**1**	12	4	13	3	10	7	2	6	8	11	5	9
C17-F29	mean	**4450.696**	153,525.4	8751.92	291,582.6	6470.85	16,101.64	14,518.18	7969.069	7654.422	11,086.21	21,508.36	7938.949	10,592.56
	best	**4169.151**	87,746.73	7664.862	156,769.3	5742.449	12,568.33	12,184.87	7242.033	7468.228	10,329.34	17,877.27	7368.964	10,404.47
	worst	**4829.521**	209,282.3	9350.469	404,565.4	7122.632	20,193.8	16,519.68	8491.258	7895.494	11,599.65	27,959.25	8649.222	10,989.46
	std	289.9914	53,145.28	764.3004	108,453.6	584.5761	3272.876	2201.057	551.5885	**183.939**	554.7787	4827.237	610.5231	282.6706
	median	**4402.056**	158,536.3	8996.175	302,497.8	6509.159	15,822.21	14,684.08	8071.493	7626.983	11,207.92	20,098.45	7868.806	10,488.15
	rank	**1**	12	6	13	2	10	9	5	3	8	11	4	7
C17-F30	mean	**5407.166**	1.93 × 10^10^	23,035,407	3.13 × 10^10^	3,901,472	1.11 × 10^10^	1.25 × 10^9^	85,519,419	1.53 × 10^9^	3.14 × 10^9^	6.1 × 10^9^	5.03 × 10^8^	5.53 × 10^8^
	best	**5337.48**	1.69 × 10^10^	13,126,945	2.93 × 10^10^	1,739,030	6.77 × 10^9^	1.02 × 10^9^	52,622,040	6.27 × 10^8^	1.18 × 10^9^	4.36 × 10^9^	1.22 × 10^8^	4.61 × 10^8^
	worst	**5557.155**	2.09 × 10^10^	40,507,493	3.39 × 10^10^	6,370,142	1.38 × 10^10^	1.69 × 10^9^	1.05 × 10^8^	2 × 10^9^	5.83 × 10^9^	7.39 × 10^9^	1.56 × 10^9^	5.93 × 10^8^
	std	**103.8976**	1.74 × 10^9^	12,590,648	2.04 × 10^9^	2,198,236	3.15 × 10^9^	3.09 × 10^8^	24,056,210	6.32 × 10^8^	2.4 × 10^9^	1.32 × 10^9^	7.23 × 10^8^	63,276,132
	median	**5367.014**	1.96 × 10^10^	19,253,594	3.11 × 10^10^	3,748,358	1.2 × 10^10^	1.14 × 10^9^	92,179,183	1.74 × 10^9^	2.78 × 10^9^	6.34 × 10^9^	1.65 × 10^8^	5.79 × 10^8^
	rank	**1**	12	3	13	2	11	7	4	8	9	10	5	6
Sum rank		**29**	336	140	355	65	293	265	114	156	249	272	162	203
Mean rank		**1**	11.586207	4.8275862	12.241379	2.2413793	10.103448	9.137931	3.9310345	5.3793103	8.5862069	9.3793103	5.5862069	7
Total rank		**1**	12	4	13	2	11	9	3	5	8	10	6	7

**Table 6:** Wilcoxon rank sum test results.

Compared Algorithm	Objective Function Type			
	CEC 2017			
	D = 10	D = 30	D = 50	D = 100
POA vs. WSO	1.28 × 10^−21^	1.28 × 10^−21^	1.28 × 10^−21^	1.28 × 10^−21^
POA vs. AVOA	2.46 × 10^−19^	1.98 × 10^−21^	1.28 × 10^−21^	1.28 × 10^−21^
POA vs. RSA	1.28 × 10^−21^	1.28 × 10^−21^	1.28 × 10^−21^	1.28 × 10^−21^
POA vs. MPA	1.31 × 10^−18^	1.02 × 10^−16^	4.33 × 10^−18^	1.28 × 10^−21^
POA vs. TSA	6.20 × 10^−21^	1.28 × 10^−21^	1.28 × 10^−21^	1.28 × 10^−21^
POA vs. WOA	6.20 × 10^−21^	1.28 × 10^−21^	1.28 × 10^−21^	1.28 × 10^−21^
POA vs. MVO	5.89 × 10^−19^	1.39 × 10^−21^	1.28 × 10^−21^	1.28 × 10^−21^
POA vs. GWO	3.41 × 10^−21^	1.28 × 10^−21^	1.28 × 10^−21^	1.28 × 10^−21^
POA vs. TLBO	2.41 × 10^−21^	1.28 × 10^−21^	1.28 × 10^−21^	1.28 × 10^−21^
POA vs. GSA	1.05 × 10^−18^	1.32 × 10^−21^	1.28 × 10^−21^	1.28 × 10^−21^
POA vs. PSO	1.01 × 10^−19^	1.54 × 10^−21^	1.28 × 10^−21^	1.28 × 10^−21^
POA vs. GA	1.76 × 10^−19^	1.28 × 10^−21^	1.28 × 10^−21^	1.28 × 10^−21^

**Table 7:** Optimization results of the CEC 2011 test suite; background color has been used in order to make the table more reader-friendly and to separate the results of benchmark functions from each other; The best results are specified using bold.

		POA	WSO	AVOA	RSA	MPA	TSA	WOA	MVO	GWO	TLBO	GSA	PSO	GA
C11-F1	mean	**5.920103**	16.29617	12.12715	20.06805	7.372872	16.94094	12.38434	13.04843	10.27841	16.96911	19.8221	16.53429	21.31513
	best	**2 × 10^−10^**	13.50111	7.791703	17.72143	0.326893	15.41917	7.235374	11.08197	0.980679	16.26314	17.25998	9.2021	19.60384
	worst	**12.30606**	19.44839	16.24841	22.93654	12.64011	18.86907	16.64328	15.36547	15.30159	17.55835	21.86564	22.8677	24.01321
	std	**7.032006**	3.148419	4.880388	2.738736	5.899133	1.518094	4.62061	2.084239	6.560785	0.705619	2.041764	6.1097	2.014154
	median	**5.687176**	16.11758	12.23424	19.80712	8.262244	16.73777	12.82936	12.87313	12.41568	17.02748	20.08139	17.03368	20.82173
	rank	**1**	7	4	12	2	9	5	6	3	10	11	8	13
C11-F2	mean	**−26.3179**	−15.9138	−21.7194	−13.4565	−25.2481	−13.2117	−19.6074	−11.0437	−23.1085	−12.8688	−16.9203	−23.1512	−14.6421
	best	**−27.0676**	−16.9621	−22.2939	−13.8985	−25.85	−16.3476	−22.4632	−12.8537	−24.8167	−14.0126	−21.4324	−24.3687	−16.5512
	worst	**−25.4328**	−14.9089	−21.1364	−13.0127	−24.0741	−11.3447	−16.2291	−9.68473	−19.9571	−11.8322	−13.3466	−21.1826	−13.2755
	std	**0.722057**	1.072936	0.501119	0.475801	0.857781	2.413417	3.328862	1.440992	2.237383	0.930503	3.745006	1.408354	1.621478
	median	**−26.3856**	−15.8921	−21.7236	−13.4575	−25.5342	−12.5772	−19.8687	−10.8182	−23.8302	−12.8153	−16.4511	−23.5268	−14.3708
	rank	**1**	8	5	10	2	11	6	13	4	12	7	3	9
C11-F4	mean	**1.15 × 10^−5^**	1.15 × 10^−5^	1.15 × 10^−5^	1.15 × 10^−5^	1.15 × 10^−5^	1.15 × 10^−5^	1.15 × 10^−5^	1.15 × 10^−5^	1.15 × 10^−5^	1.15 × 10^−5^	1.15 × 10^−5^	1.15 × 10^−5^	1.15 × 10^−5^
	best	**1.15 × 10^−5^**	1.15 × 10^−5^	1.15 × 10^−5^	1.15 × 10^−5^	1.15 × 10^−5^	1.15 × 10^−5^	1.15 × 10^−5^	1.15 × 10^−5^	1.15 × 10^−5^	1.15 × 10^−5^	1.15 × 10^−5^	1.15 × 10^−5^	1.15 × 10^−5^
	worst	**1.15 × 10^−5^**	1.15 × 10^−5^	1.15 × 10^−5^	1.15 × 10^−5^	1.15 × 10^−5^	1.15 × 10^−5^	1.15 × 10^−5^	1.15 × 10^−5^	1.15 × 10^−5^	1.15 × 10^−5^	1.15 × 10^−5^	1.15 × 10^−5^	1.15 × 10^−5^
	std	**1.95 × 10^−19^**	1.87 × 10^−11^	2.14 × 10^−9^	4.2 × 10^−11^	1.05 × 10^−15^	2.01 × 10^−14^	4.9 × 10^−19^	8.38 × 10^−13^	3.14 × 10^−15^	6.6 × 10^−14^	1.69 × 10^−19^	6.42 × 10^−20^	2.31 × 10^−18^
	median	**1.15 × 10^−5^**	1.15 × 10^−5^	1.15 × 10^−5^	1.15 × 10^−5^	1.15 × 10^−5^	1.15 × 10^−5^	1.15 × 10^−5^	1.15 × 10^−5^	1.15 × 10^−5^	1.15 × 10^−5^	1.15 × 10^−5^	1.15 × 10^−5^	1.15 × 10^−5^
	rank	**1**	11	13	12	6	8	4	10	7	9	3	2	5
C11-F4	mean	**0**	0	0	0	0	0	0	0	0	0	0	0	0
	best	**0**	0	0	0	0	0	0	0	0	0	0	0	0
	worst	**0**	0	0	0	0	0	0	0	0	0	0	0	0
	std	**0**	0	0	0	0	0	0	0	0	0	0	0	0
	median	**0**	0	0	0	0	0	0	0	0	0	0	0	0
	rank	**1**	1	1	1	1	1	1	1	1	1	1	1	1
C11-F5	mean	**−34.1274**	−26.0703	−28.9288	−21.8701	−33.3926	−28.0835	−28.5154	−27.9624	−31.9229	−13.9037	−28.2712	−12.0273	−12.7727
	best	**−34.7494**	−27.0714	−29.9445	−23.7379	−33.9826	−31.8021	−28.7	−32.0647	−34.0666	−15.6486	−31.8924	−15.1159	−14.023
	worst	**−33.3862**	−25.2709	−28.4411	−19.8332	−32.2519	−23.5537	−28.0652	−25.7367	−28.4426	−12.5761	−25.4354	−10.552	−11.4111
	std	**0.576513**	0.784839	0.712623	2.17301	0.797135	3.492912	0.30949	3.012029	2.490682	1.348016	2.897875	2.196324	1.205573
	median	**−34.1871**	−25.9695	−28.6647	−21.9546	−33.668	−28.489	−28.6481	−27.0241	−32.5912	−13.6952	−27.8785	−11.2207	−12.8284
	rank	**1**	9	4	10	2	7	5	8	3	11	6	13	12
C11-F6	mean	**−24.1119**	−15.3943	−19.7225	−14.5328	−22.8219	−9.77998	−20.5213	−11.488	−20.2419	−5.23929	−22.1938	−5.98984	−6.77572
	best	**−27.4298**	−15.7454	−21.3912	−14.9609	−25.9832	−17.4134	−22.9922	−18.1843	−23.0902	−5.96024	−26.1223	−8.96244	−11.1447
	worst	**−23.0059**	−15.0486	−18.0255	−13.5029	−21.5594	−6.79967	−14.3118	−4.99898	−18.6664	−4.99898	−19.1024	−4.99898	−4.99898
	std	**2.271847**	0.377713	1.521189	0.708141	2.185792	5.265013	4.285644	7.160496	2.151891	0.493651	3.158433	2.035421	3.027548
	median	**−23.0059**	−15.3917	−19.7367	−14.8337	−21.8724	−7.45343	−22.3905	−11.3843	−19.6054	−4.99898	−21.7752	−4.99898	−5.47961
	rank	**1**	7	6	8	2	10	4	9	5	13	3	12	11
C11-F7	mean	**0.860699**	1.502351	1.225146	1.772504	0.920045	1.240506	1.620768	0.878234	1.038737	1.599303	1.049111	1.086923	1.618042
	best	**0.582266**	1.411009	1.079851	1.582205	0.732937	1.052704	1.518444	0.841591	0.843875	1.452557	0.842345	0.854143	1.242676
	worst	**1.025027**	1.620161	1.365036	1.955283	1.008514	1.572375	1.793033	0.9401	1.231193	1.744715	1.219762	1.293277	1.812241
	std	**0.206672**	0.092172	0.160099	0.156595	0.129573	0.235055	0.123128	0.044511	0.162482	0.133354	0.172852	0.221148	0.266994
	median	**0.91775**	1.489116	1.227848	1.776264	0.969365	1.168472	1.585796	0.865622	1.039941	1.59997	1.067169	1.100137	1.708625
	rank	**1**	9	7	13	3	8	12	2	4	10	5	6	11
C11-F8	mean	**220**	276.1359	237.6892	311.0486	222.1133	252.2281	259.8008	223.5222	226.3399	223.5222	242.7658	435.5821	222.1525
	best	**220**	253.2495	223.1308	275.6506	220	220	241.8376	220	220	220	220	244.264	220
	worst	**220**	306.1606	252.2476	349.6166	224.2266	336.2323	299.6015	234.0888	232.6799	234.0888	283.3994	522.4314	228.6098
	std	**0**	23.79358	12.86933	31.16122	2.506357	57.84678	27.4653	7.235229	7.519071	7.235229	30.87915	135.2212	4.421529
	median	**220**	272.5668	237.6892	309.4637	222.1133	226.3399	248.882	220	226.3399	220	233.8319	487.8164	220
	rank	**1**	10	6	11	2	8	9	4	5	4	7	12	3
C11-F9	mean	**8789.286**	483,074.6	328,388.4	919,557.3	18,656.81	58,460.01	325,218	116,528.7	38,391.85	354,583.9	713,172.7	937,253.5	1,681,148
	best	**5457.674**	324,045.2	290,082.5	601,645.3	10,517.11	41,850.42	180,313.9	66,460.77	16,926.42	293,497.2	611,204	752,356.9	1,611,892
	worst	**14,042.29**	554,409.1	353,198.3	1,078,156	25,556.09	73,839.47	550,072.5	176,575.1	65,752.91	455,337	767,456.3	1,148,459	1,779,466
	std	**3800.348**	111,655	28,458.98	221,949	7021.667	14,112.97	172,929.7	46,690.19	21,194.23	73,247.74	71,385.83	217,154.4	84,994.93
	median	**7828.591**	526,922	335,136.4	999,213.8	19,277.02	59,075.07	285,242.7	111,539.5	35,444.03	334,750.6	737,015.2	924,099	1,666,617
	rank	**1**	9	7	11	2	4	6	5	3	8	10	12	13
C11-F10	mean	**−21.4889**	−14.9961	−17.5447	−13.5346	−19.3631	−15.3584	−14.0482	−15.6278	−15.1114	−12.678	−14.2904	−12.7642	−12.5089
	best	**−21.8299**	−16.0597	−17.7395	−13.9161	−19.746	−19.2378	−14.5308	−21.2538	−15.5783	−12.7902	−14.7804	−12.8378	−12.5968
	worst	**−20.7878**	−14.5	−17.1211	−13.3132	−18.9255	−13.3665	−13.6616	−12.8466	−14.1286	−12.4989	−13.6383	−12.6297	−12.346
	std	**0.487227**	0.736752	0.299069	0.289239	0.407904	2.72605	0.369057	3.909365	0.695109	0.132631	0.590486	0.094807	0.114197
	median	**−21.669**	−14.7122	−17.659	−13.4545	−19.3905	−14.4147	−14.0001	−14.2053	−15.3695	−12.7115	−14.3714	−12.7947	−12.5465
	rank	**1**	7	3	10	2	5	9	4	6	12	8	11	13
C11-F11	mean	**571,712.3**	5,089,428	933,728.2	7,729,839	1,510,643	5,212,407	1,127,503	1,207,727	3,388,503	4,577,612	1,296,573	4,587,180	5,366,107
	best	**260,837.9**	4,846,157	702,522.4	7,431,600	1,392,282	4,341,728	1,021,968	624,656.8	3,211,626	4,510,062	1,160,011	4,529,197	5,286,309
	worst	**828,560.9**	5,424,830	1,124,700	7,928,857	1,662,178	6,302,843	1,302,398	2,427,751	3,655,171	4,633,423	1,466,471	4,633,423	5,417,883
	std	**254,962.4**	288,930.1	189,236.6	216,947.1	138,529.7	833,226.9	130,660.1	845,509.3	193,819.7	55,400.98	134,407.8	51,634.41	59,799.43
	median	**598,725.2**	5,043,362	953,845.3	7,779,449	1,494,055	5,102,528	1,092,822	889,249.9	3,343,608	4,583,482	1,279,905	4,593,050	5,380,117
	rank	**1**	10	2	13	6	11	3	4	7	8	5	9	12
C11-F12	mean	**1,199,805**	7,409,804	3,078,638	11,593,820	1,264,353	4,507,023	5,185,186	1,310,025	1,393,451	12,537,698	5,163,454	2,161,837	12,676,663
	best	**1,155,937**	7,106,082	2,984,012	10,776,140	1,194,678	4,281,046	4,827,396	1,182,719	1,247,427	11,817,734	4,917,229	2,021,679	12,560,401
	worst	**1,249,353**	7,682,492	3,144,051	12,315,917	1,339,516	4,626,721	5,361,089	1,428,430	1,519,843	13,096,880	5,338,627	2,335,962	12,793,601
	std	**46,080.46**	244,867.2	71,787.57	648,639.6	66,020.2	164,278.3	252,560.4	103,468	116,316.4	550,537.4	185,730.7	133,330.1	98,177.52
	median	**1,196,965**	7,425,322	3,093,245	11,641,612	1,261,610	4,560,163	5,276,130	1,314,475	1,403,267	12,618,090	5,198,980	2,144,854	12,676,324
	rank	**1**	10	6	11	2	7	9	3	4	12	8	5	13
C11-F13	mean	**15,444.2**	15,801.46	15,447.48	16,192.64	15,460.59	15,483.94	15,523.07	15,499.24	15,493.37	15,866.74	112,999.3	15,484.49	28,024.66
	best	**15,444.19**	15,641.17	15,446.62	15,833.03	15,458.6	15,475.37	15,485.31	15,481.76	15,487.39	15,602.6	82,270.86	15,469.61	15,458.24
	worst	**15,444.21**	16,189.79	15,448.43	17,086.06	15,464.04	15,494.84	15,573.96	15,532.51	15,503.73	16,353.34	154,677.9	15,516.05	65,420.01
	std	**0.008884**	268.4869	0.785818	616.7849	2.478024	9.886368	42.36283	24.1668	7.436411	349.0008	33,482.43	21.84165	25,605.76
	median	**15,444.2**	15,687.44	15,447.43	15,925.73	15,459.86	15,482.78	15,516.5	15,491.35	15,491.19	15,755.51	107,524.3	15,476.15	15,610.2
	rank	**1**	9	2	11	3	4	8	7	6	10	13	5	12
C11-F14	mean	**18,295.35**	99,701.16	18,488.97	200,495.8	18,565.39	19,363.59	19,099.47	19,266.84	19,105.47	271,120.5	18,984.5	19,012.65	19,001.62
	best	**18,241.58**	76,442.57	18,382.24	148,310.8	18,484.84	19,143.07	18,958.94	19,171.86	18,971.35	28,642.2	18,736.06	18,872.44	18,757.13
	worst	**18,388.08**	138,457.2	18,586.71	287,843.8	18,643.92	19,830.86	19,196.33	19,330.18	19,258.69	520,996.6	19,158.54	19,151.92	19,253.43
	std	**69.96398**	28,491.57	97.99008	64,196.31	68.36705	323.4255	115.9594	71.45986	130.9626	242,775.2	191.4042	117.2667	208.7133
	median	**18,275.87**	91,952.46	18,493.46	182,914.4	18,566.4	19,240.21	19,121.31	19,282.67	19,095.92	267,421.6	19,021.71	19,013.11	18,997.96
	rank	**1**	11	2	12	3	10	7	9	8	13	4	6	5
C11-F15	mean	**32,883.58**	789,854	97,714.07	1,660,569	32,939.59	51,625.11	193,517.4	33,070.75	33,051.61	13,341,620	263,714.8	33,233.38	6,868,518
	best	**32,782.17**	328,154.6	41,791.18	697,019.4	32858	33,035.37	32,978.67	32,983.94	33,009.67	2,799,046	233,893.6	33,217.56	3,128,721
	worst	**32,956.46**	1,979,409	160,296.7	4,327,558	33,010.26	107,181.4	275,074.9	33,122.91	33,117.85	19,893,221	284,122.7	33,253.24	11,768,149
	std	**75.18941**	817,470.1	65,423.85	1,829,004	64.17747	38,041.05	112,257.6	63.22443	49.31413	7,983,297	24,000.85	15.28513	4,068,609
	median	**32,897.86**	425,926.1	94,384.21	808,848.4	32,945.06	33,141.84	233,008	33,088.08	33,039.45	15,337,106	268,421.4	33,231.35	6,288,601
	rank	**1**	10	7	11	2	6	8	4	3	13	9	5	12
C11-F16	mean	**133,550**	835,504.6	134,957	1,703,962	137,102.1	143,561.1	140,998.8	140,691.3	144,206.2	76,907,410	16,210,894	68,837,661	66,096,332
	best	**131,374.2**	268,051.3	133,344.2	427,899.5	135,005.9	140,925.2	136,081.1	133,384.9	142,154.2	74,944,479	8,242,318	56,944,849	53,423,145
	worst	**136,310.8**	1,949,599	135,633	4,208,326	140,662.2	145,270.7	145,978.3	148,000	149,364.9	79,121,321	29,314,317	82,255,889	84,537,318
	std	**2337.559**	776,901.4	1116.913	1,746,392	2587.54	2086.663	4238.576	6256.899	3555.112	1,797,875	9,358,325	11,205,354	13,574,898
	median	**133,257.5**	562,184	135,425.5	1,089,810	136,370.1	144,024.3	140,967.9	140,690.3	142,653	76,781,921	13,643,470	68,074,953	63,212,432
	rank	**1**	8	2	9	3	6	5	4	7	13	10	12	11
C11-F17	mean	**1,926,615**	7.75 × 10^9^	2 × 10^9^	1.34 × 10^10^	2,241,719	1.11 × 10^9^	8.39 × 10^9^	2,951,922	2,871,926	1.93 × 10^10^	9.7 × 10^9^	1.8 × 10^10^	1.89 × 10^10^
	best	**1,916,953**	6.6 × 10^9^	1.82 × 10^9^	9.64 × 10^9^	1,951,893	9.14 × 10^8^	5.98 × 10^9^	2,248,940	2,021,775	1.86 × 10^10^	8.53 × 10^9^	1.59 × 10^10^	1.77 × 10^10^
	worst	**1,942,685**	8.59 × 10^9^	2.19 × 10^9^	1.64 × 10^10^	2,773,823	1.27 × 10^9^	1.11 × 10^10^	3,497,374	4,480,830	2.01 × 10^10^	1.03 × 10^10^	2.08 × 10^10^	2.14 × 10^10^
	std	**11,729.35**	9.04 × 10^8^	1.68 × 10^8^	2.98 × 10^9^	378,232.3	1.87 × 10^8^	2.23 × 10^9^	591,769.5	1,137,387	6.69 × 10^8^	8.12 × 10^8^	2.28 × 10^9^	1.72 × 10^9^
	median	**1,923,412**	7.9 × 10^9^	2 × 10^9^	1.38 × 10^10^	2,120,579	1.13 × 10^9^	8.2 × 10^9^	3,030,686	2,492,549	1.92 × 10^10^	9.99 × 10^9^	1.77 × 10^10^	1.83 × 10^10^
	rank	**1**	7	6	10	2	5	8	4	3	13	9	11	12
C11-F18	mean	**942,057.5**	47,685,736	5,797,182	1.03 × 10^8^	967,735.7	1,900,478	8,412,951	981,892.1	1,019,092	26,941,532	9,752,238	1.17 × 10^8^	99,204,327
	best	**938,416.2**	32,835,794	3,536,567	70,851,530	948,240.5	1,678,127	3,691,349	961,316.7	963,662	21,379,694	7,310,034	98,075,250	95,567,953
	worst	**944,706.9**	54,224,643	9,852,395	1.17 × 10^8^	1,018,468	2,196,439	14,673,571	991,091.1	1,165,991	29,133,515	12,269,456	1.3 × 10^8^	1.03 × 10^8^
	std	**2710.775**	10,287,738	3,021,014	22,213,838	34,862.01	256,626.5	4,762,937	14,247.92	100,799.7	3,824,041	2,275,875	14,522,917	3,058,517
	median	**942,553.5**	51,841,254	4,899,884	1.11 × 10^8^	952,117	1,863,672	7,643,442	987,580.2	973,357.6	28,626,459	9,714,731	1.2 × 10^8^	99,195,338
	rank	**1**	10	6	12	2	5	7	3	4	9	8	13	11
C11-F19	mean	**1,025,341**	46,951,626	5,895,982	1 × 10^8^	1,122,631	2,269,177	8,976,703	1,415,705	1,317,213	30,923,282	5,559,356	1.49 × 10^8^	99,546,536
	best	**967,927.7**	40,077,691	5,413,733	86,762,354	1,056,780	2,056,560	1,912,200	1,106,530	1,198,912	21,691,480	2,222,360	1.36 × 10^8^	97,066,099
	worst	**1,167,142**	59,652,101	7,107,091	1.26 × 10^8^	1,275,910	2,645,965	16,142,794	1,827,341	1,493,445	38,532,571	7,250,902	1.73 × 10^8^	1.02 × 10^8^
	std	**97,398.36**	9,066,953	834,136.7	18,873,011	105,641.6	265,805.2	6,885,212	308,560.8	128,320.4	7,489,918	2,343,343	16,563,540	2,301,701
	median	**983,146.6**	44,038,356	5,531,551	94,386,241	1,078,918	2,187,092	8,925,910	1,364,474	1,288,247	31,734,538	6,382,080	1.45 × 10^8^	99,313,659
	rank	**1**	10	7	12	2	5	8	4	3	9	6	13	11
C11-F20	mean	**941,250.4**	49,902,655	5,223,809	1.08 × 10^8^	957,767.4	1,706,979	6,424,884	968,550.5	990,801.4	30,030,029	12,470,747	1.38 × 10^8^	99,812,450
	best	**936,143.2**	43,921,348	4,620,095	94,897,156	955,705.6	1,548,205	6,060,356	960,209	973,383.7	29,375,031	8,325,455	1.26 × 10^8^	95,046,637
	worst	**946,866.6**	59,071,400	5,868,197	1.29 × 10^8^	958,850.4	1,972,240	6,911,234	977,985.8	1,004,265	30,738,864	19,234,959	1.5 × 10^8^	1.04 × 10^8^
	std	**4899.038**	6,630,787	532,074.1	14,882,069	1441.981	206,856.4	373,449.1	7851.209	13,658.84	582,763.3	4,896,556	13,558,271	3,674,975
	median	**940,995.9**	48,308,936	5,203,473	1.05 × 10^8^	958,256.7	1,653,735	6,363,973	968,003.6	992,778.4	30,003,110	11,161,287	1.38 × 10^8^	1 × 10^8^
	rank	**1**	10	6	12	2	5	7	3	4	9	8	13	11
C11-F21	mean	**12.71443**	45.1876	20.43993	67.87132	15.50132	27.51897	35.27695	25.54006	21.07511	88.83355	36.93059	93.18987	90.48226
	best	**9.974206**	37.79559	18.9035	51.24228	13.24809	24.4736	32.7261	22.6756	19.38568	43.73739	33.01343	80.97607	52.76258
	worst	**14.97499**	53.06318	22.32666	84.41698	17.78701	28.83146	38.45684	28.47066	23.41105	129.9077	39.66032	103.1089	109.7311
	std	**2.357559**	6.741994	1.502622	15.03798	2.158206	2.098538	2.590224	3.174898	1.907519	36.26977	3.001039	11.39521	27.27537
	median	**12.95425**	44.94581	20.26478	67.91302	15.48509	28.38542	34.96244	25.507	20.75185	90.84455	37.5243	94.33725	99.71769
	rank	**1**	9	3	10	2	6	7	5	4	11	8	13	12
C11-F22	mean	**16.12513**	42.68276	25.9215	57.10887	18.68324	29.98205	42.26705	30.11715	23.79947	90.96656	42.57617	94.45669	82.28708
	best	**11.50133**	37.81929	20.73719	42.43422	15.55659	25.85189	36.1813	23.32794	23.31157	59.34979	36.35534	78.91304	80.82874
	worst	**19.55286**	47.9384	30.93823	65.87537	21.03086	32.65023	46.82953	34.97092	24.23236	107.8169	50.02428	104.5544	84.13235
	std	**4.101915**	4.49598	4.977189	10.48875	2.703119	2.979601	4.988123	5.135703	0.41001	22.32621	5.84784	11.714	1.411088
	median	**16.72317**	42.48667	26.00529	60.06295	19.07277	30.71304	43.02869	31.08487	23.82698	98.34978	41.96253	97.17966	82.09361
	rank	**1**	9	4	10	2	5	7	6	3	12	8	13	11
Sum rank		**22**	191	109	231	55	146	145	118	97	222	157	198	224
Mean rank		**1**	8.681818	4.954545	10.5	2.5	6.636364	6.590909	5.363636	4.409091	10.09091	7.136364	9	10.18182
Total rank		**1**	2	12	4	13	3	11	9	6	7	10	5	8
Wilcoxon: *p*-value			1.28 × 10^−15^	7.32 × 10^−15^	1.28 × 10^−15^	5.32 × 10^−15^	2.74 × 10^−15^	1.28 × 10^−15^	2.99 × 10^−12^	5.32 × 10^−15^	4.02 × 10^−15^	6.38 × 10^−15^	1.90 × 10^−15^	4.02 × 10^−15^

**Table 8:** Performance of optimization algorithms on pressure vessel design problem.

Algorithm	Optimum Variables				Optimum Cost
	*T_s_*	*T_h_*	*R*	*L*	
POA	0.7780271	0.3845792	40.312284	200	**5882.8955**
WSO	0.7780272	0.3845788	40.312283	200	5882.9013
AVOA	0.7780307	0.384581	40.312469	199.99741	5882.9075
RSA	1.1799694	0.6311498	59.819101	53.515497	7692.0978
MPA	0.7780271	0.3845792	40.312284	200	5882.9013
TSA	0.7794463	0.3857743	40.383839	200	5908.4196
WOA	0.9067002	0.448673	46.017223	136.2267	6256.8632
MVO	0.8323916	0.4152568	43.112935	165.20916	5999.4855
GWO	0.7784443	0.3857682	40.320326	199.96571	5889.9468
TLBO	1.5339616	0.4778123	47.429558	127.36717	10,629.675
GSA	1.1173119	1.1297405	43.973024	191.12001	11,764.254
PSO	1.5222025	0.6145174	62.315801	55.20521	9850.1299
GA	1.3837426	0.7688899	57.605994	78.51226	10,738.52

**Table 9:** Statistical results of optimization algorithms on pressure vessel design problem.

Algorithm	Mean	Best	Worst	Std	Median	Rank
POA	**5882.8955**	**5882.8955**	**5882.8955**	**1.92 × 10^−12^**	**5882.8955**	**1**
WSO	5890.9257	5882.9013	5962.0728	21.997434	5882.9017	3
AVOA	6207.3916	5882.9075	7004.3405	348.84586	6041.7492	5
RSA	12,174.081	7692.0978	19,482.674	3095.8917	11,204.142	9
MPA	5882.9013	5882.9013	5882.9013	3.65 × 10^−6^	5882.9013	2
TSA	6257.1233	5908.4196	6909.9332	329.83718	6134.2078	6
WOA	7922.2943	6256.8632	12,555.602	1665.1308	7518.4095	8
MVO	6495.1818	5999.4855	7008.2785	317.12575	6547.3182	7
GWO	6007.6959	5889.9468	6642.5575	236.99908	5897.9847	4
TLBO	27,465.413	10,629.675	58,347.67	13,658.123	24,286.567	12
GSA	20,110.975	11,764.254	31,159.248	6644.7597	19,327.122	10
PSO	28,828.623	9850.1299	49,094.713	12,786.567	31,741.352	13
GA	24,722.523	10,738.52	44,100.29	10,720.864	21,949.806	11

**Table 10:** Performance of optimization algorithms on speed reducer design problem.

Algorithm	Optimum Variables							Optimum Cost
	b	*M*	*p*	*l*_1_	*l*_2_	*d*_1_	*d*_2_	
POA	3.5	0.7	17	7.3	7.8	3.3502147	5.2866832	**2996.3482**
WSO	3.5000004	0.7	17	7.3000084	7.8000004	3.3502148	5.2866833	2996.3483
AVOA	3.5	0.7	17	7.3000006	7.8	3.3502147	5.2866832	2996.3482
RSA	3.5782709	0.7	17	8.0827092	8.1913546	3.3548418	5.4536482	3154.7106
MPA	3.5	0.7	17	7.3	7.8	3.3502147	5.2866832	2996.3482
TSA	3.5109533	0.7	17	7.3	8.1913546	3.3504914	5.2896834	3011.2327
WOA	3.5742812	0.7	17	7.3	7.9777636	3.3598929	5.2867448	3031.9314
MVO	3.5019122	0.7	17	7.3	8.0284714	3.366672	5.2868519	3006.4419
GWO	3.5005445	0.7	17	7.3043676	7.8	3.3618765	5.2884893	3000.7349
TLBO	3.5476378	0.7033945	24.917693	7.9805293	8.0931464	3.6162006	5.3314146	4927.3723
GSA	3.5194552	0.7023381	17.313478	7.7420374	7.8760975	3.3999447	5.3709689	3143.5799
PSO	3.5069497	0.7000611	17.930439	7.3841039	7.8577718	3.5584711	5.3353772	3256.3667
GA	3.5662501	0.7047261	17.691104	7.6758437	7.8474233	3.6485809	5.3373389	3294.0025

**Table 11:** Statistical results of optimization algorithms on speed reducer design problem.

Algorithm	Mean	Best	Worst	Std	Median	Rank
POA	**2996.3482**	**2996.3482**	**2996.3482**	**9.58 × 10^−13^**	**2996.3482**	**1**
WSO	2996.5889	2996.3483	2998.4297	0.5101165	2996.3619	3
AVOA	3000.1762	2996.3482	3008.8545	3.4609637	3000.0917	4
RSA	3234.4969	3154.7106	3284.0135	50.166314	3247.1314	9
MPA	2996.3482	2996.3482	2996.3482	2.78 × 10^−6^	2996.3482	2
TSA	3026.7367	3011.2327	3038.3972	8.8440577	3028.2551	7
WOA	3126.8766	3031.9314	3377.445	92.715236	3098.5654	8
MVO	3024.7753	3006.4419	3059.0455	11.563388	3025.1488	6
GWO	3003.374	3000.7349	3008.4394	2.1869003	3002.9342	5
TLBO	5.907 × 10^13^	4927.3723	4.275 × 10^14^	1.01 × 10^14^	2.313 × 10^13^	12
GSA	3385.5422	3143.5799	3912.9273	228.70017	3275.4427	10
PSO	8.717 × 10^13^	3256.3667	4.416 × 10^14^	1.081 × 10^14^	6.235 × 10^13^	13
GA	4.197 × 10^13^	3294.0025	2.709 × 10^14^	6.79 × 10^13^	1.682 × 10^13^	11

**Table 12:** Performance of optimization algorithms on welded beam design problem.

Algorithm	Optimum Variables				Optimum Cost
	*h*	*l*	*t*	*b*	
POA	0.2057296	3.4704887	9.0366239	0.2057296	**1.7246798**
WSO	0.2057292	3.4704885	9.0366237	0.2057291	1.7248523
AVOA	0.2050803	3.4845704	9.0365333	0.2057338	1.7257584
RSA	0.1980596	3.5249794	9.7906672	0.2159744	1.9375828
MPA	0.2057296	3.4704887	9.0366239	0.2057296	1.7248523
TSA	0.2044275	3.4916172	9.0600241	0.2060919	1.7324856
WOA	0.2125196	3.3510058	8.9833094	0.2186902	1.8067407
MVO	0.2059533	3.4656684	9.0434674	0.2060063	1.7278338
GWO	0.2056128	3.4731682	9.0362981	0.2057883	1.7254222
TLBO	0.2986976	4.2778023	7.1361343	0.3919305	2.8272527
GSA	0.2805172	2.8349124	7.6654232	0.2924906	2.0300996
PSO	0.3473169	3.4316063	7.6004377	0.5182734	3.675343
GA	0.2214997	6.3937785	7.9559315	0.2894528	2.6042749

**Table 13:** Statistical results of optimization algorithms on welded beam design problem.

Algorithm	Mean	Best	Worst	Std	Median	Rank
POA	**1.7246798**	**1.7246798**	**1.7246798**	**2.34 × 10^−16^**	**1.7246798**	**1**
WSO	1.7248526	1.7248523	1.724857	1.09 × 10^−6^	1.7248523	3
AVOA	1.7557138	1.7257584	1.8248581	0.0317906	1.7439234	7
RSA	2.1125267	1.9375828	2.4075717	0.1256523	2.0912766	8
MPA	1.7248523	1.7248523	1.7248523	2.92 × 10^−9^	1.7248523	2
TSA	1.7403814	1.7324856	1.7481834	0.0048867	1.7404631	6
WOA	2.2220918	1.8067407	3.6950008	0.5593985	2.031176	9
MVO	1.7387463	1.7278338	1.7674553	0.0119923	1.7352918	5
GWO	1.7268895	1.7254222	1.7303217	0.001188	1.7266813	4
TLBO	2.823 × 10^13^	2.8272527	2.724 × 10^14^	7.072 × 10^13^	5.0914359	12
GSA	2.3349608	2.0300996	2.5968573	0.1669487	2.3600351	10
PSO	3.893 × 10^13^	3.675343	2.357 × 10^14^	7.636 × 10^13^	5.9728379	13
GA	9.556 × 10^12^	2.6042749	1.034 × 10^14^	3.013 × 10^13^	5.0630857	11

**Table 14:** Performance of optimization algorithms on tension/compression spring design problem.

Algorithm	Optimum Variables			Optimum Cost
	*d*	*D*	*P*	
POA	0.0516891	0.3567177	11.288966	**0.0126019**
WSO	0.0516874	0.3566773	11.291337	0.0126652
AVOA	0.0512669	0.3466708	11.910602	0.0126694
RSA	0.050367	0.3206032	14.193627	0.0130834
MPA	0.0516905	0.3567522	11.286949	0.0126652
TSA	0.0510947	0.3426132	12.187613	0.0126794
WOA	0.0512453	0.3461621	11.94391	0.0126699
MVO	0.050367	0.325522	13.492324	0.0127369
GWO	0.0519158	0.3621859	10.980498	0.0126699
TLBO	0.0653037	0.8107583	4.0184069	0.0167497
GSA	0.0545928	0.4283484	8.3456483	0.0130117
PSO	0.0652338	0.8081171	4.0184069	0.0166633
GA	0.0657002	0.8173757	4.0184069	0.0170839

**Table 15:** Statistical results of optimization algorithms on tension/compression spring design problem.

Algorithm	Mean	Best	Worst	Std	Median	Rank
POA	**0.0126019**	**0.0126019**	**0.0126019**	**7.07 × 10^−18^**	**0.0126019**	**1**
WSO	0.0126746	0.0126652	0.0127999	3.084 × 10^−5^	0.0126656	3
AVOA	0.0132328	0.0126694	0.0139111	0.0004795	0.0131756	8
RSA	0.0131519	0.0130834	0.0132725	5.968 × 10^−5^	0.0131343	6
MPA	0.0126652	0.0126652	0.0126652	2.45 × 10^−9^	0.0126652	2
TSA	0.0129142	0.0126794	0.0133863	0.0002078	0.0128525	5
WOA	0.0131737	0.0126699	0.014201	0.0005197	0.0130077	7
MVO	0.0158555	0.0127369	0.0170594	0.0014169	0.0166225	9
GWO	0.0127136	0.0126699	0.0129006	4.757 × 10^−5^	0.0127115	4
TLBO	0.0171949	0.0167497	0.0177033	0.0003079	0.0171578	10
GSA	0.0183255	0.0130117	0.0289135	0.003664	0.0179686	11
PSO	1.752 × 10^13^	0.0166633	3.109 × 10^14^	7.145 × 10^13^	0.0166633	13
GA	1.369 × 10^12^	0.0170839	1.416 × 10^13^	4.198 × 10^12^	0.0234606	12

## Data Availability

Data are contained within the article.

## Appendix A

The optimal value (OV) for each test function of the CEC 2017 test suite is presented in Table A1. It is also explained in Appendix A. Also, information about the optimal values of the CEC 2011 test suite is given in reference [69].

**Table A1:** The optimal values of CEC 2017 test suite.

function	C17-F1	C17-F2	C17-F3	C17-F4	C17-F5	C17-F6	C17-F7	C17-F8	C17-F9	C17-F10	C17-F11	C17-F12	C17-F13	C17-F14	C17-F15
OV	100	200	300	400	500	600	700	800	900	1000	1100	1200	1300	1400	1500
function	C17-F16	C17-F17	C17-F18	C17-F19	C17-F20	C17-F21	C17-F22	C17-F23	C17-F24	C17-F25	C17-F26	C17-F27	C17-F28	C17-F29	C17-F30
OV	1600	1700	1800	1900	2000	2100	2200	2300	2400	2500	2600	2700	2800	2900	3000
